# TRIM24 promotes proliferation and metastasis of gastric cancer via mediating NRBP1 ubiquitination

**DOI:** 10.1038/s41419-025-08346-w

**Published:** 2025-12-22

**Authors:** Chunyan Weng, Jingli Xu, Chenghai He, Rijuan Jin, Xiaoliang Jin, Shaopeng Sun, Siwei Pan, Meng Li, Yue Hu, Xi Wang, Yanqiang Zhang, Can Hu, Zhiyuan Xu, Bin Lv

**Affiliations:** 1 Department of Gastroenterology, The First Affiliated Hospital of Zhejiang Chinese Medical University (Zhejiang Provincial Hospital of Chinese Medicine), Hangzhou, Zhejiang China; 2 Department of Gastric surgery, Zhejiang Cancer Hospital, Hangzhou, Zhejiang China; 3 Department of Internal Medicine, The Affiliated Hospital of Hangzhou Normal University, Hangzhou, Zhejiang China; 4 Key Laboratory of Digestive Pathophysiology of Zhejiang Province, Hangzhou, Zhejiang China

**Keywords:** Gastric cancer, Prognostic markers

## Abstract

The early detection and precise treatment of gastric cancer (GC) remain critical challenges worldwide. In this work, we screened and identified a subset of highly aggressive GC cell lines that exhibit elevated expression of TRIM24 using transwell assays and animal models. TRIM24 showed enhanced expression in GC cells and gastric carcinoma tissue samples in comparison with gastric noncancerous tissues. Importantly, elevated TRIM24 levels correlated with advanced tumor stage and poorer clinical outcomes. Functionally, TRIM24 acted as an oncogene, driving GC proliferation, invasion, and metastasis both in cell culture and animal experiments. Notably, TRIM24 knockdown markedly inducted apoptosis in GC cells through the modulation of NRBP1, a known context-specific tumor suppressor. Mechanistically, TRIM24 bound to NRBP1, enhancing its ubiquitination and subsequent degradation. Further mechanistic insights revealed that NRBP1 phosphorylation at residue S42 was crucial for TRIM24-mediated ubiquitination, with residue K430 identified as the specific ubiquitination site targeted by TRIM24. Jointly, the above findings unveil a critical role for TRIM24 in GC tumorigenesis and metastatic progression, thereby positioning TRIM24 as a promising therapeutic target in GC management.

## Introduction

Gastric cancer (GC) represents an important malignancy and a leading cause of cancer mortality worldwide. The most recent estimates released by GLOBOCAN reported about 0.98 million incident gastric cancer cases globally in 2022 [1]. Due to the limited availability of early screening for GC, diagnosis is mostly performed at an advanced stage, potentially with distant metastases, thereby forfeiting opportunities for surgical intervention [2]. Although GC incidence and mortality have markedly declined in recent decades, 5-year survival rate in GC cases remains notably low [3]. This may be attributed to poor chemotherapy tolerance, insensitivity to immunotherapy, and limited options in targeted therapies for GC treatment [4]. Hence, exploring the mechanisms underpinning GC development is urgently needed, as well as the discovery of new therapeutic targets.

The liver, peritoneum, distant lymph nodes (LNs), and bones are the most frequent sites for metastasis in GC [5]. Liver metastasis from gastric cancer can occur due to the anatomical proximity between the stomach and liver, facilitated by the liver’s distinct blood supply and hemodynamic architecture [6]. Peritoneal metastasis (PM) occurs in ∼53–66% of patients with distant metastatic GC [7, 8]. The pathogenesis of peritoneal metastasis in GC is very complex, involving adhesion, movement, angiogenesis, immune evasion, etc [9]. Moreover, bone metastases in gastric cancer are uncommon and typically emerge in the later stages of the disease’s progression; however, micrometastatic seeding in the bone may be evident in the early stage of the disease [10]. Overall, once metastasis occurs, the survival rate of GC patients significantly declines. Thus, identifying effective therapeutic targets becomes crucial.

Protein ubiquitination represents an important and dynamic post-translational modification utilized by almost all eukaryotes to modulate cellular events, e.g., cell cycle, cell signaling transduction, transcriptional regulation [11–16]. Dysregulation of this ubiquitination process is involved in the pathogenetic mechanisms of numerous diseases, notably multiple forms of cancer [17]. During ubiquitination, ubiquitin’s C terminus forms a bond with the ɛ-amino group of a lysine on the target protein [18]. The ubiquitin conjugation process typically requires the joint activity of ubiquitin-activating enzyme (E1), ubiquitin-conjugating enzyme (E2), and ubiquitin ligase (E3) [19]. Specifically, E1 induces ubiquitin activation; E2 accepts ubiquitin molecules from E1 and, in collaboration with E3, ultimately transfers the ubiquitin molecule to its substrate protein [20]. Notably, E3 determines the specificity of the ubiquitin system, with different E3 ligases exhibiting targeting specificities for particular proteins [21]. Extensive research suggests E3 ligases play crucial roles in cancer progression, a phenomenon attributable to their modulatory effects on proteins controlling diverse cell events. An expanding body of research connects ubiquitination to tumor metastasis, with evidence suggesting its pivotal involvement in cellular signaling pathways and various biological functions within the microenvironment [18, 22, 23].

This study identifies TRIM24 as an E3 ubiquitin ligase that is closely associated with the progression and metastasis of GC. We further examined TRIM24 expression in GC, and identified its oncogenic functions in cell culture and animal studies. Moreover, the specific mechanism through which TRIM24 promotes malignancy in GC was elucidated. These findings present novel therapeutic targets and potential avenues for GC therapy.

## Materials and methods

### Cell lines and culture

HEK293T cell, Human normal gastric epithelial NGEC and GC (FU97, AGS, NUGC3, MKN-74, NCI-N87, BGC823, MKN-1, NUGC4, MKN-45, MKN-7 and GCIY) cells were provided by the Type Culture Collection of the Chinese Academy of Science (Shanghai, China) and cultured in our laboratory. All cells were recently authenticated by STR profiling. Cell culture was carried out with RPMI 1640 or Dulbecco’s Modified Eagle Medium (DMEM) (Gibco, USA) with 10% fetal bovine serum (FBS; Gibco), 100 U/mL penicillin, and 100 μg/mL streptomycin (Invitrogen, USA) in a humid incubator with 5% CO2 at 37 °C. All the cells used in this study were tested for mycoplasma.

### Construction of aggressive GC cell line

We developed highly aggressive GC cells derived from the liver-metastatic GC cell line, MKN74. Briefly, MKN74 (5 × 10^5^) cells were seeded into the upper chamber of a Transwell insert (Corning, cat# 3452). After 2 days, the cells that had migrated to the lower chamber were collected and cultured. This process was repeated three times. The collected MKN74 cells (1 × 10^6^) were then subcutaneously injected into nude mice. Fourteen days later, the subcutaneous tumors were implanted into the greater curvature of the mice’s stomachs. After 1 month, tumors that had metastasized to the liver were harvested, dissociated into single cells, and further cultured. This process was also repeated three times. Finally, RNA sequencing was performed to compare the transcriptomes of the MKN74-aggressive cells with the parental MKN74 cells.

### Bioinformatics and RNA sequencing

Differential expression of TRIM24 between GC and adjacent noncancerous specimens was assessed with the Gene Expression Profiling Interactive Analysis (GEPIA; http://gepia.cancer-pku.cn/) database. GEPIA was utilized for the evaluation of the correlation between TRIM24 mRNA levels and GC patient prognosis. 29 paired GC tumor and paired cancerous tissues were collected from Zhejiang cancer hospital. Following that, total RNAs were collected and sent to GeneChem Biotechnology Company (Shanghai, China) for mRNA library construction and sequencing. TRIM family mRNAs were selected for further analysis and differentially expressed mRNAs were selected using edgeR with FDR < 0.05 and |log2FC| > 1.

### Patients’ tissue specimens and tissue microarray (TMA) construction

Primary GC and paired adjacent noncancerous tissue specimens were collected from 101 individuals surgically resected in the First Affiliated Hospital of Zhejiang Chinese Medicine University from 2008 to 2010. Relative complete clinical data were available in 78 patients. Each patient provided signed informed consent. TMA construction was carried out with 101 GC and 74 noncancerous tissue samples. For each sample, staining was performed with antibodies targeting TRIM24 and/or NRBP1, followed by incubation with Goat anti-Rabbit IgG H&L (HRP). The list of antibodies is available in Table S1. Protein expressions were determined on an H-score system. H-scores were derived from the following formula: H-score = ∑ (IS × AP), where IS is staining intensity (0 to 3 indicating no, weak, intermediate and strong staining, respectively) and AP is the percent of positive cancer or epithelial cells (0–4 indicating <5%, 5–25%, 26–50%, 51–75% and 76–100%, respectively), resulting in total H-scores ranging from 0 to 12. Furthermore, TRIM24 expression levels were grouped based on median IHC staining score: low TRIM24 expression (IHC staining score <10), and high TRIM24 expression (IHC staining score ≥10). The clinicopathological characteristics were verified by two pathologists with extensive experience. No patient had undergone preoperative chemotherapy or radiation therapy. Informed consent was obtained from all human subjects prior to their participation in the study. Ethical approval was obtained from the First Affiliated Hospital of Zhejiang Chinese Medicine University (approval number: IRB-2023-960(IIT)).

### Western blot assay

Protein purification from cell and tissue samples used a lysis buffer with protease inhibitors (Bimake). After SDS-PAGE, the samples underwent electro-transfer onto a PVDF membrane (Millipore), followed by blocking (5% skim milk in PBST) and successive incubations with primary and secondary antibodies. Detection was carried out with an enhanced chemiluminescent substrate kit (Fude). For the validation of the kinase responsible for the phosphorylation at the S42 site of NRBP1, HEK293T cells were treated with the CK1 inhibitor D4476 (MCE, Cat. No# HY-10324) for 6 h, followed by immunoblotting analysis. The details of relevant antibodies are listed in Supplementary Table S1.

### Lentivirus infections

We purchased lentiviruses harboring an shRNA against TRIM24, a TRIM24 overexpression plasmid, or a scramble shRNA from Shanghai GeneChem Co Ltd, China. ShRNAs against TRIM24 were CCGATCCCAAGCTCATCATTT and GCGCCTCCTTAAAGTTGCCAT. Infections were carried out as directed by the manufacturer for 72 h, and transfection efficacy was determined by western blot. For TRIM24 knockout, we employed a CRISPR-Cas9 system with a single guide RNA (sgRNA) targeting the TRIM24 locus (sequence: ACACGGCGCAAGTGTCCAA), and the corresponding lentivirus was constructed by Shanghai GeneChem Co., Ltd, China.

### TRIM24 inhibitor treatment

NUGC3 GC cells were treated with 1 μM IACS-9571 (MCE, cat#HY-102000B) and 0.5 μM dTRIM24 (MCE, cat#HY-111519) for 24 h. Following the treatment, the cells were subjected to western blot, clone formation, and transwell assays.

### Quantitative proteome analysis

GeneChem Biotechnology Company (China) constructed quantitative proteome libraries and performed sequencing with stable knockdown TRIM24 GC and vector GC cells. Next, proteins were extracted, quantitated and enzymatically hydrolyzed, followed by mass spectroscopy (MS). MS data analysis used MaxQuant 1.6.17.0. The cutoff of the global FDR for identifying peptides and proteins was 0.01. Protein abundance was determined according to normalized spectral protein intensity (LFQ). Each protein with a fold change above 1.5 and Student’s t-test P < 0.05 was deemed to show differential expression.

### RNA interference and plasmid transfection

Two short interfering RNA (siRNA) pairs directed against the NRBP1 sequence (CCTTGAAGATGTCAGGAAT and GTCGAGAAGAGCAGAAGAA) were synthesized, as well as a negative control siRNA (NC). Cloning into the pSliencer4.1-CMV Vector (Ambion) was performed as directed by the manufacturer. Plasmids of NRBP1 full-length and truncations, NRBP1 site-mutations were purchased from *Jiman*, shanghai. Cells underwent transfection with siRNA or plasmids of NRBP1, respectively, utilizing Lipofectamine 3000 (Invitrogen) according the protocol. For TRIM24 rescue experiments in the NRBP1 knockdown model, stable cell lines with TRIM24 knockdown (generated via lentiviral infection) and corresponding control cell lines were transfected with NRBP1 siRNAs. Transfections were conducted for 18 h, after which phenotypic assays, including colony formation, wound healing, and Transwell migration assays, were performed. Transfection efficacy was quantitated by immunoblot.

### Quantitative real-time PCR

Total RNA extraction utilized TRIzol reagent (Invitrogen). Reverse transcription employed the primeScript RT Master Mix (Takara) as instructed by the manufacturer. qPCR employed the iQ SYBR Green Supermix (Bio-Rad) and was carried out on an Eppendorf Mastercycleer ep realplex4 instrument (Eppendorf). Primers (Aikerui Biotech) were: TRIM24, 5′-TCCCCAGTGACCAACAACACC-3′ and 5′-TGTTCCACGACAGGATTCTGC-3′; NRBP1, 5′-GTCTGAGATTTTGGAAGAGTCGC-3′ and 5′- CACAACCTCTACACCTTCCTCTG-3′; GAPDH, 5′-GCATTGCCCTCAACGACCAC-3′ and 5′- CCACCACCCTGTTGCTGTAG-3′. Data analysis applied the 2^-ΔΔCT^ method, with GAPDH for normalization.

### Co-immunoprecipitation

To immune-precipitate endogenous proteins, cell lysates underwent overnight incubation with primary antibodies or control IgG (4 °C). Then, samples underwent a 2-h incubation with protein A/G magnetic beads (Bimake). Immunoblot was performed to assess the obtained immunoprecipitates.

### Clonogenic, migration, invasion and wound healing assays

For clonogenic assay, about 800 MKN74 and NUGC-3 GC cells/well were added to 6-well plates, respectively. Following a 15-day culture with RPMI 1640, fixation and crystal violet (Solarbio, China) staining were carried out.

Migration and invasion assays used transwell plates without and with Matrigel (Corning), respectively. In brief, 5 × 10^4^ cells in FBS-free medium were plated in the superior compartment. Meanwhile, medium with 10% FBS was placed in the inferior compartment. Upon incubation, migratory and invading cells underwent fixation and 0.1% crystal violet staining. A microscope (Axio observer A1, Zeiss, Germany) was employed for data analysis.

For the wound healing assay, about 10^6^ cells/well were added to 6-well plates. At confluency, the culture inserts were removed. After 2 PBS washes, an optical microscope (ix71, Olympus, Japan) was employed for imaging at 0, 24 and 48 h following wounding. The relative migration rate (%) is calculated as following: (0 h scratch width − 48 h scratch width)/0 h scratch width × 100%.

### Cell apoptosis assays

Approximately 3 × 10^5^ cells/well were added to 6-well plates and cultured overnight. Apoptosis was examined flow-cytometrically with the Annexin V-FITC/PI Staining Kit (LiankeBio, Hangzhou) as directed by the manufacturer.

### Orthotopic and liver metastasis of GC tumors in the nude mouse model

Four-week-old male BALB/c nude mice from the experimental animal ministry of Zhejiang Chinese Medical University were used. Each group contained 4–6 mice per model to ensure experimental reliability and adherence to the 3Rs principle for animal welfare. The investigator was blinded to group allocation during the experiment and outcome assessment. The animal study had approval from Animal Ethical and Welfare Committee of Zhejiang Chinese Medical University, following the AAALAC and IACUC guidelines. The animal research in the study was conducted using randomization, concealed allocation, and blinded outcome assessment.

The animals were submitted to a 1-week adaptative housing prior to any treatment. Then, 5 × 10^6^ cells (wild-type GC cells and sorted aggressive-GC cells, or stable knockdown TRIM24 and empty vector-transformed GC cells) were administered by subcutaneous injection for the establishment of a xenograft tumor model. After the longest tumor diameter reached 1 cm, tumors were extracted and minced into 1-mm^3^ fragments. Next, each fragment was placed on the surface of the serosa on the greater curvature of the stomach. Body weights and tumor sizes were measured. Each animal received fluorescein substrate (150 mg/kg) by intraperitoneal injection for live imaging on a Xenogen IVIS 200 imaging system (Caliper Life Sciences, USA) at 7-day intervals. The tumor inhibition rate was analyzed with LT Living Image 4.3.

The liver metastasis model of GC was established as previously described [24]. Briefly, BALB/c nude mice were used for the study. Stable TRIM24-overexpressing and control GC cell lines (MKN74) were prepared, and 2 million cells were slowly injected into the spleen of each mouse. Ten minutes post-injection, the splenic blood vessels and splenogastric artery were ligated, and the spleen was surgically removed. 35 days later, liver tissues were harvested for imaging and enumeration of metastatic nodules.

### Statistical analysis

Statistical analyses were conducted using SPSS 17.0 and GraphPad Prism 9. Unpaired T-tests were used to compare data between two groups. Chi-square tests and Fisher’s exact test were applied for analyzing different parameters. The correlation between two variables was assessed using Pearson’s correlation analysis. Data are expressed as mean ± SD, with all experiments repeated at least three times. P < 0.05 was deemed statistically significant. P-value significance codes: *, p < 0.05; **, p < 0.01; ***, p < 0.001.

### Ethics approval and consent to participate

All experiments were approved by Animal Ethical and Welfare Committee of Zhejiang Chinese Medical University, following the AAALAC and IACUC guidelines (2023-0424-04).

## Results

### TRIM24 is upregulated in highly aggressive GC cells

**Fig. 1:**
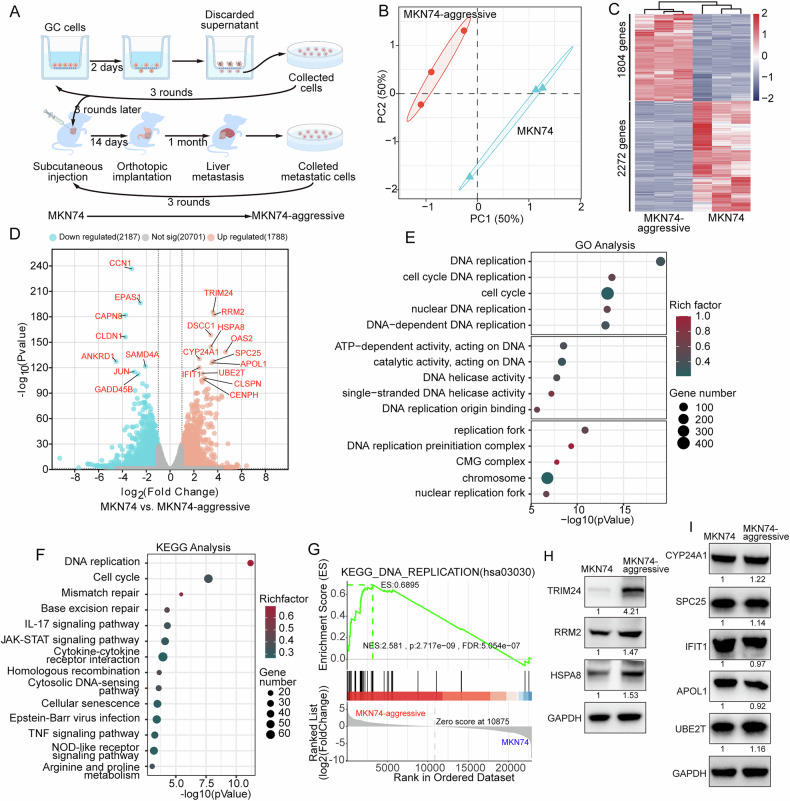
Highly aggressive GC cells exhibit elevated levels of TRIM24 expression. **A** The strategy for screening highly aggressive GC cells (MKN74-aggressive) using Transwell assays and animal models. **B** Quality control of transcriptomic data comparing MKN74-aggressive and MKN74 cells. **C**, **D** Heatmap and volcano plot of differentially expressed genes comparing MKN74-aggressive to MKN74 cells. **E**, **F** GO and KEGG analyses of differentially expressed genes comparing MKN74-aggressive and MKN74 cells. **G** KEGG pathway analysis of DNA replication from GSEA comparing MKN74-aggressive to MKN74 cells. **H**, **I** The protein expressions of TRIM24, RRM2, HSP8, CYP24A1, SPC25, IFIT1, APOL1, and UBE2T were evaluated in MKN74 and MKN74-aggressive cells by WB. The numbers shown below the bands represent the relative densitometry values.

To identify the characteristics of highly aggressive gastric cancer cells, we utilized a combination of in vitro Transwell assays and an in vivo gastric cancer liver metastasis model to screen for highly invasive MKN74-aggressive cells, followed by RNA sequencing (Fig. 1A). The selected MKN74-aggressive cells were further subjected to in vitro and in vivo experiments. Transwell assay results demonstrated that MKN74-aggressive cells exhibited significantly enhanced migration and invasion capabilities (Supplementary Fig. 1A, B). Additionally, in an orthotopic tumor-bearing model, MKN74-aggressive cells showed a stronger tumor growth trend (Supplementary Fig. 1C–F) and a marked propensity for peritoneal and liver metastasis (Supplementary Fig. 1G, H). RNA sequencing analysis revealed that 1804 genes were upregulated and 2274 genes were downregulated in MKN74-aggressive cells compared to the parental MKN74 cells (Fig. 1B, C). Among the top 20 genes with the most significant expression changes, 12 were upregulated (TRIM24, RRM2, DSSC1, HSPA8, OAS2, CYP24A1, SPC25, APOL1, IFIT1, UBE2T, CLSPN, and CENPH) and 8 were downregulated (CCN1, EPAS1, CAPN8, CLDN1, ANKRD1, SAMD4A, JUN, and GADD45B) (Fig. 1D). Furthermore, GO analysis indicated that the differentially expressed genes between MKN74-aggressive and MKN74 cells were associated with pathways related to DNA replication and cell cycle regulation (Fig. 1E, G). KEGG pathway analysis suggested that, in addition to DNA replication and cell cycle pathways, other pathways such as IL-17 signaling, JAK-STAT signaling, TNF signaling, and NOD-like receptor signaling were also implicated (Fig. 1F). To identify key differential proteins, we performed Western blotting to validate the protein expression levels of eight genes that were significantly upregulated in the transcriptome and have been previously associated with tumor progression. The results showed that TRIM24 was significantly upregulated in MKN74-aggressive cells, whereas the increases in RRM2 and HSPA8 were comparatively modest. (Fig. 1H). In contrast, APOL1, CYP24A1, SPC25, IFIT1, and UBE2T did not exhibit notable changes (Fig. 1I). These findings suggest that the elevated expression of TRIM24 in gastric cancer cells may be associated with their increased invasiveness.

### TRIM24 downregulation in GC predicts a better patient prognosis

**Fig. 2:**
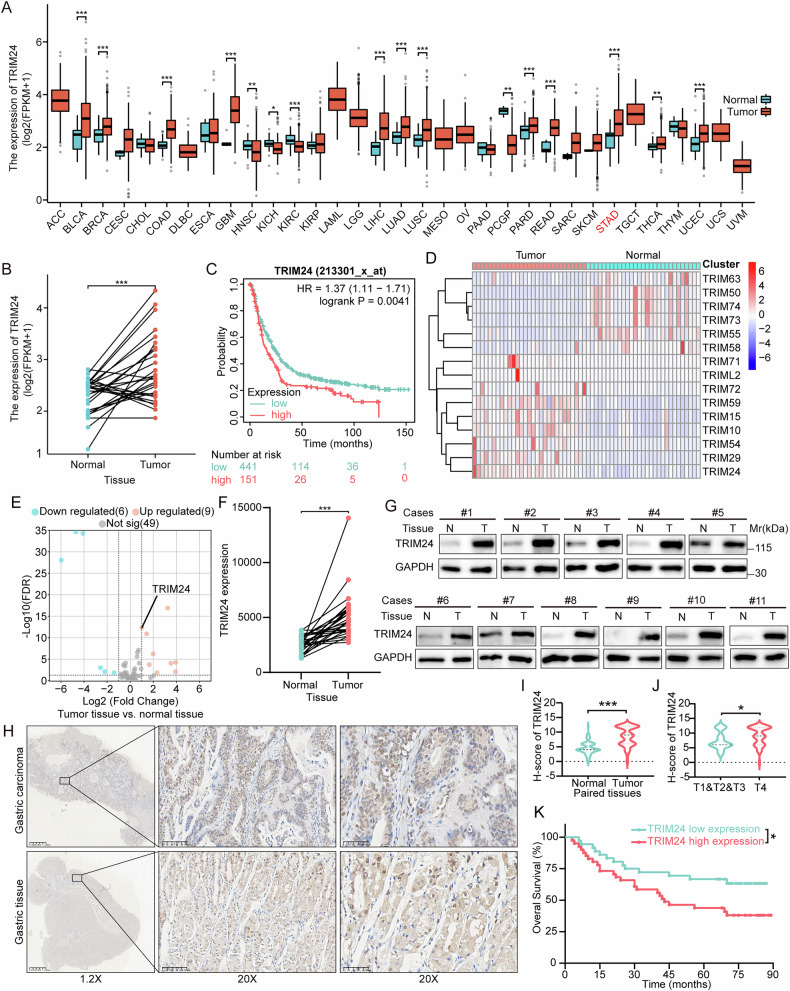
TRIM24 is overexpressed in GC patients, with higher TRIM24 expression predicting poorer prognosis. **A** TRIM24 expression in pan-cancer was analyzed in TCGA and Genotype-Tissue Expression (GTEx). **B** Expression levels of TRIM24 in gastric carcinoma and paired adjacent tissues in TCGA. **C** Kaplan-Meier curve estimates of survival distributions in GC according to TRIM24 expression in the Kaplan-Meier plotter database. **D**, **E** Heat map and volcano map of relative expression levels of differentially expressed TRIM family mRNAs in 29 GC tissues and paired adjacent tissues. **F** TRIIM24 mRNA expression in 29 GC tissues and paired adjacent tissues. **G** TRIM24 protein expression was evaluated in 11 paired GC tissues by WB. **H** Representative IHC staining images of TRIM24 expression in GC and paired adjacent tissues. **I** A statistical chart showing differences in TRIM24 IHC scores between GC tissues and paired adjacent tissues. **J** A statistical chart showing differences in TRIM24 IHC scores between difference T stage of GC tissues. **K** Survival analysis of patients with high and low expression levels of TRIM24 (log-rank test, P < 0.05). Adrenocortical carcinoma (ACC), Bladder Urothelial Carcinoma (BLCA), Breast invasive carcinoma (BRCA), Cervical squamous cell carcinoma and endocervical adenocarcinoma (CESC), Cholangiocarcinoma (CHOL), Colon adenocarcinoma (COAD), Lymphoid Neoplasm Diffuse Large B-cell Lymphoma (DLBC), Esophageal carcinoma (ESCA), Glioblastoma multiforme (GBM), Head and Neck squamous cell carcinoma (HNSC), Kidney Chromophobe (KICH), Kidney renal clear cell carcinoma (KIRC), Kidney renal papillary cell carcinoma (KIRP), Acute Myeloid Leukemia (LAML), Brain Lower Grade. Glioma (LGG), Liver hepatocellular carcinoma (LIHC), Lung adenocarcinoma (LUAD), Lung squamous cell carcinoma (LUSC), Mesothelioma (MESO), Ovarian serous cystadenocarcinoma (OV), Pancreatic adenocarcinoma (PAAD), Pheochromocytoma and Paraganglioma (PCPG), Prostate adenocarcinoma (PRAD), Rectum adenocarcinoma (READ), Sarcoma (SARC), Skin Cutaneous Melanoma (SKCM), Stomach adenocarcinoma (STAD), Testicular Germ Cell Tumors (TGCT), Thyroid carcinoma (THCA), Thymoma (THYM), Uterine Corpus Endometrial Carcinoma (UCEC), Uterine Carcinosarcoma (UCS), Uveal Melanoma (UVM). *, p < 0.05; **, p < 0.01; ***, p < 0.001.

To determine TRIM24 expression in cancers, we firstly evaluated TRIM24 levels in TCGA pan-cancer (33 cancer types), with GTEx employed to obtain TRIM24 mRNA amounts based on the XIANTAO online tool. TRIM24 upregulation was detected in 16/33 cancer types, including GC (Fig. 2A). Furthermore, TRIM24 levels were elevated in most GC tissue samples in comparison with paired noncancerous gastric mucosal tissue samples in public datasets from TCGA (Fig. 2B). Employing the online Kaplan–Meier plotter database, we determined TRIM24 overexpression in GC patients predicted poor overall survival (OS) (Fig. 2C). After that, 29 gastric cancer patients at Zhejiang Cancer Hospital had their tumor tissues and normal mucosal tissues sequenced and analyzed for transcriptomic analysis. TRIM family proteins were then selected for analysis. In the samples, 80 TRIM family genes were detected, among which the mRNA of 9 TRIM proteins (including TRIM24) was up-regulated, and the mRNA of 6 TRIM proteins was down-regulated (Fig. 2D–F and Supplementary Fig. 2A). At least at the transcriptional level, these results suggest that the TRIM24 mRNA is markedly overexpressed in GC tissues compared with paired non-cancerous tissues.

To further elucidate the significance of TRIM24 protein expression in GC, TRIM24 levels were assessed in 11 pairs of fresh samples collected from Zhejiang Cancer Hospital, revealing that GC tissue samples had higher TRIM24 protein levels versus paired adjacent noncancerous tissue samples (Fig. 2G). Moreover, we collected frozen sections of GC and paired cancer from the First Affiliated Hospital of Zhejiang Chinese Medicine University. IHC staining further confirmed this finding (Fig. 2H). Subsequently, TRIM24 expression IHC scores were assigned according to positive staining area percentages and intensity scores (Supplementary Fig. 2B). As depicted in Fig. 2I and Supplementary Fig. 2C, TRIM24 expression was always enhanced in GC tissue samples in comparison with adjacent tissue samples, whether in paired or unmatched samples. In addition, TRIM24 expression was associated with tumor T stage, indicating that TRIM24 predicted the depth of tumor invasion (Fig. 2J). Moreover, higher TRIM24 expression was associated with lower CD3 expression, demonstrating that TRIM24 inhibited the recruitment of immune cells (Supplementary Fig. 2D). According to median expression score in carcinoma, the samples were categorized as TRIM24-high or TRIM24-low. Interestingly, TRIM24 overexpression had significant associations with T stage (P = 0.014) and CD3 expression (P = 0.046), but no associations with age, sex, Lauren type, tumor size, Tumor location, Differentiated degree, N stage, M stage, TNM stage, PD-L1 expression and CLDN18.2 expression (Table 1). Subsequently, we performed COX regression analysis on variables such as sex, age, tumor location, differentiated degree, TNM stage, and TRIM24 expression, and found that M stage and high TRIM24 expression were both independent adverse prognostic factors for GC patients (Table 2). Kaplan-Meier curves and the logrank test for OS analysis revealed cases with elevated TRIM24 expression had poorer prognosis compared with individuals with reduced TRIM24 amounts (Fig. 2K). These results suggest TRIM24 upregulation in GC, which could predict a poor prognosis.

**Table 1:** Correlation between TRIM24 expression and clinical characteristics.

Variable	TRIM24 expression		Total	*χ*2	*p*-value
	High	Low			
Age					
≤65	24	22	46	0.126	0.722
>65	18	14	32		
Sex					
Female	12	9	21	0.126	0.723
Male	30	27	57		
Lauren type					
Intestinal GC	24	15	39	1.585	0.453
Diffuse GC	12	13	25		
Mixed GC	3	4	7		
Unknown	3	4	7		
Tumor location					
Proximal stomach	14	8	22	2.265	0.322
Distal stomach	25	27	52		
Total stomach	3	1	4		
Differentiated degree					
Moderate	9	3	12	2.702	0.1
Poor	32	33	65		
Unknown	1	0	1		
T stage					
T1 + T2 + T3	9	17	26	6.098	0.014*
T4	31	17	48		
Unknown	2	2	4		
N stage					
N0 + N1	10	7	17	0.202	0.653
N2 + N3	30	27	57		
Unknown	2	2	4		
M stage					
M0	38	34	72	0.363	0.547
M1	2	0	2		
Unknown	2	2	4		
TNM stage					
II	4	7	11	1.628	0.202
III + IV	36	27	63		
Unknown	2	2	4		
CD3 expression					
Low	27	15	42	3.991	0.046*
High	15	21	36		
PD-L1 expression					
Negative	33	31	64	0.748	0.387
Positive	9	5	14		
CLDN18.2 expression					
Negative	19	20	39	1.1	0.294
Positive	19	12	31		
Unknown	4	4	8		

^*^Statistically significant (p < 0.05).

**Table 2:** Cox Proportional Hazards Regression Analysis.

Variable	B	SE	Wald	P value	OR value	95% CI of OR value
Gender (Female* vs. Male)	−0.118	0.406	0.085	0.770	0.888	00.401–1.967
Age (≤65 vs. >65)	0.529	0.359	2.171	0.141	1.697	0.840–3.431
Tumor location (Proximal vs. Distal vs. total)	−0.293	0.372	0.623	0.430	0.746	0.360–1.545
Differentiated degree (Poor vs. Moderate)	−0.976	0.597	2.669	0.102	0.377	0.117–1.215
T (T1-3 vs. T4)	0.140	0.545	0.066	0.798	1.150	0.395–3.345
N (N1 vs. N2-3)	0.607	0.499	1.480	0.224	1.835	0.690–4.883
M (M0 vs. M1)	2.435	0.867	7.892	0.005*	11.412	2.088–62.383
TNM (II vs. III-IV)	−0.917	0.710	1.669	0.196	0.400	0.099–1.607
TRIM24 expression (Low vs. high)	1.061	0.426	6.221	0.013*	2.890	1.255–6.655

*Statistically significant (p < 0.05).

### TRIM24 expression promotes the malignant potential of cultured GC cells

**Fig. 3:**
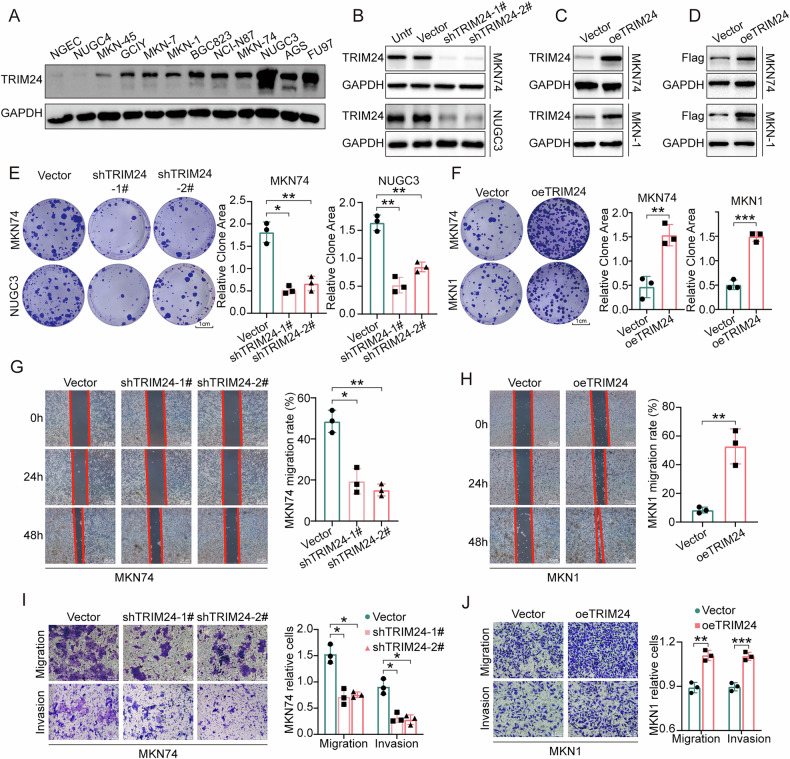
TRIM24 promotes GC cell migration, invasion in vitro. **A** Expression of TRIM24 in normal gastric epithelial cell line NGEC (non-malignant gastric epithelial cell lines derived from healthy gastric tissue) and GC cell lines (NUGC4, MKN45, GCIY, MKN7, MKN1, BGC823, NCI-N87, MKN74, NUGC3, AGS and FU97) determined by western blot. **B**–**D** MKN74, NUGC3 and/or MKN1 were transfected with vector lentivirus, TRIM24 shRNA lentiviruses (1# and 2#), and/or TRIM24-overexpressing lentiviruses and verified by WB. Colony forming (**E**, **F**), wound healing (**G**, **H**), and migration and invasion (**I**, **J**) were performed in MKN74 and MKN1 cells transfected with vector lentivirus, TRIM24 shRNA lentiviruses (1# and 2#), and/or TRIM24-overexpressing lentiviruses, respectively. Data represent mean ± SD (n = 3). *P < 0.05, **P < 0.01, ***P < 0.001.

To investigate TRIM24’s function, TRIM24 expression was determined in 11 gastric cancer and 1 normal gastric mucosal cell lines by immunoblot. As depicted in Fig. 3A, TRIM24 protein amounts were markedly higher in most GC cells in comparison with noncancerous cells. Short-hairpin RNA (shRNA) TRIM24-knockdown and TRIM24 overexpression lentiviruses were transfected into NUGC3, MKN74, or/and MKN1 cells, and transfection efficiency was confirmed (Fig. 3B–D). The clonogenic assay revealed TRIM24 knockdown markedly decreased clonogenicity, which was enhanced by TRIM24 overexpression in GC cells (Fig. 3E, F). Additionally, the wound healing assay demonstrated TRIM24 silencing resulted in retarded wound closure in the MKN74 and NUGC3 GC cell lines (Fig. 3G and Supplementary Fig. 3A), whereas TRIM24 overexpression resulted in rapid wound closure in MKN74 and MKN1 GC cells (Fig. 3H and Supplementary Fig. 3B). Consistently, transwell assays showed TRIM24 knockdown and overexpression substantially impeded and enhanced the numbers of migratory and invading GC cells, respectively (Fig. 3I, J and Supplementary Fig. 3C, D). Furthermore, we utilized TRIM24 inhibitors (dTRIM24 and IACS-9571), which have been reported in previous studies, to treat GC cells. The results showed that TRIM24 inhibitors not only significantly reduced TRIM24 expression but also markedly suppressed the migration and invasion of GC cells (Supplementary Fig. 3E–G). Taken together, the above data indicated that TRIM24 knockdown decreases the malignant potential of GC cells, while TRIM24 overexpression had the opposite effects.

### Knockdown of TRIM24 represses tumor growth and metastasis in a mouse model

**Fig. 4:**
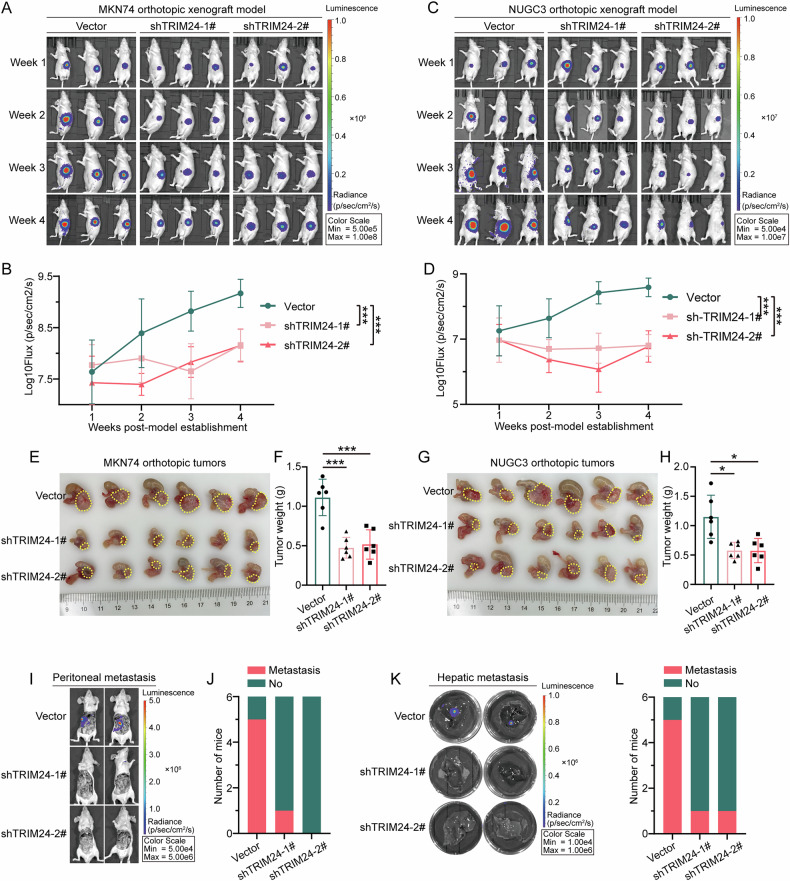
TRIM24 promotes tumor proliferation, invasion and metastasis in vivo. **A**–**D** The fluorescence images (representative) and values of tumor-bearing mice were obtained and detected in the vector and shTRIM24 (#1 and #2) groups using the IVIS imaging system. **E**–**H** The tumor sizes and weights of tumor-bearing mice were determined in the vector and shTRIM24 (#1 and #2) groups. **I**, **J** Representative images of peritoneal metastasis and numbers of mice with peritoneal metastasis in the vector and TRIM24 shRNA lentiviruses (1# and 2#) groups. **K**, **L** Representative images of hepatic metastasis and numbers of mice with hepatic metastasis in the vector and TRIM24 shRNA lentiviruses (1# and 2#) groups. Data represent mean ± SD (n = 6). *P < 0.05, **P < 0.01, ***P < 0.001.

In order to examine TRIM24’s function in GC cells in vivo, an orthotopic tumor mouse model of GC was established using vector-luciferase or knockdown TRIM24-luciferase lentivirus-transfected MKN74 and NUGC3 GC cells. Western blot and IHC staining re-verified the knockdown efficiency of TRIM24 silencing in gastric xenograft tumors (Supplementary Fig. 4A, B). As depicted in Fig. 4A–D and Supplementary Fig. 4C, D, TRIM24 knockdown significantly reduced tumor fluorescence values in MKN74- and NUGC3-orthotopic tumor models. Furthermore, TRIM24 downregulation remarkably reduced MKN74- and NUGC3-orthotopic tumor sizes and tumor weights (Fig. 4E–H). Furthermore, fluorescence imaging of mice without stomach and fluorescence imaging of the liver were carried out to determine TRIM24’s association with peritoneal or liver metastasis of GC in vivo. Compared with the control group, TRIM24 knockdown groups showed reduced intraperitoneal metastasis with decreased intraperitoneal metastasis fluorescence values for both MKN74 and NUGC3 GC cells (Fig. 4I–J). Similarly, the numbers of mice with liver metastasis and fluorescence values for liver metastasis were reduced in the TRIM24 knockdown groups (Fig. 4K, L). Furthermore, the number of liver metastasis nodules is significantly decreased (Supplementary Fig. 4E, F), while TRIM24 knockdown does not affect liver function (Supplementary Fig. 4G, H). Subsequently, CRISPR-Cas9 was employed to knockout TRIM24 in MKN74 cells (Supplementary Fig. 4I). The resulting orthotopic GC model likewise exhibited reduced intragastric fluorescence intensity and tumor mass (Supplementary Fig. 4J, K), accompanied by diminished liver and spleen metastases (Supplementary Fig. 4L, M). To further elucidate the critical role of TRIM24 in GC metastasis, we constructed a liver metastasis model using GC cell lines with control or TRIM24 overexpression. The results showed that TRIM24 overexpression significantly enhanced the liver metastatic capacity of GC cells (Supplementary Fig. 4N, O), and this enhanced metastatic ability was not entirely dependent on the size of the primary tumor.

These data strongly suggested that TRIM24 promotes GC progression and metastasis in vivo.

### Proteomic analysis revealed a negative correlation between TRIM24 and NRBP1

**Fig. 5:**
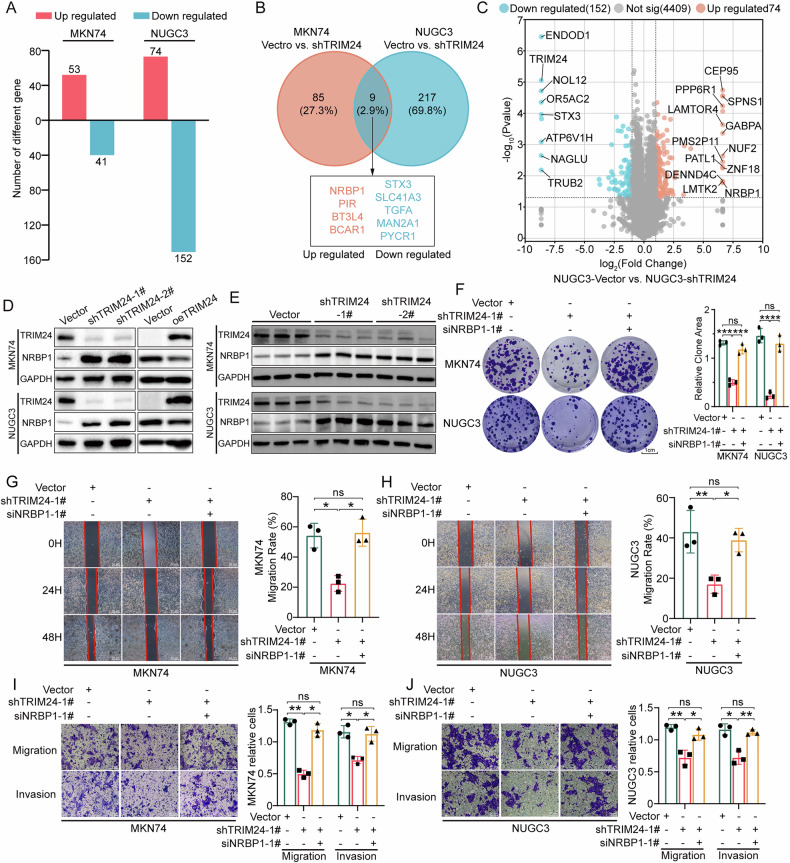
Proteomic analysis of MKN74 and NUGC3 GC cells indicates NRBP1 is the key effector in TRIM24-mediated malignancy of GC. **A** The numbers of distinct proteins found by mass spectrometry in MKN74 and NUGC3 cells transfected with TRIM24 shRNA lentivirus compared with vector lentivirus. **B** Common differentially expressed proteins identified by proteomic screening of MKN74 and NUGC3 cells. **C** Volcano plot showing differentially expressed proteins in NUGC3 cells following TRIM24 knockdown. **D** TRIM24 and NRBP1 protein amounts evaluated in the vector, TRIM24 shRNA (1# and 2#), and overexpression TRIM24 lentiviruses groups by WB. **E** TRIM24 and NRBP1 protein amounts evaluated in orthotopic tumors of MKN74 and NUGC3 transfected vector, TRIM24 shRNA (1# and 2#), and overexpression TRIM24 lentivirus by WB. Colony forming (**F**), wound healing (**G**, **H**), migration and invasion (**I**, **J**) assays were performed in MKN74 and NUGC3 cells transfected with TRIM24 shRNA-1# lentivirus alone or TRIM24 shRNA-1# lentivirus and NRBP1 siRNA-1# simultaneously. Data represent mean ± SD (n = 3). Data are shown as mean ± SD. ns no statistical difference, *P < 0.05, **P < 0.01, ***P < 0.001.

To examine how TRIM24 induces GC progression, quantitative mass spectrometry (MS) of the proteome was performed to analyze differences between MKN74/NUGC3 GC cells and TRIM24 knockdown MKN74/NUGC3 GC cells. Totally 5718 proteins were annotated. After preliminary screening, 94 and 226 proteins showed differential expression in MKN74 and NUGC3 cells, respectively (Fig. 5A). In comparison with respective control groups, 53 and 74 proteins were upregulated while 41 and 152 were downregulated in TRIM24-knockdown MKN74 and NUGC3 groups, respectively. Additionally, there were 4 common differentially upregulated (NRBP1, PIR, BT3L4 and BCAR1) and 5 common differentially downregulated (STX3, SLC41A3, TGFA, MAN2A1 and PYCR1) proteins in 2 GC cell lines with knockdown of TRIM24 (Fig. 5B, C and Supplementary Fig. 5A). We used TRIM24 knockdown MKN74 cells to validate the above results. We found that knockdown of TRIM24 led to a potential upregulation of NRBP1, while BCAR1, BTF3L4, and PIR showed only slight increases (Supplementary Fig. 5B). Additionally, both MAN2A1 and TGFA3 exhibited slight decreases, whereas STX3, SLC41A3 and PYCR1 showed no significant changes (Supplementary Fig. 5B). Given that TRIM24 is recognized as a ubiquitin E3 ligase that promotes the ubiquitination of numerous substrates, including some tumor suppressors, thereby exerting its oncogenic effects, our study focused on the proteins whose expression increased after TRIM24 knockdown. Previous studies have suggested that genes such as BCAR1 [25] and PIR [26] in tumor cells are considered oncogenic factors, with their increased expression in tumor tissues being closely associated with poor prognosis. However, there is limited research on BTF3L4 in cancer. Given its slight increase in expression following TRIM24 knockdown, we excluded these genes from further investigation in our study.

Interestingly, NRBP1 (nuclear receptor binding protein 1) exerts distinct roles in different tumor contexts. For instance, in lung and breast cancers NRBP1 has been identified as a potential tumor suppressor [27–29], whereas in prostate cancer it has been implicated in tumor-promoting functions [30]. Based on these observations, we further investigated whether TRIM24 regulates NRBP1 and how NRBP1 functions specifically in GC. Our results demonstrated that NRBP1 expression was enhanced in knockdown TRIM24 cell lines and mouse xenografts (Fig. 5D, E). The results were consistent with proteomics findings.

To determine whether TRIM24 promotes GC proliferation, invasion and metastasis by abrogating NRBP1-mediated tumor suppression, siRNA targeting NRBP1 was employed to transfect stable TRIM24 knockdown MKN74 and NUGC3 cells. Firstly, two NRBP1-specific siRNAs successfully suppressed the upregulation of NRBP1 induced by TRIM24 knockdown (Supplementary Fig. 5C, D). Consistent with the above results, TRIM24 knockdown reduced clonogenicity and migratory and invasive properties in MKN74 and NUGC3 GC cells. However, NRBP1 knockdown using siRNA-1# effectively relieved these effects (Fig. 5F–J and Supplementary Fig. 5E–I). Similarly, siRNA-2# also rescued the proliferative and migratory abilities of NUGC3 cells (Supplementary Fig. 5J, K). Overall, the above data suggested NRBP1 controlled TRIM24-regulated GC development.

### Knockdown of TRIM24 promotes apoptosis in GC cells by mediating NRBP1 expression

**Fig. 6:**
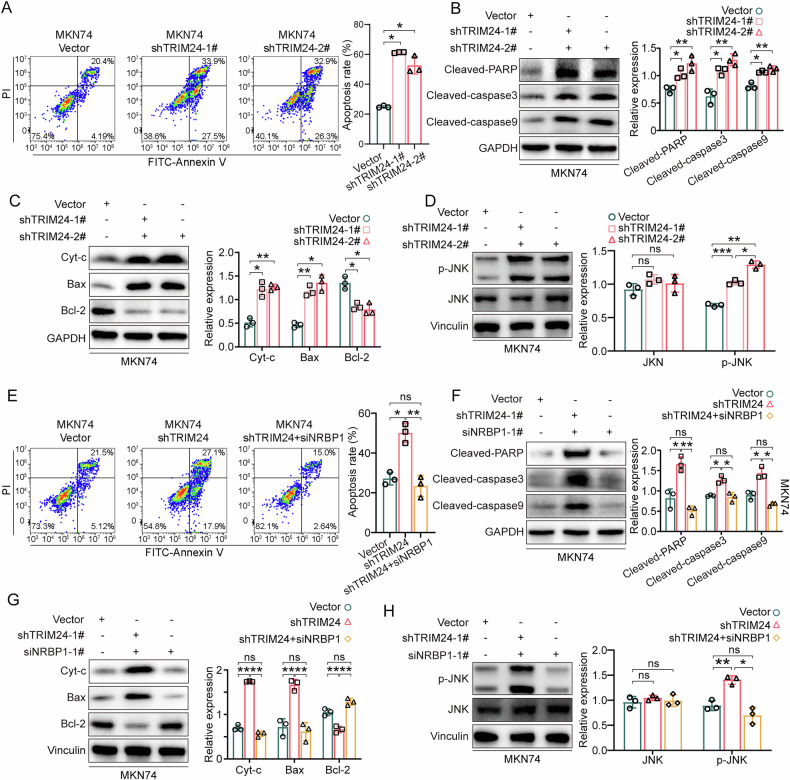
Knockdown of TRIM24 induces apoptosis by increasing NRBP1 expression in GC cells. **A** Apoptotic cell analysis by flow cytometry after transfection with TRIM24 shRNA (1# and 2#) lentivirus in MKN74. Annexin V-FITC and PI were used to assess apoptotic events. **B**, **C** Protein expression levels of the intrinsic apoptosis markers cleaved PARP, cleaved caspase 3, cleaved caspase 9, cytosolic cytochrome c (Cyt-c), Bax, and Bcl-2 in MKN74 and after TRIM24 knockdown. **D** Protein expression levels of JNK and p-JNK in MKN74 after TRIM24 knockdown. **E** Apoptotic cell analysis by flow cytometry after transfection with TRIM24 shRNA lentivirus alone or simultaneously transfected with TRIM24 shRNA lentivirus and NRBP1 siRNA-1# in MKN74. Annexin V-FITC and PI were used to assess apoptotic events. **F**, **G** Protein expression levels of the intrinsic apoptosis markers clv-PARP, clv-caspase 3, clv-caspase 9, Cyt-c, Bax, and Bcl-2 in MKN74 after transfection with TRIM24 shRNA lentivirus alone or simultaneously transfected with TRIM24 shRNA lentivirus and NRBP1 siRNA-1#. **H** Protein expression levels of JNK and p-JNK in MKN74 after transfection with TRIM24 shRNA lentivirus alone or simultaneously transfected with TRIM24 shRNA lentivirus and NRBP1 siRNA-1#. Data are shown as mean ± SD. ns: no statistical difference, *P < 0.05, **P < 0.01, ***P < 0.001.

Previous studies showed that NRBP1 enhances caspase-dependent intrinsic apoptosis via JNK signaling in colorectal cancer [31, 32]. As depicted in Fig. 6A and Supplementary Fig. 6A, TRIM24 knockdown markedly increased apoptotic rates in MKN74 and NUGC3 GC cells. The caspase cascade plays a crucial role in executing apoptosis. Caspase 9 is indispensable for the intrinsic apoptotic pathway, while caspase 3 drives apoptosis. Our data demonstrated that TRIM24 silencing elevated cleaved caspase 3 and 9 amounts (Fig. 6B and Supplementary Fig. 6B). The pro-apoptotic member Bax promotes mitochondrial outer membrane permeabilization, whereas the anti-apoptotic protein Bcl-2 counteracts this process by preserving mitochondrial integrity. The balance between Bax and Bcl-2 determines cytochrome c (Cyt-c) release from mitochondria into the cytoplasm, thereby regulating caspase activation [33]. As depicted in Fig. 6C and Supplementary Fig. 6C, TRIM24 knockdown significantly increased Cyt-c levels and resulted in upregulated Bax and downregulated Bcl-2 in MKN74 and NUGC3 cells. A knockdown of TRIM24 also effectively increased p-JNK (Tyr185) expression, but not JNK itself (Fig. 6D and Supplementary Fig. 6D). The above findings indicate that TRIM24 knockdown induces GC cell apoptosis via the intrinsic pathway with the activation of the p-JNK and activated caspase cascade.

NRBP1 silencing was used to verify NRBP1’s function in promoting MKN74 and NUGC3 cell apoptosis by TRIM24 knockdown. SiRNA-mediated NRBP1 knockdown successfully rescued the increase in apoptosis induced by TRIM24 knockdown (Fig. 6E and Supplementary Fig. 6E). Furthermore, siRNA-mediated NRBP1 knockdown greatly reduced the upregulation of cleaved PARP, cleaved caspase 3, cleaved caspase 9, Cyt-c, and Bax induced by TRIM24 knockdown, while inhibiting Bcl-2 downregulation (Fig. 6F, G and Supplementary Fig. 6F, G). In addition, p-JNK expression successfully rescued by NRBP1 siRNA (Fig. 6H and Supplementary Fig. 6H). These above data indicate TRIM24 knockdown promotes apoptosis in GC cells by mediating NRBP1 expression.

### TRIM24 binds to NRBP1 and induces its ubiquitination

**Fig. 7:**
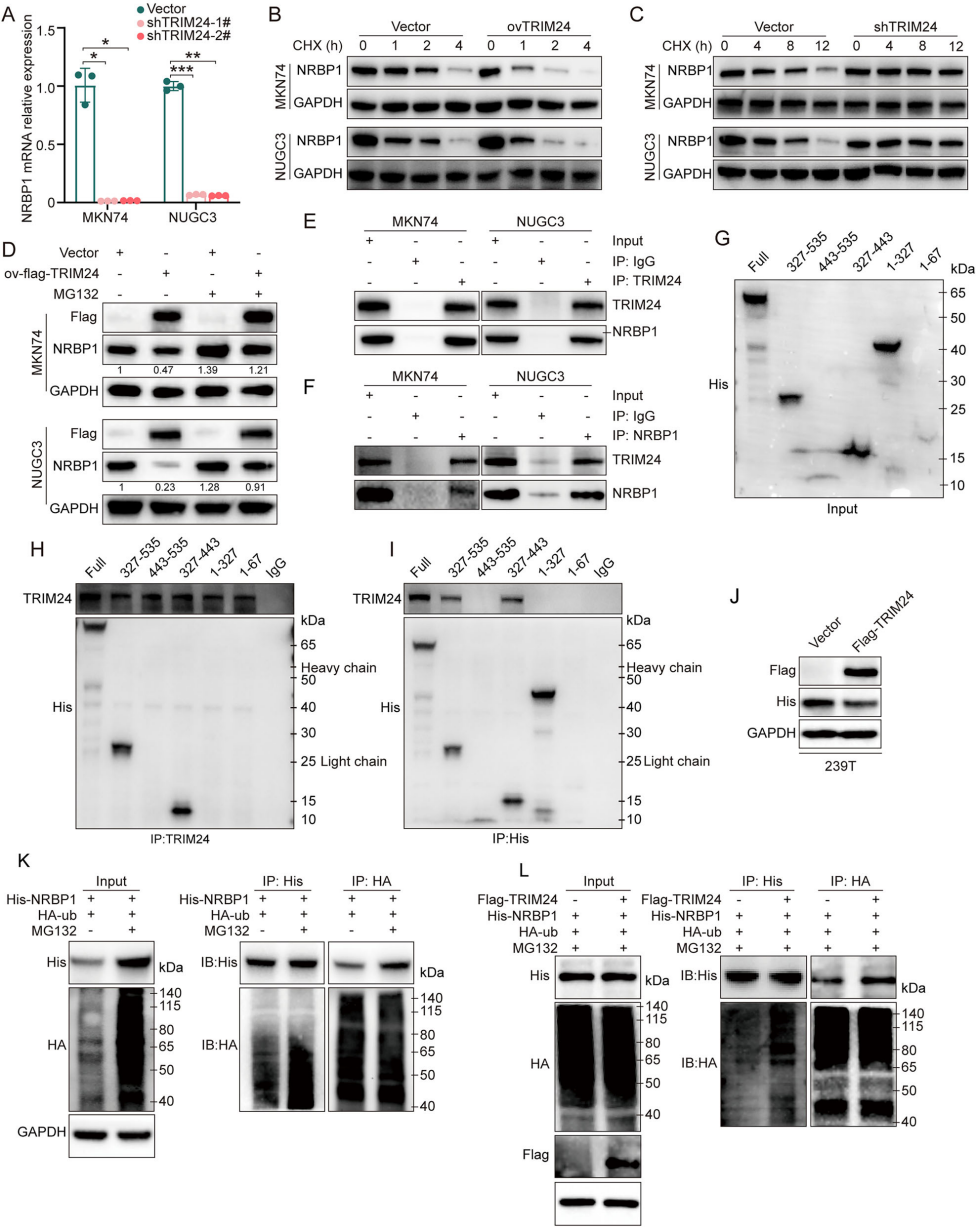
TRIM24 interacts with NRBP1 and promotes its polyubiquitination. **A** NRBP1 mRNA expression in the vector and shTRIM24 (1# and 2#) groups detected by qPCR. **B**, **C** GC cells infected with vector, overexpression TRIM24, and TRIM24 shRNA lentiviruses were treated with 100 µg/ml cycloheximide, and protein lysates were collected at the indicated times for WB. **D** Vector and overexpression TRIM24 GC cells were treated with 10 µg/mL MG-132 for 6 h and analyzed by WB. The numbers shown below the bands represent the relative densitometry values. **E** MKN74 and NUGC3 cell lysates were immunoprecipitated with control IgG or anti-TRIM24 antibody, followed by WB. **F** MKN74 and NUGC3 cell lysates were immunoprecipitated with control IgG or anti-NRBP1 antibody, followed by WB. **G**–**I** Transfect His-NRBP1 truncation fragments into HEK293T cells, followed by immunoprecipitation and WB analysis. **J** HEK293T cells (vector or overexpression flag-TRIM24) transfected with His-NRBP1 plasmid, and followed by WB. **K** HEK293T cells expressing His-NRBP1 and HA-ub were treated with 10 µg/mL MG-132 for 6 h and subjected to sequential immunoprecipitation and WB. **L** HEK293T cells expressing His-NRBP1, HA-ub, and Flag-TRIM24 were treated with 10 µg/mL MG-132 for 6 h and subjected to sequential immunoprecipitation-WB. Data represent mean ± SD (n = 3). *P < 0.05, **P < 0.01, ***P < 0.001.

To examine how TRIM24 controls NRBP1 expression, NRBP1 protein levels were quantitated after TRIM24 knockdown. As depicted in Fig. 7A, TRIM24 knockdown reduced NRBP1 mRNA levels. Moreover, TRIM24 overexpression significantly enhanced the degradation of NRBP1 with cycloheximide (CHX) treatment in GC cells, while TRIM24 knockdown delayed NRBP1 degradation in cells (Fig. 7B, C and Supplementary Fig. 7A, B). This may be because TRIM24 affects the protein stability of NRBP1. Specifically, TRIM24 knockdown may improve the stability of the NRBP1 protein and subsequently negatively inhibit NRBP1 mRNA levels.

We hypothesized that NRBP1 might be targeted for degradation by the ubiquitin-specific protease (USP) system, given that TRIM24 possesses E3 ubiquitin ligase activity [34, 35]. Consistent with this hypothesis, the proteasome inhibitor MG132 markedly attenuated the NRBP1 downregulation caused by overexpressed TRIM24 (Fig. 7D). Subsequently, coimmunoprecipitation (co-IP) assays demonstrated that interaction between TRIM24 and NRBP1 occurred with endogenous protein levels in MKN74 and NUGC3 cells (Fig. 7E, F). To further define the TRIM24-binding site in NRBP1, truncated NRBP1 plasmids were designed according to the possible domain predicting by *InterPro* (http://www.ebi.ac.uk/interpro/), and transfected in HEK293T cells (Supplementary Fig. 7C). The results indicated that the NRBP1(1-67) truncation exhibited instability with the significantly low expression (Supplementary Fig. 7D). Among these findings, the observed band shift of the 1-67 truncation might result from stabilizing modifications or non-specific detection by the antibody. Furthermore, co-IP assays demonstrated that neither of the NRBP1 truncations, NRBP1(1-67) nor NRBP1(1-327), could interact with TRIM24, while a clear interaction between full-length NRBP1 and TRIM24 was shown (Supplementary Fig. 7E). These results suggest that TRIM24 may interact with the 327-535 region of NRBP1. To further investigate this, we constructed NRBP1(327-443) and NRBP1(443-535) plasmids (Supplementary Fig. 7F). Co-IP results revealed that the NRBP1(327-443) segment is the primary domain responsible for interacting with TRIM24 (Fig. 7G–I).

To determine whether TRIM24 promotes NRBP1 protein ubiquitination, co-transfection was firstly carried out with His-NRBP1, HA-ubiquitin (ub), or/and Flag-TRIM24 in HEK293T cells (Fig. 7J). Co-IP revealed significantly enhanced interaction between His-NRBP1 and HA-ub after MG132 treatment (Fig. 7K). Furthermore, TRIM24 overexpression (Flag-TRIM24) significantly increased the interaction of His-NRBP1 with HA-ub (Fig. 7L). Jointly, these data indicate TRIM24 regulates NRBP1 protein degradation by controlling NRBP1 ubiquitination.

### TRIM24 promotes ubiquitination of S42 phosphorylated NRBP1 at K430

**Fig. 8:**
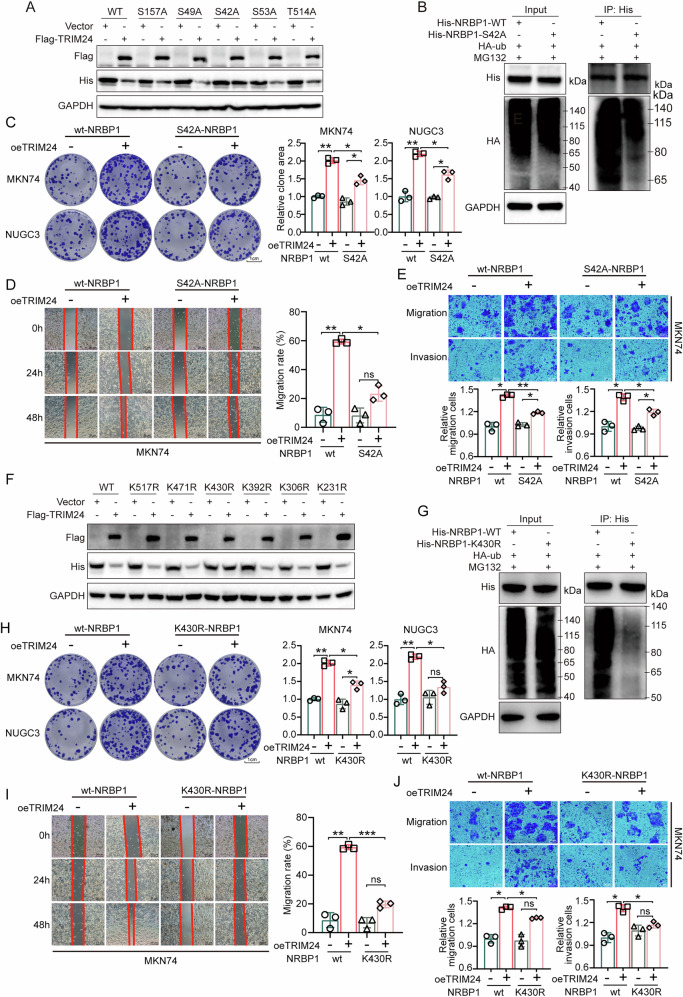
TRIM24 promotes ubiquitination of S42 phosphorylated NRBP1 at the K430 residue. **A** Effects of the mutations of five phosphorylation sites on TRIM24-mediated NRBP1 expression in HEK293T cells, detected by WB. **B** Effects of NRBP1 S42 site mutation on TRIM24-mediated NRBP1 ubiquitination in HEK293T cells. Colony forming (**C**), wound healing (**D**), migration and invasion (**E**) assays were performed in MKN74 (vector or overexpression TRIM24) transfected with NRBP1 S42 site mutation plasmid. **F** Effects of the mutations of six ubiquitination sites on TRIM24-mediated NRBP1 expression in HEK293T cells, detected by WB. **G** Effect of NRBP1 K430 site mutation on TRIM24-mediated NRBP1 ubiquitination in HEK293T cells. Colony forming (**H**), wound healing (**I**), migration and invasion (**J**) assays were performed in MKN74 (vector or overexpression TRIM24) transfected with NRBP1 K430 site mutation plasmid. Data are shown as mean ± SD. ns no statistical difference, *P < 0.05, **P < 0.01, ***P < 0.001.

Several proteins require phosphorylation to exert their biological activities. To investigate whether the phosphorylation of NRBP1 affects its ubiquitination-degradation by TRIM24, post-translational modification sites on NRBP1 were examined, and five phosphorylation sites, namely S517, S49, S42, S53 and T514, were identified utilizing information from the PhosphoSitePlus database (www.phosphosite.org) (Supplementary Fig. 8A). Subsequently, these putative phosphorylation sites were mutated to Alanine and cloned into plasmids (S517A, S49A, S42A, S53A, and T514A) (Supplementary Fig. 8B). The resulting constructs were then separately transfected into HEK293T cells expressing either control or overexpressed TRIM24 (Flag-TRIM24). The data revealed that overexpression of TRIM24 successfully degraded wild-type (wt) NRBP1 as well as the phosphorylation mutants at four sites, except for NRBP1 with the S42 site mutation (S42A-NRBP1) (Fig. 8A). Moreover, co-IP confirmed a significant decrease in the interaction between S42A-NRBP1 and ubiquitin (Fig. 8B). To further verify whether the phosphorylation of NRBP1 at S42 is the key to TRIM24’s ability to promote GC growth, clone formation, wound-healing, and transwell assays were conducted. Our experiments indicate that TRIM24 markedly enhanced biological endpoints in the presence of WT NRBP1, whereas these effects were attenuated and much less pronounced in the presence of the S42A mutant (Fig. 8C–E and Supplementary Fig. 8C, D). Subsequently, we identified Casein Kinase 1 (CK1) as a potential kinase responsible for the phosphorylation at the S42 site of NRBP1 in the online database (Table 3). The results suggest that treatment of HEK293T cells with a CK1 inhibitor suppressed the reduction of NRBP1 protein and weakened the interaction between TRIM24 and NRBP1 (Supplementary Fig. 8E). These findings suggest that phosphorylation at the S42 site of NRBP1 may be a critical prerequisite for TRIM24-mediated degradation.

**Table 3:** Predicted Phosphorylation sites and corresponding kinases of NRBP1 based on PhosphoSitePlus database.

Score	Percentile	Motif	Motif Group	Site	Sequence	Surface Accessibility	Gene Info	Previously Mapped Site	Evolutionary conservation	Colocalization
0.318	0.00062	Casein Kinase 1 (Casn_Kin1)	Acidophilic serine/threonine kinase group (Acid_ST_kin)	S157	TEYMSSGsLKQFLKK	1.1633	CSNK1G2 KC1G2_HUMAN	NA	Scan orthologs	cytoplasm
0.354	0.00194	Casein Kinase 1 (Casn_Kin1)	Acidophilic serine/threonine kinase group (Acid_ST_kin)	S42	TSTTSAAsPEEEEES	1.4536	CSNK1G2 KC1G2_HUMAN	NA	Scan orthologs	cytoplasm
0.227	0.00012	Casein Kinase 2 (Casn_Kin2)	Acidophilic serine/threonine kinase group (Acid_ST_kin)	S49	SPEEEEEsEDESEIL	4.6135	CSNK2B CSK2B_HUMAN	PhosphoSitePlus (2015/02/09)	Scan orthologs	nucleus
0.368	0.00156	Casein Kinase 2 (Casn_Kin2)	Acidophilic serine/threonine kinase group (Acid_ST_kin)	S53	EEESEDEsEILEESP	0.8892	CSNK2B CSK2B_HUMAN	NA	Scan orthologs	nucleus
0.539	0.00108	Nek7 (Nek7)	Phosphoserine/threonine binding group (pST_bind)	T514	LTSLLEEtLNKFNFA	2.732	NEK7	NA	Scan orthologs	unknown/NA

*NA* not apply.

To determine the site of NRBP1 ubiquitination, post-translational modification sites on NRBP1 were assessed (www.phosphosite.org), and 6 ubiquitination sites were detected, i.e., K517, K471, K430, K392, K306, and K231 (Supplementary Fig. 8F). Subsequently, employing similar methodologies, the putative ubiquitylation sites were mutated to arginine and cloned into plasmids (K517R, K471R, K430R, K392R, K306R, and K231R) (Supplementary Fig. 8G). The results indicated that TRIM24 overexpression effectively degraded both wt-NRBP1 and its mutants at the five identified sites, except for the NRBP1 mutant with the K430 site mutation (K430R-NRBP1) (Fig. 8F). Further co-IP confirmed a significant reduction in the interaction of K430R-NRBP1 and ubiquitin molecules (Fig. 8G). Moreover, clone formation, wound-healing, and transwell assays confirmed that TRIM24 overexpression couldn’t reduce the anti-tumor effect of K430R-NRBP1 (Fig. 8H–J and Supplementary Fig. 8H, I). These findings suggest that TRIM24 facilitates NRBP1 degradation by inducing ubiquitination modification at the K430 site.

## Discussion

PTMs of proteins play critical roles in cancer pathogenesis, owing to their capacity to regulate multiple cellular events, including growth, differentiation, apoptosis, and metastasis [36]. PTMs, including ubiquitination, phosphorylation, and acetylation, among others, are involved in the dysregulation of cellular physiology, thereby fostering proliferation, invasion, and metastasis in cancer cells [37]. Previous research has demonstrated that numerous TRIM proteins, predominantly functioning as E3 ubiquitin ligases, are significantly associated with cancer malignancy and prognosis through their regulation of substrate ubiquitination [38, 39]. As a significant TRIM protein, TRIM24 has been progressively investigated for its roles in various cancers, although research concerning its involvement in GC remains relatively limited. As shown above, aberrant TRIM24 upregulation was detected in cancerous tissue samples versus adjacent gastric tissue specimens, with high TRIM24 expression associated with poorer prognosis. Functional studies further demonstrated that TRIM24 markedly promoted cell proliferation and metastasis in GC, both in cultured cells and in animal models. Importantly, findings from the liver metastasis model indicated that the enhanced metastatic potential conferred by TRIM24 was not entirely dependent on primary tumor size. Mechanistically, this effect was mediated through the modulation of NRBP1 protein expression. These data indicate TRIM24 plays an oncogenic role and could be a therapeutic target in GC.

Tripartite motif (TRIM) proteins are an important subfamily of Really Interesting New Gene (RING) E3 ubiquitin ligases that control the ubiquitination of multiple protein substrates to regulate diverse biological processes. Tripartite motif-containing 24 (TRIM24), belonging to the tripartite motif protein family, features a RING domain as well as a C-terminal PHD-Bromo domain. Such unique configuration suggests its potential capability to recognize histone or non-histone proteins bearing particular combinations of post-translational modifications (PTMs) [40, 41]. TRIM24 has been reported to undergo aberrant activation across a diverse spectrum of cancers, e.g., hepatocellular carcinomas, and breast, prostate and lung cancers [42–46]. Numerous studies have indicated TRIM24 contributes to the regulation of tumor cell biology through interactions with tumor suppressor or oncogenic pathways. Specifically, TRIM24 promotes p53 ubiquitination and proteasome-dependent degradation [47], and also interacts with chromatin and estrogen receptor to induce target genes controlling cell division and carcinogenesis in breast cancer [41]. Additionally, TRIM24 induces tumorigenesis via activation of aerobic glycolysis [48] and undergoes chromosomal translocation resulting in the generation of oncogenic fusion proteins. However, in GC, investigations have primarily focused on surface phenomena such as Cyclin D1, AKT, and the Wnt/β-catenin signaling pathway [49, 50], while deeper mechanistic insights into TRIM24’s role in gastric cancer remain inadequately explored.

Human NRBP1(535 amino acids) has been shown to play dual roles in cancer progression, either suppressing or promoting tumor development. On the one hand, high levels of NRBP1 have been shown to enhance proliferation and metastasis in bladder cancer [31], as well as influence the oncogenic potential of triple-negative breast cancer cells via the P-Rex1/Rac1/Cdc42 pathway [51]. On the other hand, the tumor suppressor function of NRBP1 was first highlighted in hematological and intestinal cancers by gene knock-out studies in mice [28, 32]. Consistently, NRBP1 upregulation in colorectal cancer (CRC) is associated with improved survival, while NRBP1 overexpression in cultured CRC cells triggers apoptosis and inhibits cell proliferation, and decreases xenograft growth in mice [32]. Likewise, NRBP1 upregulation is also correlated with good prognosis in lung adenocarcinoma [28]. Therefore, the tumor suppressive function of NRBP1 exhibits a certain degree of credibility. In this study, TRIM24 physically interacted with NRBP1 and enhanced ubiquitination-degradation of NRBP1, inhibiting cell apoptosis. This theory has been substantiated by several experiments as follows. Firstly, TRIM24 silencing upregulated NRBP1 in GC cells, whereas TRIM24 overexpression downregulated NRBP1. Secondly, in the presence of proteasome inhibitors, MG132 treatment disrupted TRIM24-induced NRBP1 downregulation in GC cells. Mechanistically, phosphorylation at the S42 residue may be a critical prerequisite for TRIM24 to recognize and degrade NRBP1, thereby modulating its tumor-suppressive function. Additionally, TRIM24 can promote NRBP1 ubiquitination at the K430 residue, subsequently leading to its degradation, ultimately inhibiting apoptosis in gastric cancer cells.

Apoptosis has specific cellular characteristics and includes extrinsic and intrinsic pathways [52, 53]. Existing evidence highlights an important regulatory role for TRIM proteins in intrinsic pathways, which substantially affects the cell fate [54]. TRIM3 was negatively associated with the expression of Bcl-2 as a pro-apoptotic gene in GC patients [55]. In addition, shTRIM21 remarkably suppressed apoptosis in apatinib-treated GC cells, while TRIM21 upregulation further induced apoptosis in GC cells after apatinib culture [56]. Importantly, to date, a total of three articles have elaborated the relationship between GC and the TRIM24 protein [49, 50, 57]. Among them, only Fang et al. [49] studied the relationship between TRIM24 and apoptosis, reporting that low expression of TRIM24 increases the number of apoptotic cells, corroborating the present findings. However, the mechanisms underpinning TRIM24-induced apoptosis were not elucidated. In this study, TRIM24 silencing substantially triggered apoptosis in GC cells, whereas downregulation of NRBP1 significantly attenuated this effect. Consistent with prior work showing NRBP1 promotes caspase-dependent intrinsic apoptosis via JNK signaling, stabilization of NRBP1 upon TRIM24 inhibition in GC cells was accompanied by increased p-JNK, cytochrome-c release, and caspase-9/-3 activation. Collectively, these findings imply that TRIM24, by directly targeting NRBP1, enhances the malignant potential of cells while suppressing apoptosis in GC.

**Fig. 9:**
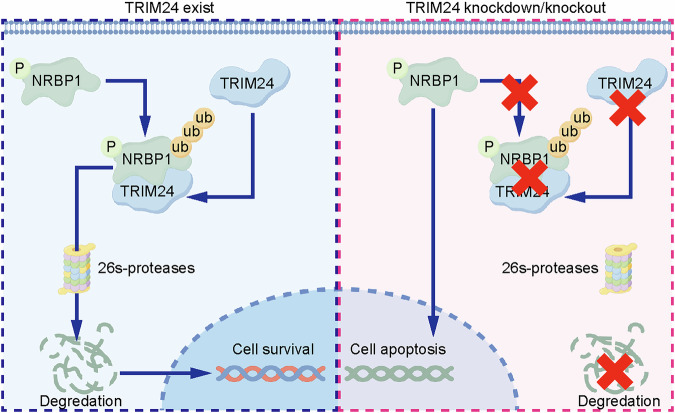
The mechanism of TRIM24 promotes ubiquitination of NRBP1, resulting GC cells apoptosis. This figure is created with figdraw.com.

In summary, the above findings indicated that TRIM24 plays pro-tumorigenic roles in GC progression via S42 phosphorylation and K430 ubiquitination–related degradation of NRBP1. Notably, S42 or K430 mutant of NRBP1 suppresses TRIM24’s capability of degrading NRBP1, enhancing GC proliferation, invasion and metastasis (Fig. 9). We are the first to demonstrate that TRIM24 promotes gastric cancer progression through the degradation of NRBP1, a tumor suppressor protein. This discovery provides new mechanistic insights into how TRIM24 exerts its oncogenic effects and suggests that targeting TRIM24 or enhancing NRBP1 expression could serve as promising therapeutic strategies for the treatment of gastric cancer.

## Supplementary information

Supplementary figures and table

aj-checklis

All western blots -supplementary materials

## Data Availability

Data will be made available on request.
