# Supplementary material for: TRIM24 promotes proliferation and metastasis of gastric cancer via mediating NRBP1 ubiquitination
Source: Cell Death Dis. 2025 Dec 22;16(1):915. doi: 10.1038/s41419-025-08346-w (PMC12749008; doi:10.1038/s41419-025-08346-w)
Supplement: Supplementary file 1 — Supplementary figures and table [file 41419_2025_8346_MOESM1_ESM.docx]

**
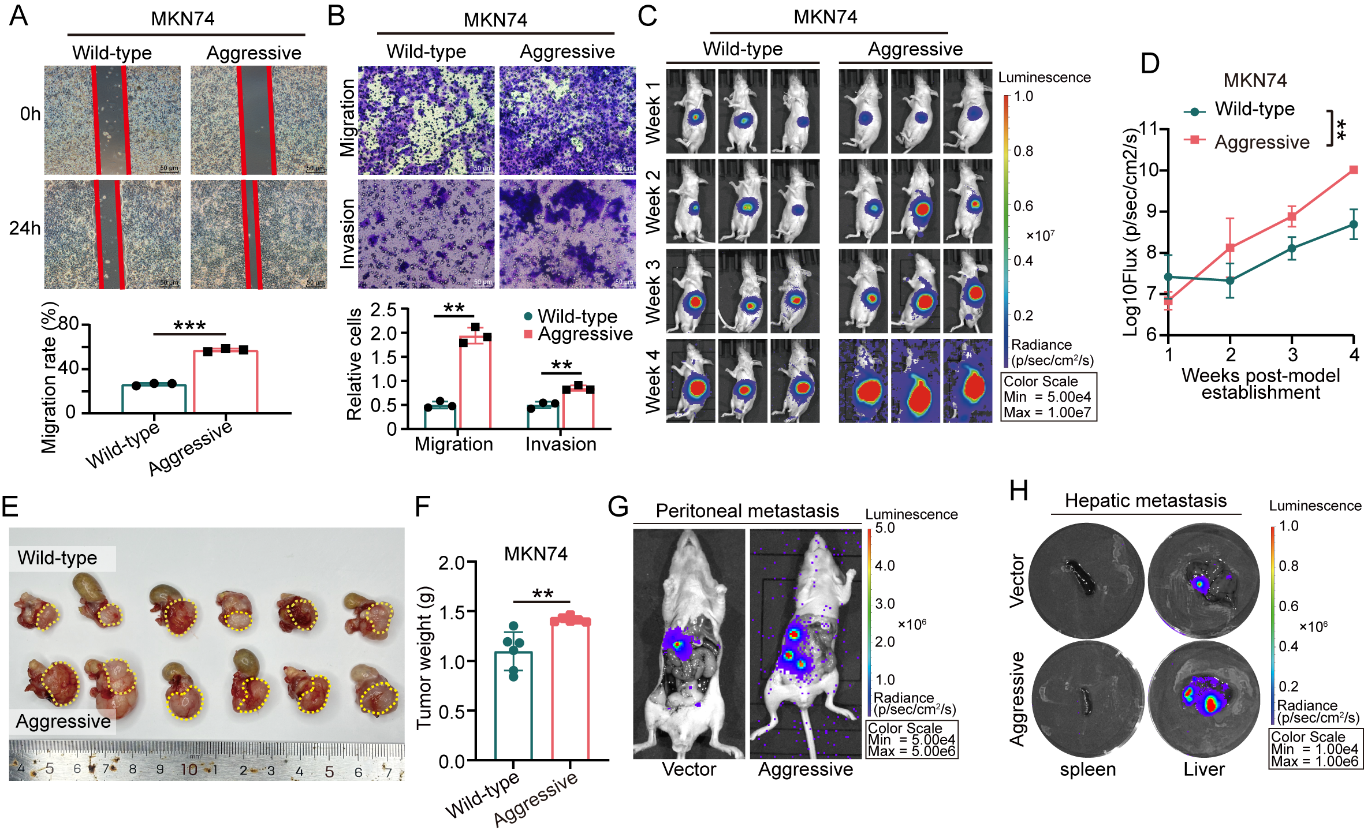
Supplementary figure 1. Aggressive GC Cells Exhibit Enhanced Metastatic and Growth Abilities. (A)** Aggressive GC Cells Exhibit Enhanced Metastatic and Growth Abilities. A: Representative images of scratch assays and corresponding quantification showing the migratory capacity of wild-type MKN74 and aggressive-MKN74 cells. **(B)** Transwell assay results demonstrating the migration and invasion abilities of wild-type MKN74 and aggressive-MKN74 cells, with statistical quantification. **(C)** Representative orthotopic fluorescence images of tumors derived from wild-type MKN74 and aggressive-MKN74 mice modles. **(D)** Quantification of fluorescence intensity for orthotopic tumors from wild-type MKN74 and aggressive-MKN74 mice modles. **(E)** Images of excised orthotopic tumors from wild-type MKN74 and aggressive-MKN74 mice modles. **(F)** Tumor weight comparison between wild-type MKN74 and aggressive-MKN74 mice modles. **(G)** Fluorescence imaging of peritoneal metastases in mice in wild-type MKN74 and aggressive-MKN74 mice modles. **(H)** Fluorescence imaging of liver and spleen metastases in wild-type MKN74 and aggressive-MKN74 mice modles. Data represent mean ± SD (n =3). *P < 0.05, **P < 0.01, ***P < 0.001.


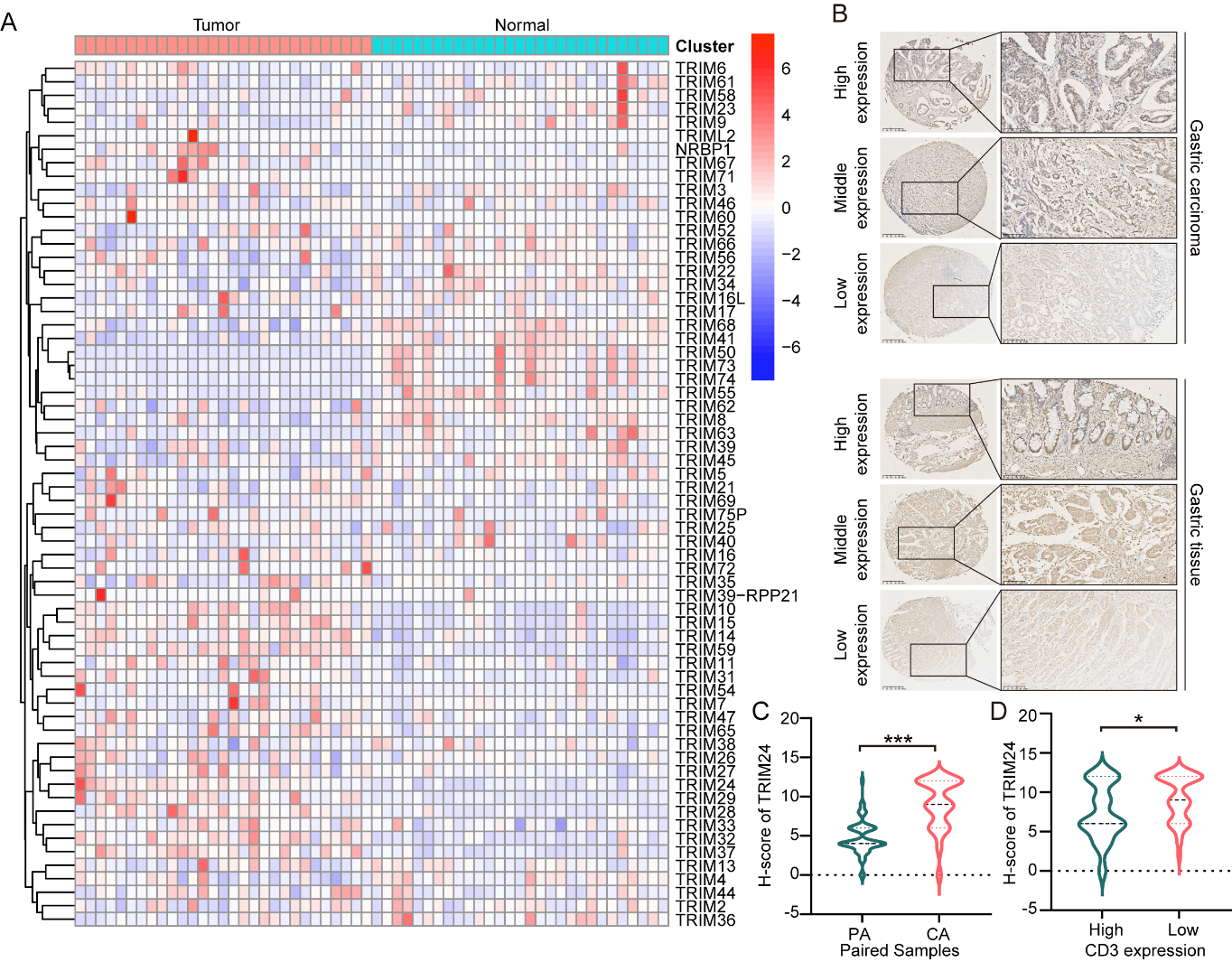


**Supplementary figure 2. TRIM24 is overexpressed in GC tumor tissues and TRIM24 overexpression predicts poorer prognosis. (A)** Heat map of relative expression levels of total TRIM family mRNAs in 29 GC tissues and paired adjacent tissues. **(B)** Representative images of TRIM24 protein expression in GC and adjacent tissues in three groups (low, medium and high expression groups) according to the IHC score. **(C)** A statistical chart showing differences in TRIM24 IHC scores between GC tissues and unpaired adjacent tissues. **(D)** A statistical chart showing differences in TRIM24 IHC scores between difference CD3 expression level. *, p < 0.05; **, p < 0.01; ***, p < 0.001.

**
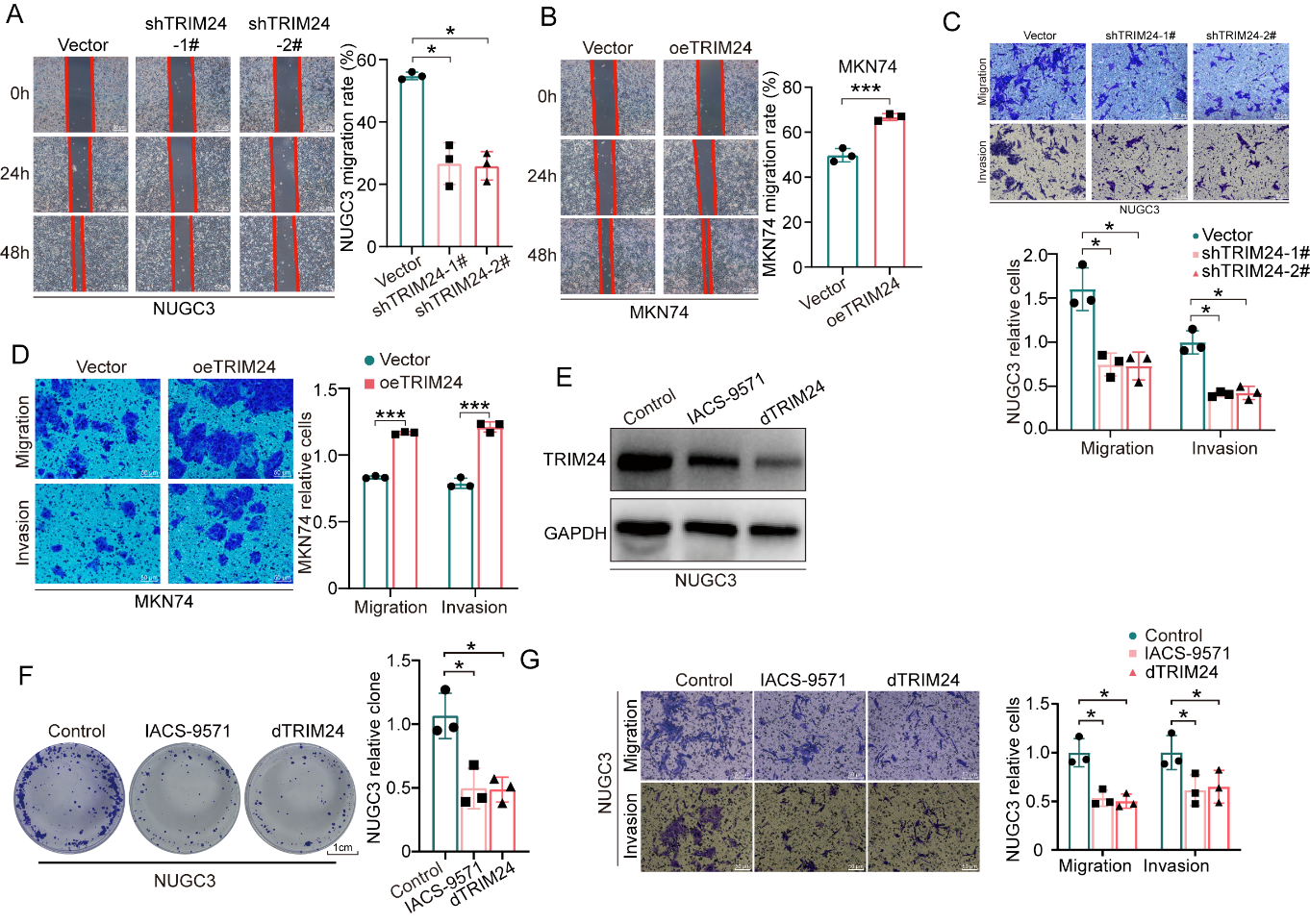
**

**Supplementary figure 3. TRIM24 facilitates GC cell migration, invasion in vitro.** Wound healing **(A-B)**, and migration and invasion **(C-D)** were performed in NUGC3 and MKN74 cells transfected with vector lentivirus, TRIM24 shRNA lentiviruses (1# and 2#), and/or TRIM24-overexpressing lentiviruses, respectively (n=3). **(E)** Western blot analysis of NUGC3 cells treated with TRIM24 inhibitors IACS-9571 (1 μM) and dTRIM24 (0.5 μM) for 24 hours. **(F-G)** Colony formation and Transwell assays, along with corresponding quantification, showing the effects of TRIM24 inhibitors on NUGC3 cells. Data represent mean ± SD (n = 3). *P < 0.05, **P < 0.01, ***P < 0.001.


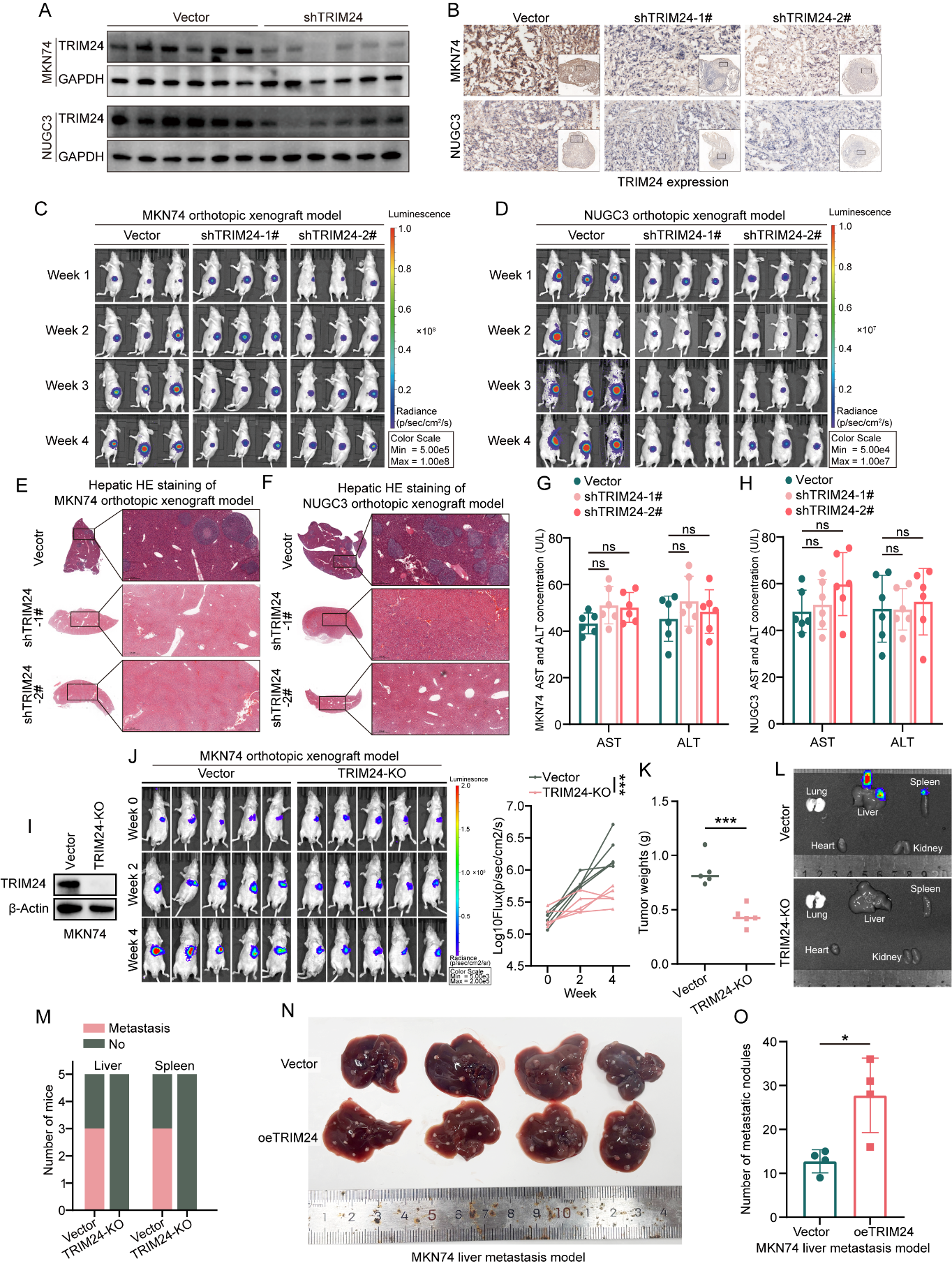


**Supplementary figure 4. TRIM24 promotes tumor proliferation, invasion and metastasis in *vivo*. (A-B)** TRIM24 expressions in orthotopic xenograft tumor of MKN74 and NUGC3 transfected with vector lentivirus and TRIM24 shRNA lentiviruses (1# and 2#) were verified by WB and IHC assays. **(C-D)** The fluorescence images (representative) of tumor-bearing mice were obtained and detected in the vector and shTRIM24 (#1 and #2) groups using the IVIS imaging system. **(E-F)** The HE staining were performed in MKN74 and NUGC3 hepatic metastatic nodules in vector lentivirus, TRIM24 shRNA lentiviruses (1# and 2#) groups. transfected with, and/or TRIM24-overexpressing lentiviruses. **(G-H)** The AST and ALT levels in peripheral blood were detected. **(I)** Western blot analysis confirming TRIM24 knockout efficiency in MKN74 cells using the CRISPR-Cas9 system. **(J)** The fluorescence images and values of MKN74 tumor-bearing mice were obtained and detected in the vector and knockout TRIM24 (TRIM2-KO) groups using the IVIS imaging system. **(K)** Tumor weights in the vector and TRIM2-KO groups. **(L-M)** Representative images of hepatic and spleen metastasis and numbers of mice with hepatic and splenic metastasis in the vector and TRIM24-KO groups. **(N-O)** Images of liver metastases and quantification of liver metastatic nodules in the liver metastasis model. Data are shown as mean ± SD. ns: no statistical difference, **P* < 0.05, ***P* < 0.01, ****P* < 0.001.

**
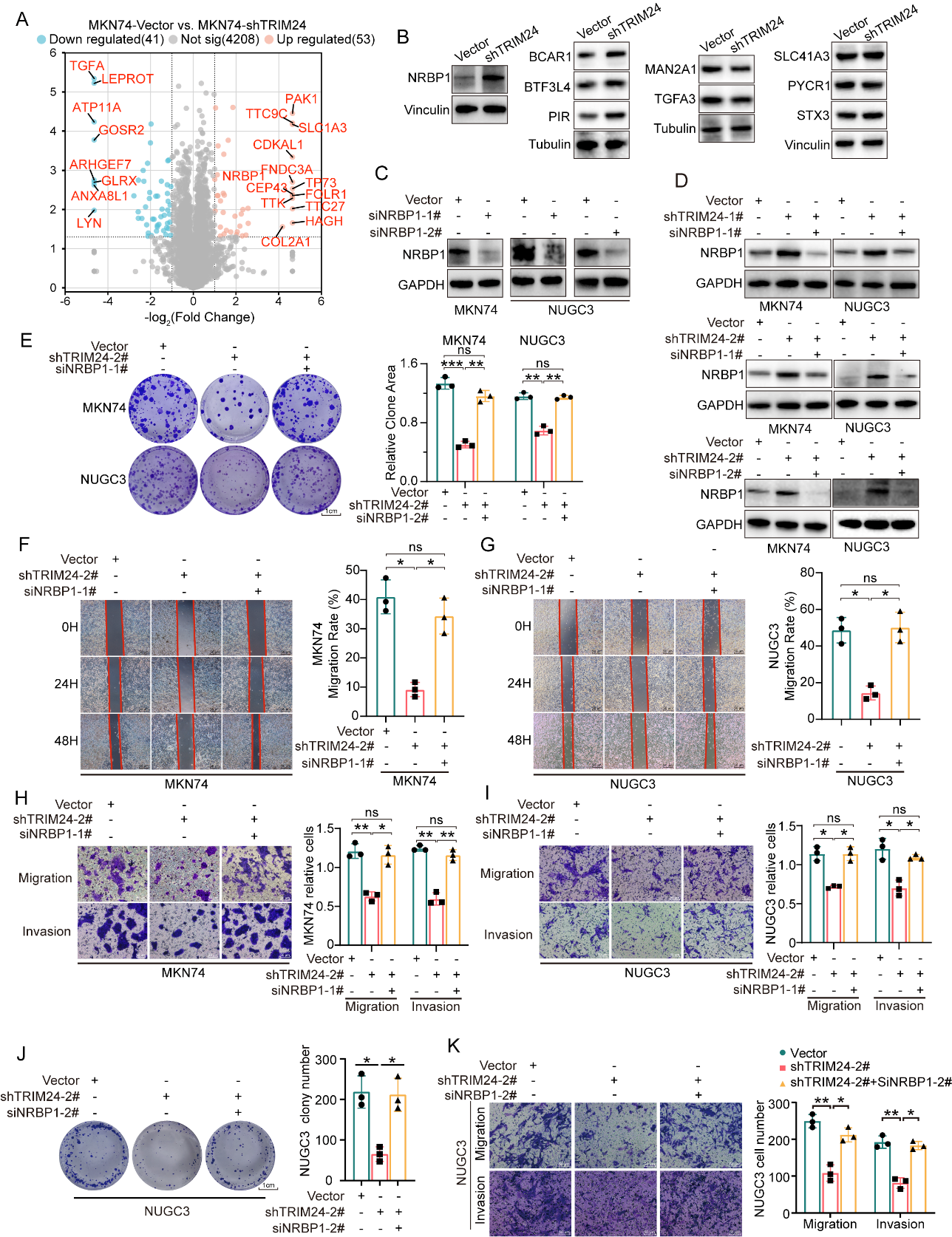
**

**Supplementary figure 5. Proteomic analysis of MKN74 and NUGC3 GC cells indicates NRBP1 is the key effector in TRIM24-mediated malignancy of GC. (A)** Volcano plot showing differentially expressed proteins in MKN74 cells. **(B)** The protein expressions of NRBP1, BCAR1, BTF3L4, PIR, MAN2A1, TGFA3, SLC41A3, PYCR1, and STX3 were evaluated in MKN74 cells transfected with TRIM24 shRNA lentivirus by WB. **(C-D)** Western blot analysis showing the knockdown efficiency of siNRBP1-1# and siNRBP1-2# in wild-type GC cells **(C)** or shTRIM24 GC cells **(D)**. **(E-I)** TRIM24 knockdown Colony forming **(E)**, wound healing **(F-G)**, migration and invasion **(H-I)** assays were performed in MKN74 and NUGC3 cells transfected with TRIM24 shRNA-2# lentivirus alone or TRIM24 shRNA-2# lentivirus and NRBP1 siRNA-1# simultaneously. (**J-K)** Clone formation, migration, and invasion assays were performed in cells transfected with TRIM24 shRNA-2# lentivirus in combination with NRBP1 siRNA-2#. Data represent mean ± SD (n = 3). *P < 0.05, **P < 0.01, ***P < 0.001.

**
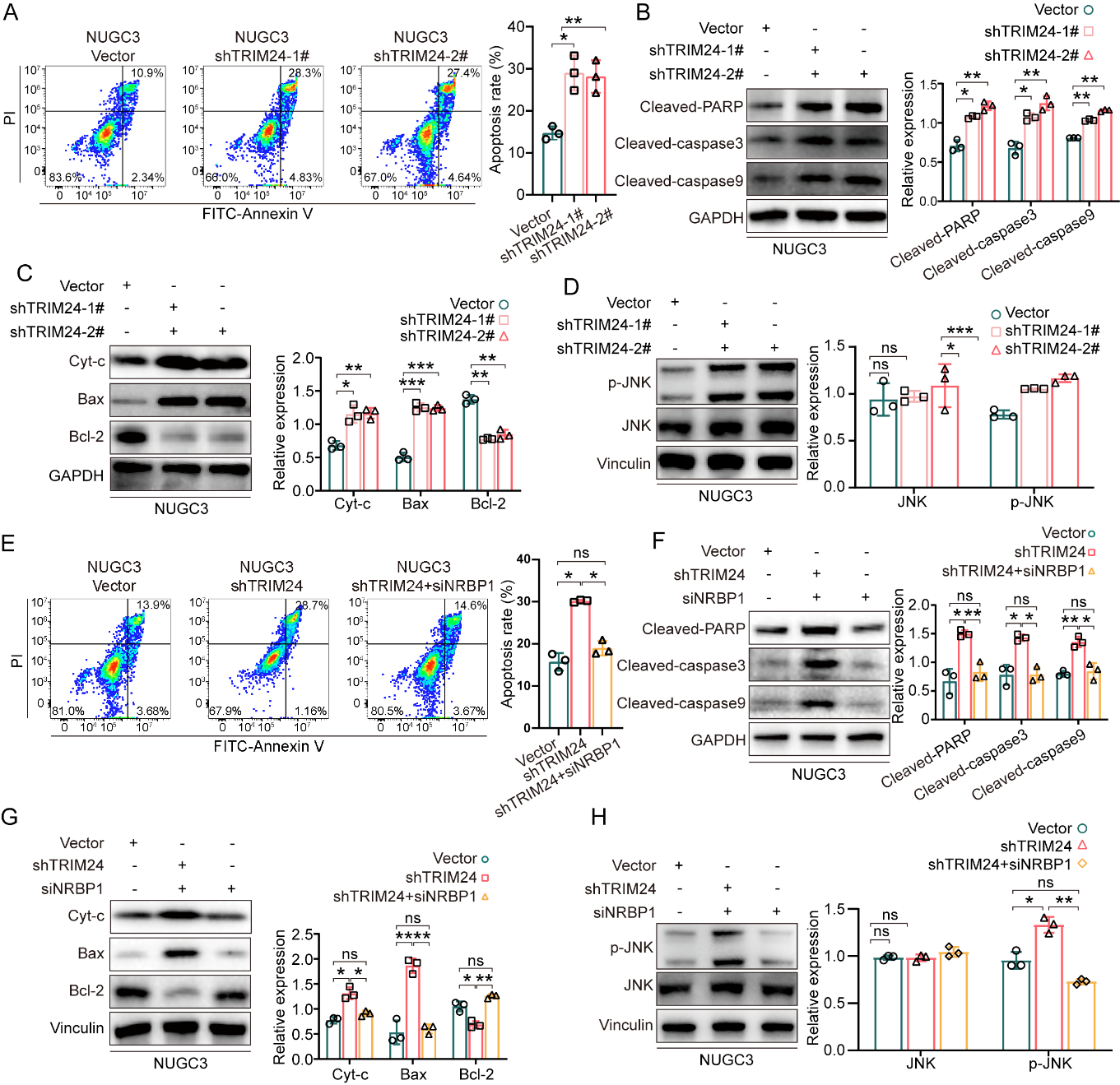
**

**Supplementary figure 6. Knockdown of TRIM24 induces apoptosis through increasing NRBP1 expression in NUGC3 cells. (A)** Apoptotic cell analysis by flow cytometry after transfection with TRIM24 shRNA (1# and 2#) lentivirus in NUGC3. Annexin V-FITC and PI were used to assess apoptotic events. **(B-C)** Protein expression levels of the intrinsic apoptosis markers clv-PARP, clv-caspase3, clv-caspase9, Cyt-c, Bax, and Bcl-2 in NUGC3 and after TRIM24 knockdown. **(D)** Protein expression levels of JNK and p-JNK in NUGC3 after TRIM24 knockdown. **(E)** Apoptotic cell analysis by flow cytometry after transfection with TRIM24 shRNA lentivirus alone or simultaneously transfected with TRIM24 shRNA lentivirus and NRBP1 siRNA in NUGC3. Annexin V-FITC and PI were used to assess apoptotic events. **(F-G)** Protein expression levels of the intrinsic apoptosis markers clv-PARP, clv-caspase 3, clv-caspase 9, Cyt-c, Bax, and Bcl-2 in NUGC3 after transfection with TRIM24 shRNA lentivirus alone or simultaneously transfected with TRIM24 shRNA lentivirus and NRBP1 siRNA. **(H)** Protein expression levels of JNK and p-JNK in NUGC3 after transfection with TRIM24 shRNA lentivirus alone or simultaneously transfected with TRIM24 shRNA lentivirus and NRBP1 siRNA. Data represent mean ± SD (n = 3). *P < 0.05, **P < 0.01, ***P < 0.001.


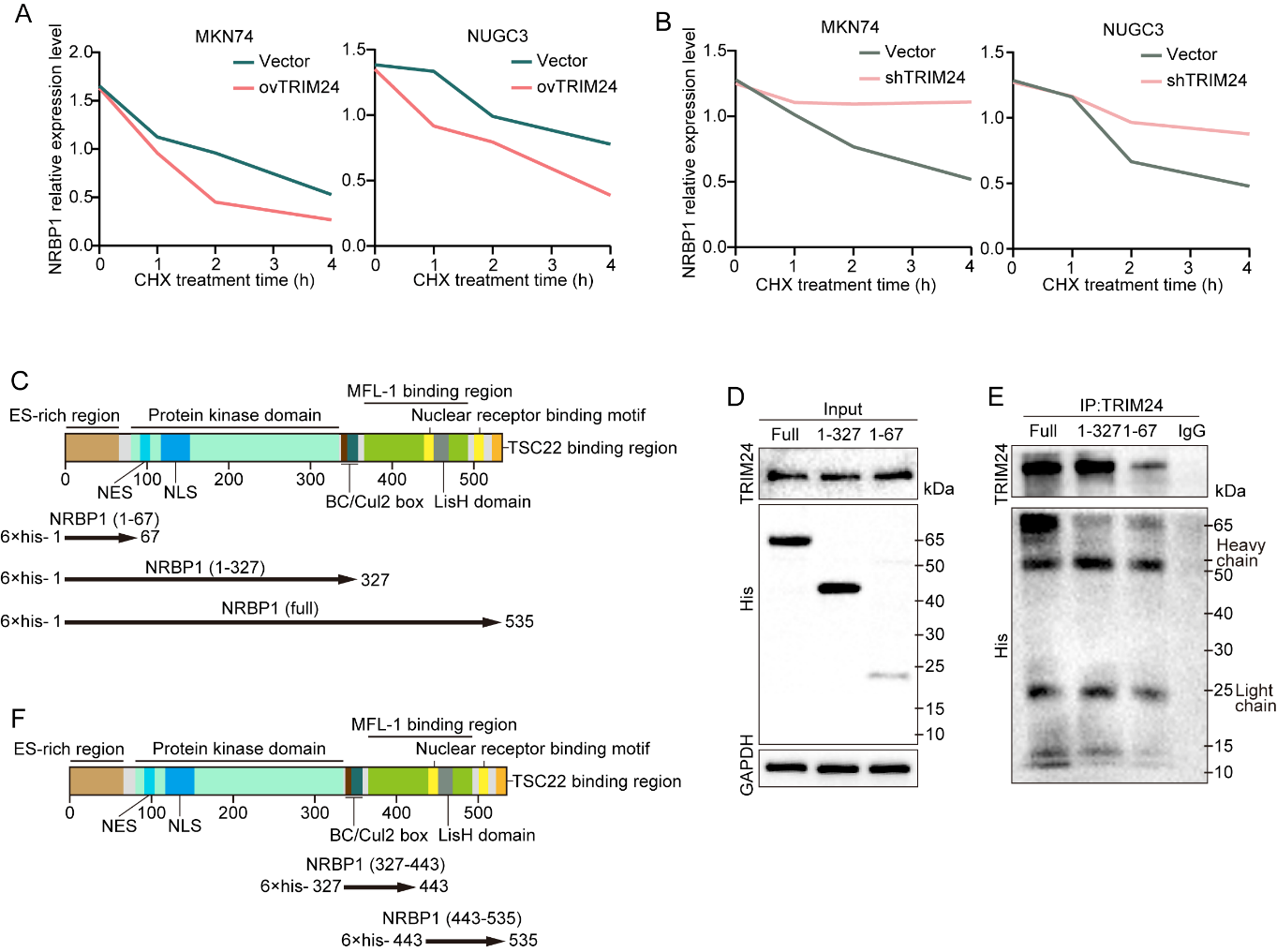


**Supplementary figure 7.** **TRIM24 interacted with NRBP1 at 327-535 region. (A-B)** Relative expression levels of NRBP1 protein as shown in figure 7B-C. **(C, F)** The Schematic representation of the NRBP1 truncations. **(D-E)** The plasmids of NRBP1 truncations were transfected into HEK293T cells, and further expression and interaction were detected by co-IP and WB assays.


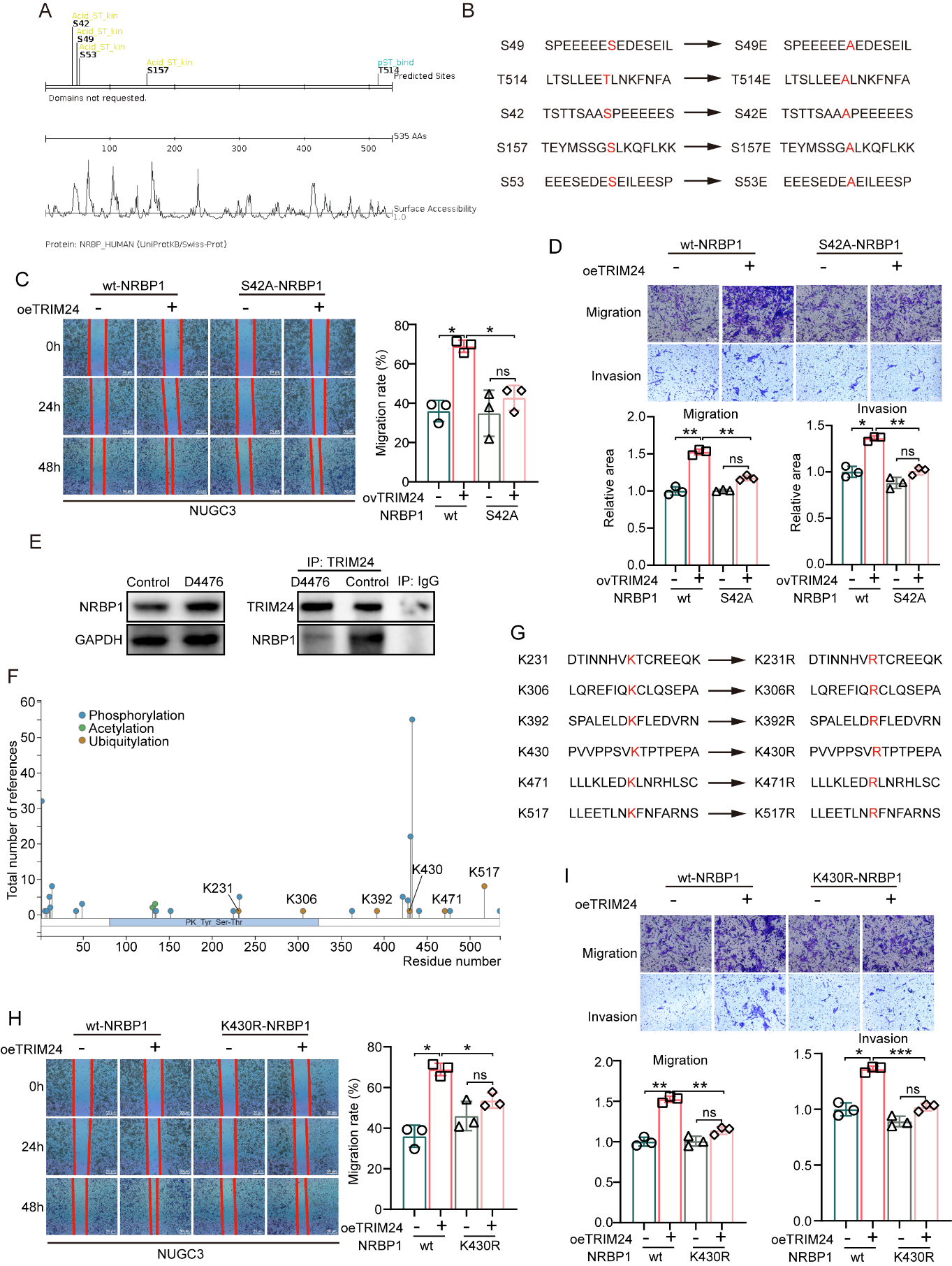


**Supplementary figure 8. TRIM24 promotes ubiquitination of S42 phosphorylated NRBP1 at the K430 residue. (A)** Site map of NRBP1 phosphorylation site prediction. **(B)** Schematic diagram of NRBP1 phosphorylation site mutation to Alanine. Wound healing **(C)**, migration and invasion **(D)** assays were performed in NUGC3 (vector or overexpression TRIM24) transfected with NRBP1 S42 site mutation plasmid. **(E)** HEK293T cells were treated with the CK1 inhibitor D4476 (60 μM) for 6 hours, followed by immunoprecipitation and WB analysis. **(F)**  Site map of NRBP1 ubiquitination site prediction. **(G)** Schematic diagram of NRBP1 ubiquitination site mutation to arginine. Wound healing **(H)**, migration and invasion **(I)** assays were performed in NUGC3 (vector or overexpression TRIM24) transfected with NRBP1 S42 site mutation plasmid.  Data are shown as mean ± SD. ns: no statistical difference, **P* < 0.05, ***P* < 0.01, ****P* < 0.001.

**Supplementary Table S1**

Detailed information on relevant antibodies

| **Antibody name** | **Commercial source** | **Catalog number** |
| --- | --- | --- |
| TRIM24 | Abcam | ab70560 |
| TRIM24 | Proteintech | 66324-1-Ig |
| STX3 | Proteintech | 66760-1-Ig |
| SLC41A3 | Invitrogen | PA5-107267 |
| TGFA | abcam | ab208156 |
| MAN2A1 | HUABIO | PSH02-67 |
| PYCR1 | abcam | ab317563 |
| PIR | Proteintech | 10263-1-AP |
| BTF3L4 | Proteintech | 16500-1-AP |
| BCAR1 | abcam | ab108320 |
| NRBP1 | Invitrogen | MA5-24925 |
| NRBP1 | Invitrogen | PA5-30542 |
| Cyp24a1 | Proteintech | 21582-1-AP |
| SPC25 | Proteintech | 26474-1-AP |
| IFIT1 | Proteintech | 23247-1-AP |
| APOL1 | Proteintech | 11486-2-AP |
| UBE2T | abcam | ab179802 |
| RRM2 | Proteintech | 11661-1-AP |
| HSPA8 | Proteintech | 66442-1-Ig |
| GAPDH | Proteintech | 60004-1-Ig |
| Vinculin | HUABIO | ET1705-94 |
| Tubulin | abcam | ab7291 |
| clv-PARP | HUABIO | ET1608-10 |
| clv-caspase3 | Cell Signaling Technology | 9661T |
| clv-caspase9 | Cell Signaling Technology | 9505T |
| Cyt-c | HUABIO | ET1610-16 |
| Bax | HUABIO | ET1603-34 |
| Bcl-2 | Cell Signaling Technology | 15071T |
| JNK | Proteintech | 24164-1-AP |
| P-JNK | Proteintech | 80024-1-RR |
| Ubiquitin | Cell Signaling Technology | 20326 |
| DYKDDDDK tag | Proteintech | 20543-1-AP |
