# Supplementary material for: TRIM24 promotes proliferation and metastasis of gastric cancer via mediating NRBP1 ubiquitination
Source: Cell Death Dis. 2025 Dec 22;16(1):915. doi: 10.1038/s41419-025-08346-w (PMC12749008; doi:10.1038/s41419-025-08346-w)

Figure1 H and I

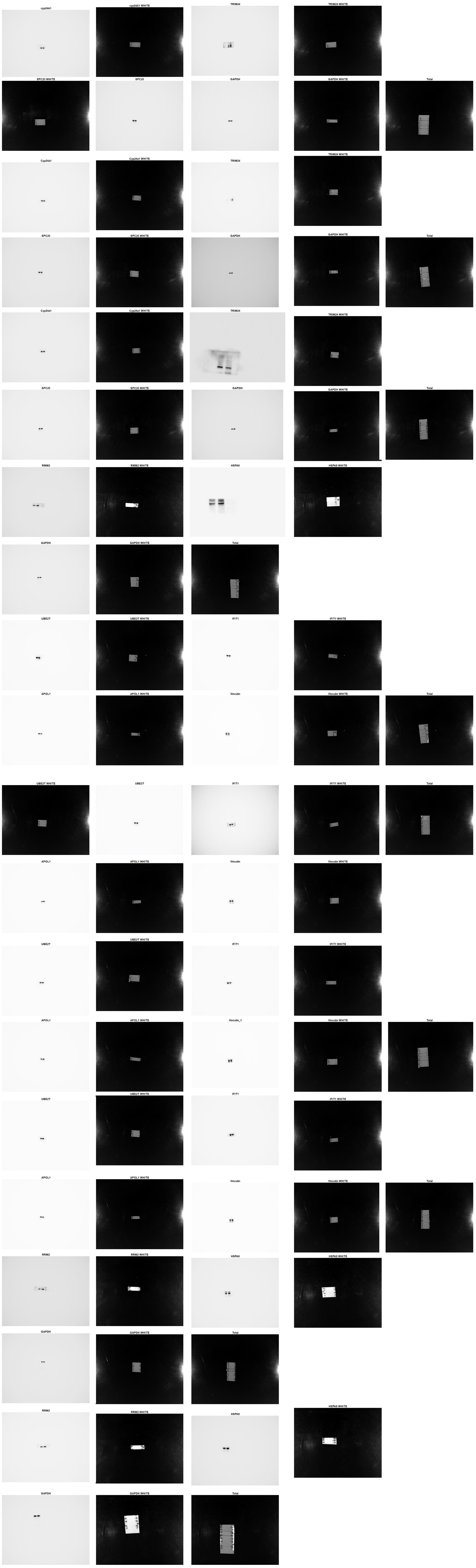

Figure 2G

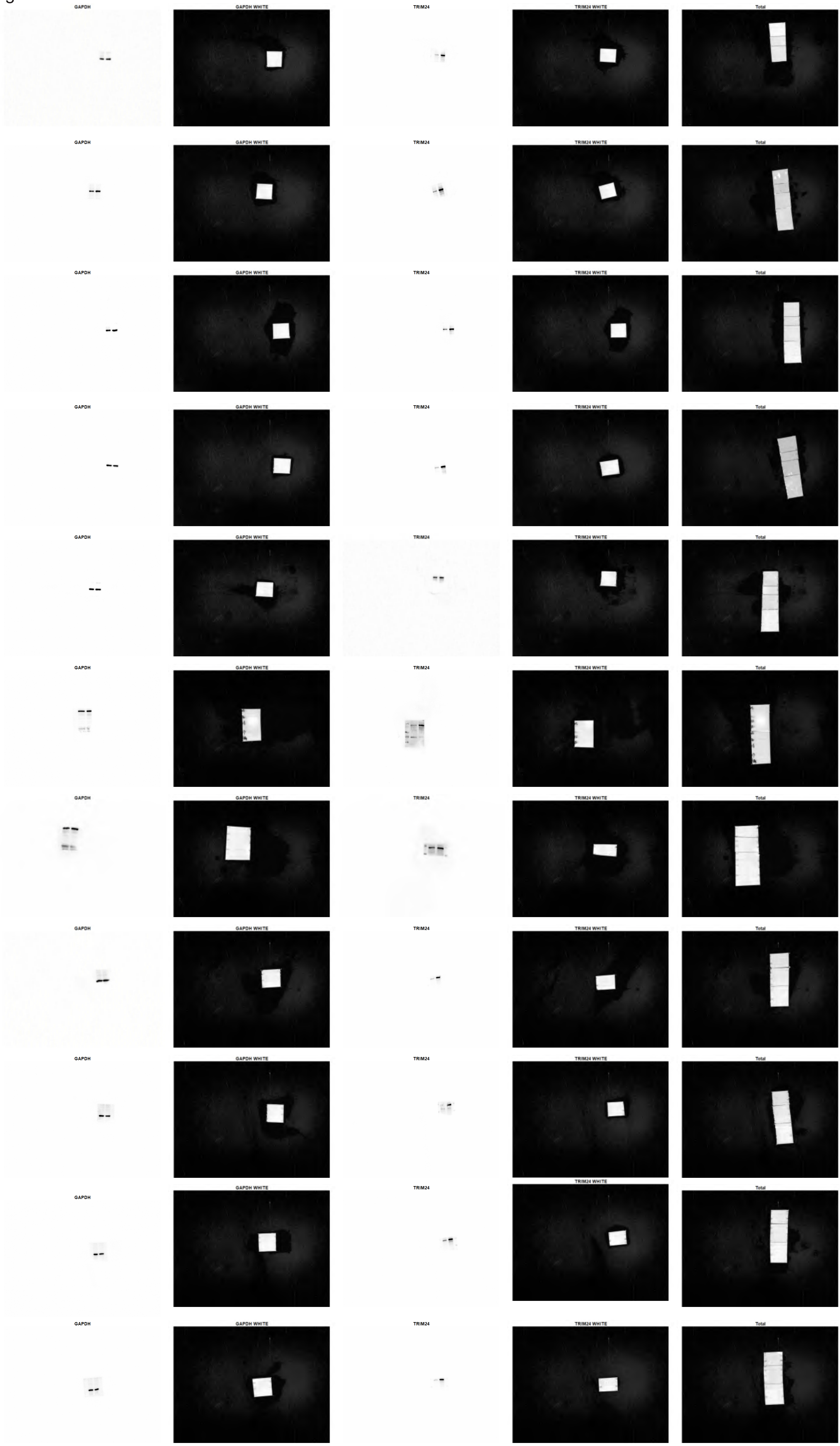
GAPDH
GAPDH WHITE
TRIM24
TRIM24 WHITE
Total
GAPDH
GAPDH WHITE
TRIM24
TRIM24 WHITE
Total
GAPDH
GAPDH WHITE
TRIM24
TRIM24 WHITE
Total
GAPDH
GAPDH WHITE
TRIM24
TRIM24 WHITE
Total
GAPDH
GAPDH WHITE
TRIM24
TRIM24 WHITE
Total
GAPDH
GAPDH WHITE
TRIM24
TRIM24 WHITE
Total
GAPDH
GAPDH WHITE
TRIM24
TRIM24 WHITE
Total
GAPDH
GAPDH WHITE
TRIM24
TRIM24 WHITE
Total
GAPDH
GAPDH WHITE
TRIM24
TRIM24 WHITE
Total
GAPDH
GAPDH WHITE
TRIM24
TRIM24 WHITE
Total
GAPDH
GAPDH WHITE
TRIM24
TRIM24 WHITE
Total

Figure3A

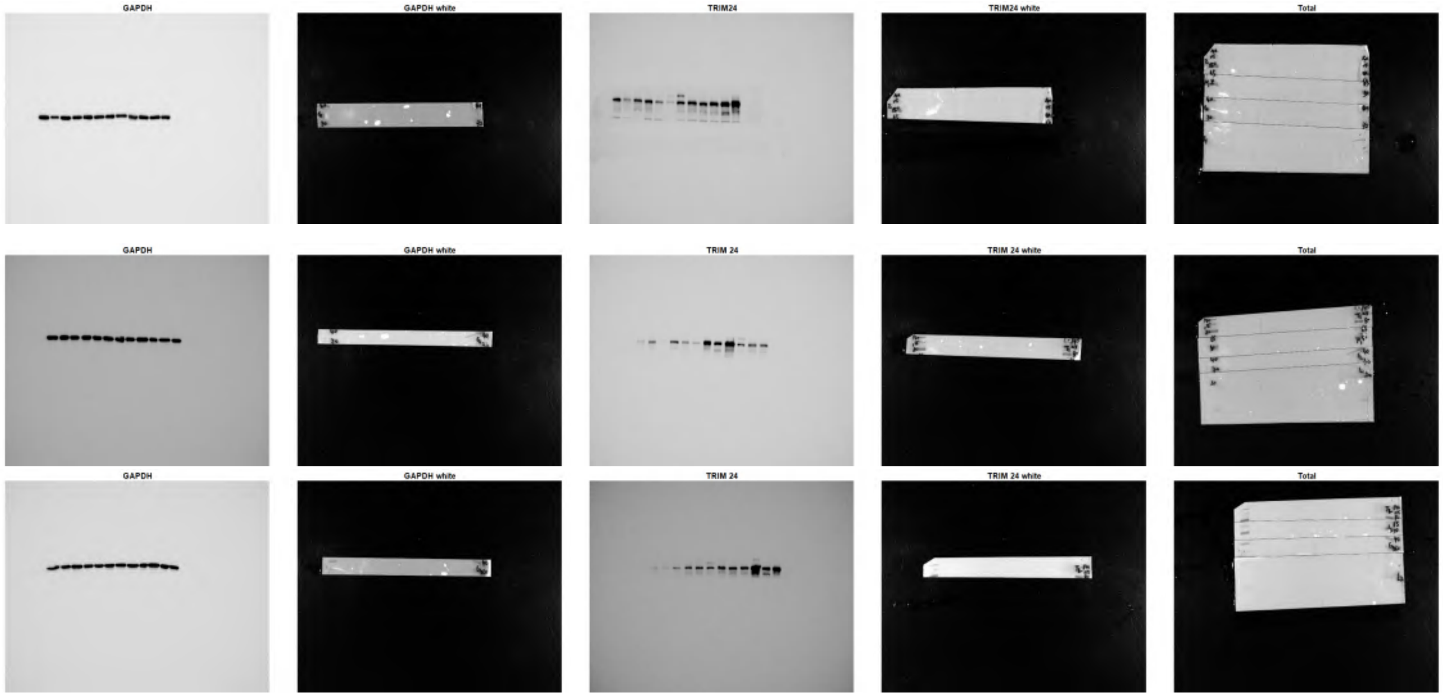

Figure3B

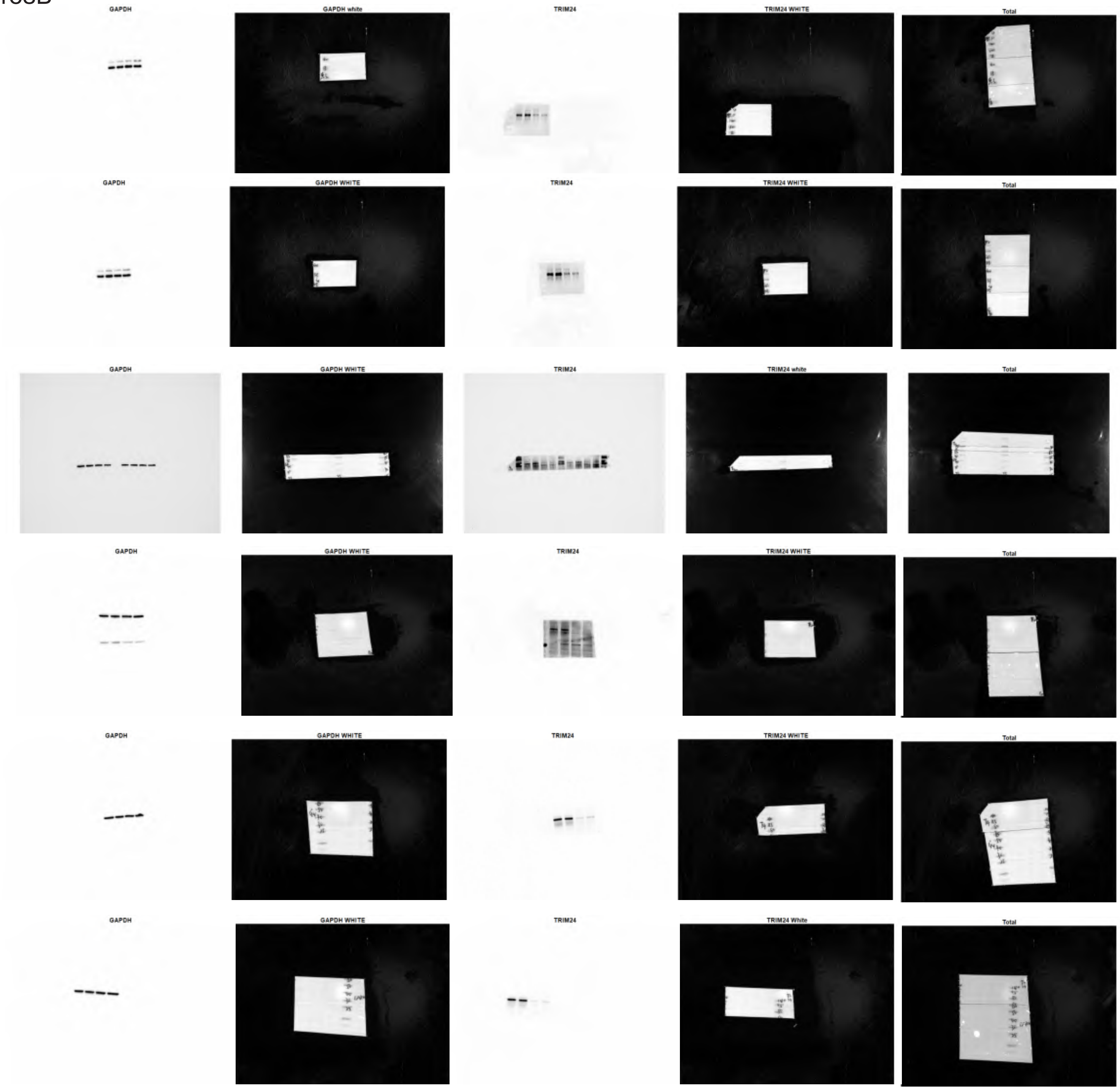

Figure3C

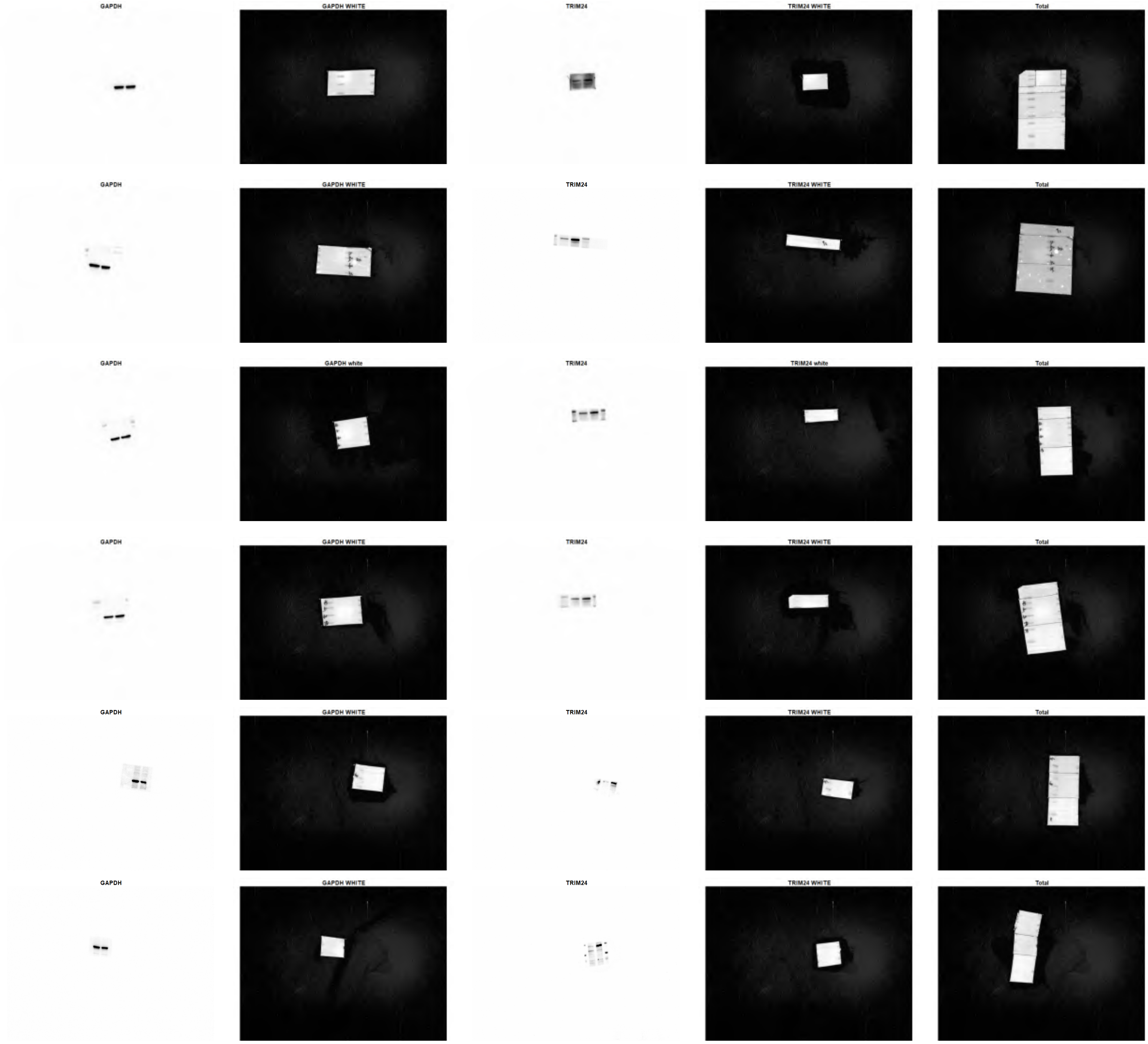
GAPDH
GAPDH WHITE
TRIM24
TRIM24 WHITE
Total
GAPDH
GAPDH WHITE
TRIM24
TRIM24 WHITE
Total
GAPDH
GAPDH white
TRIM24
TRIM24 white
Total
GAPDH
GAPDH WHITE
TRIM24
TRIM24 WHITE
Total
GAPDH
GAPDH WHITE
TRIM24
TRIM24 WHITE
Total
GAPDH
GAPDH WHITE
TRIM24
TRIM24 WHITE
Total

Figure3D

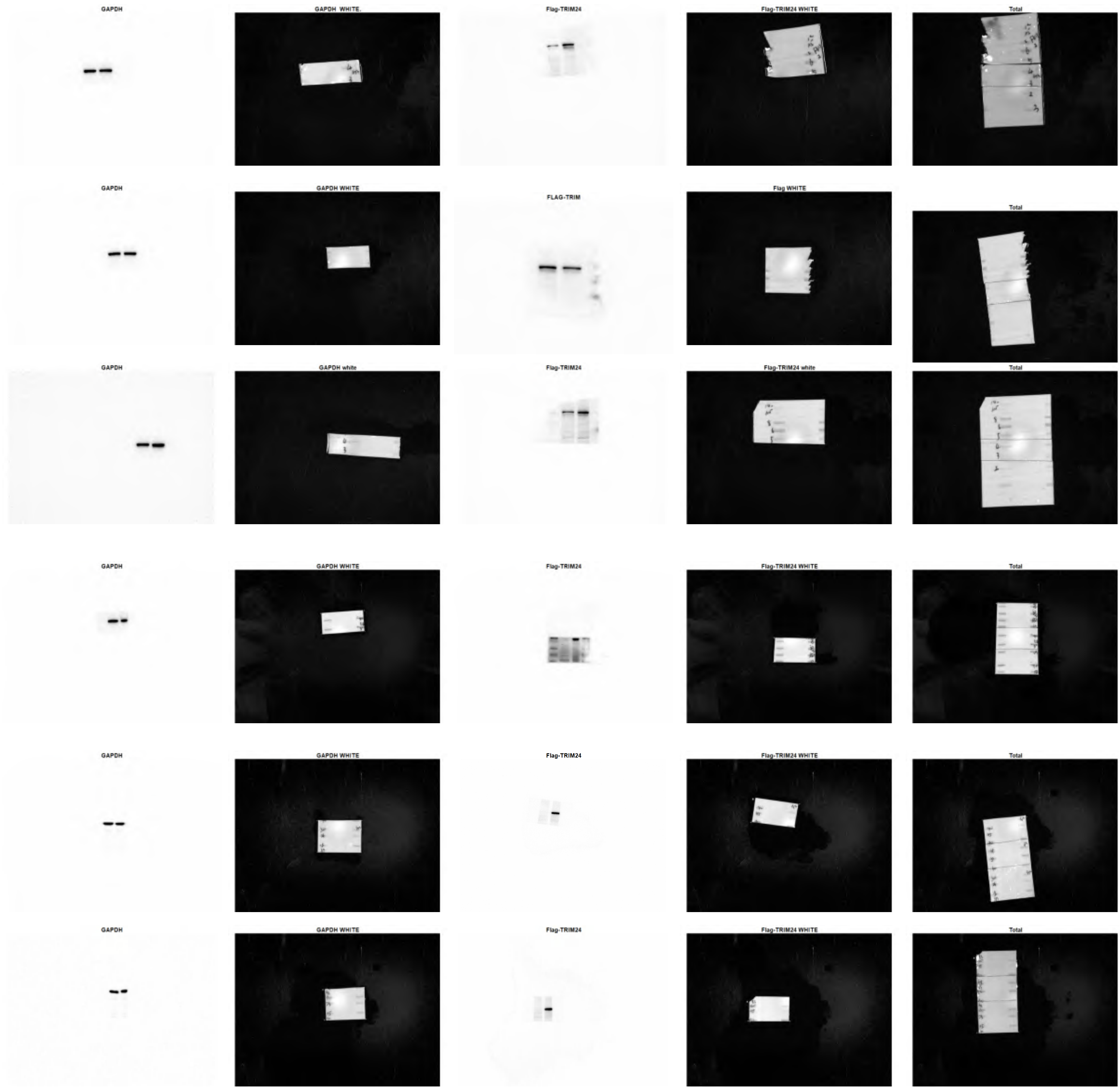
GAPDH
GAPDH WHITE.
Flag-TRIM24
Flag-TRIM24 WHITE
Total
GAPDH
GAPDH WHITE
FLAG-TRIM
Flag WHITE
Total
GAPDH
GAPDH white
Flag-TRIM24
Flag-TRIM24 white
Total
GAPDH
GAPDH WHITE
Flag-TRIM24
Flag-TRIM24 WHITE
Total
GAPDH
GAPDH WHITE
Flag-TRIM24
Flag-TRIM24 WHITE
Total
GAPDH
GAPDH WHITE
Flag-TRIM24
Flag-TRIM24 WHITE
Total

Figure5D

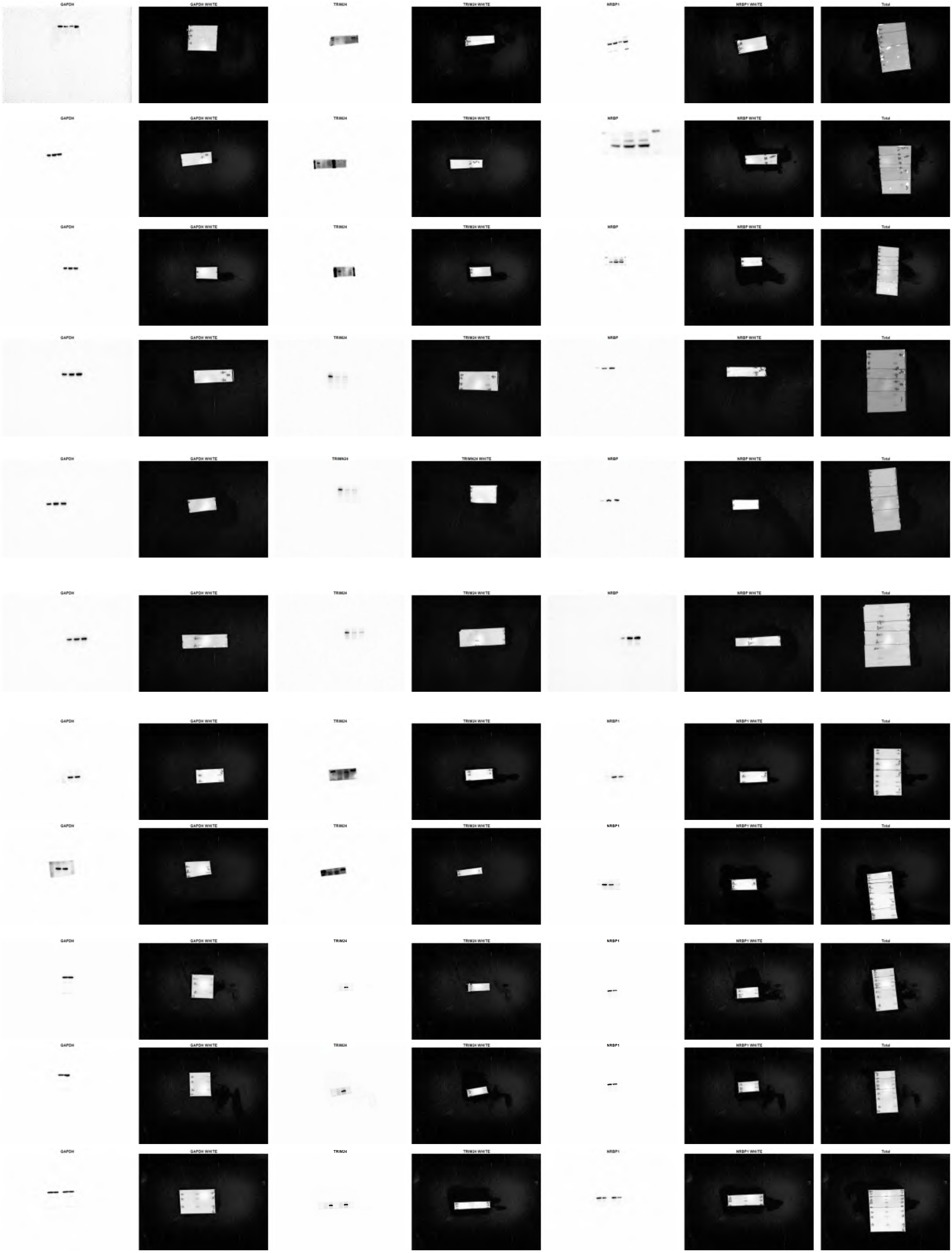

Figure5E

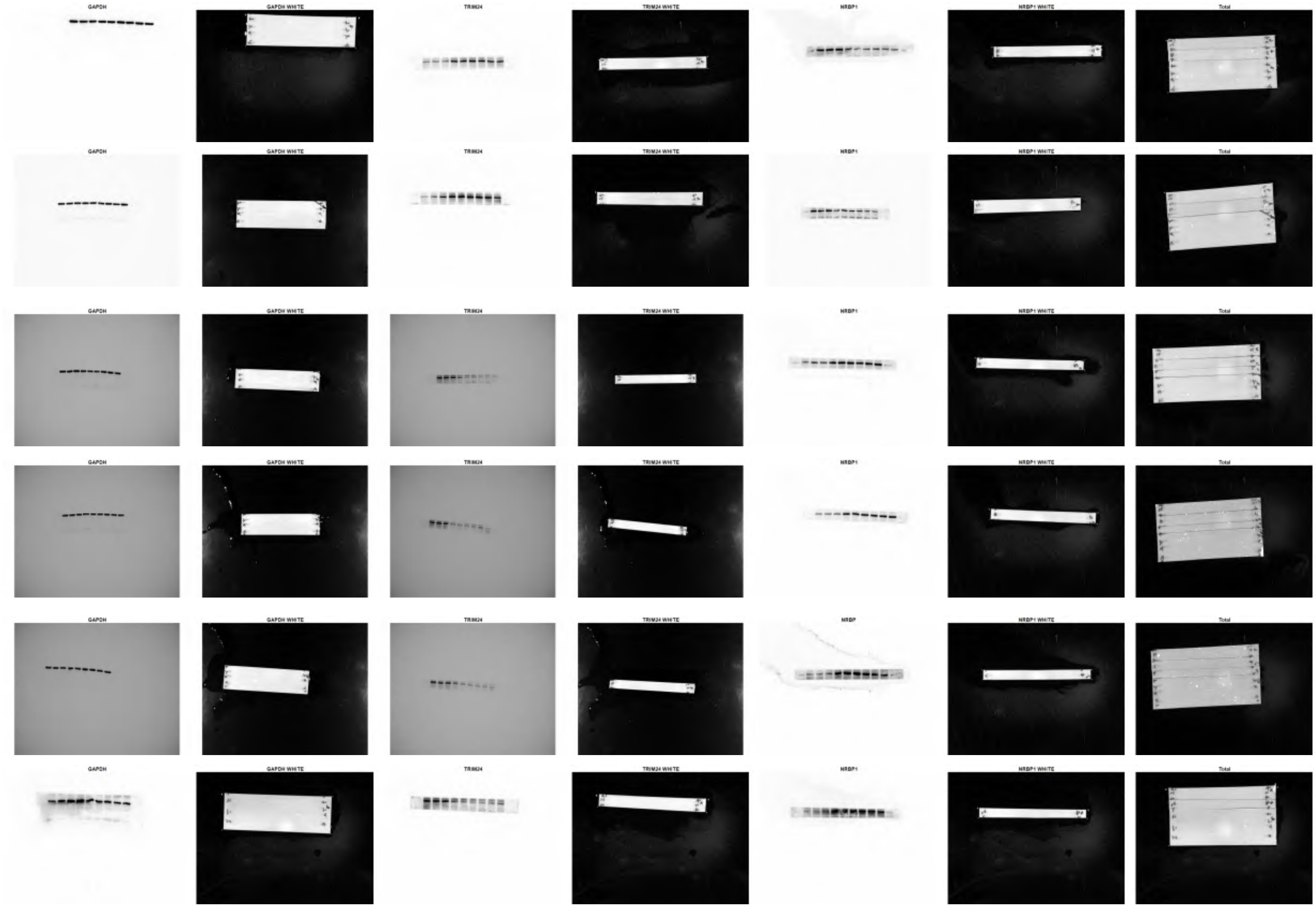
GAPDH
GAPDH WHITE
TRIM24
TRIM24 WHITE
NRBP1
NRBP1 WHITE
Total
NRBP

Figure6B+C

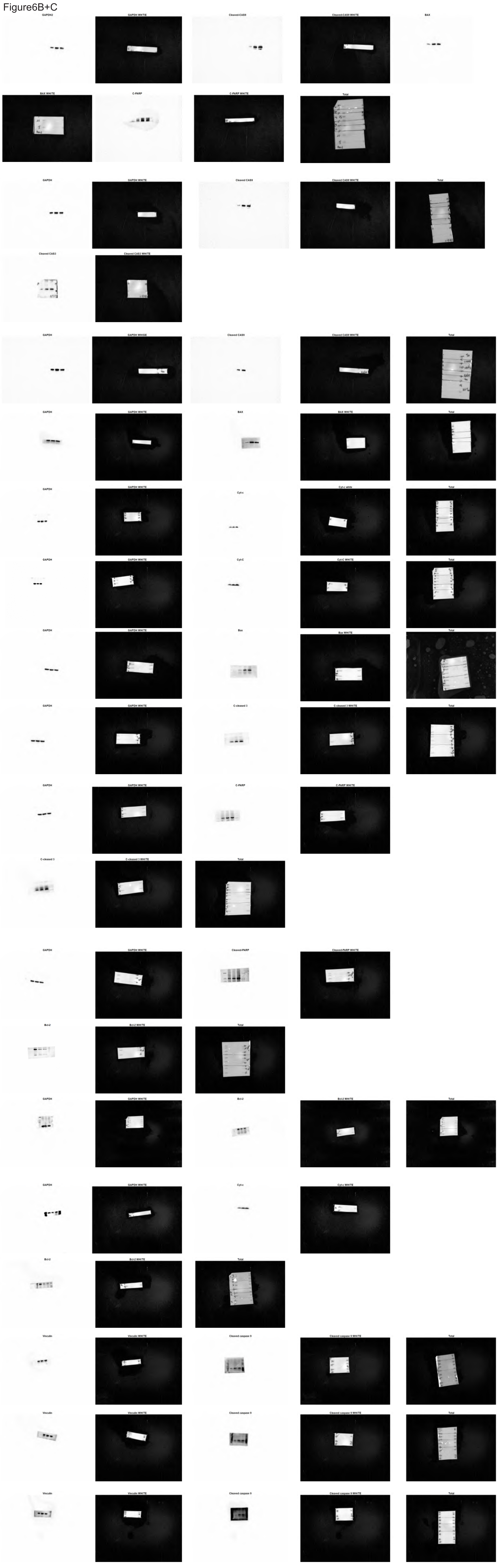

Figure6D

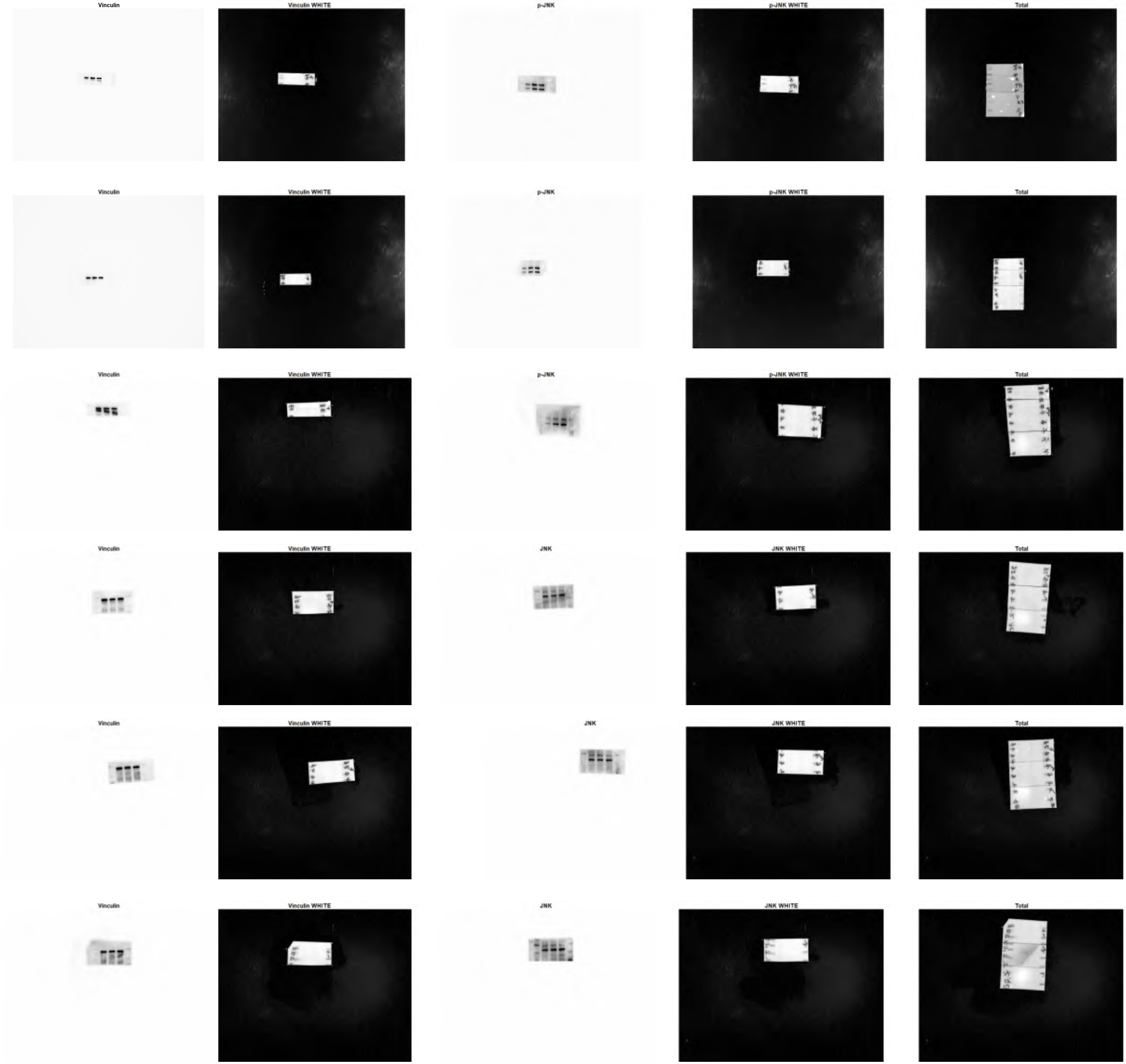
Vinculin
Vinculin WHITE
p-JNK
p-JNK WHITE
Total
Vinculin
Vinculin WHITE
p-JNK
p-JNK WHITE
Total
Vinculin
Vinculin WHITE
p-JNK
p-JNK WHITE
Total
Vinculin
Vinculin WHITE
JNK
JNK WHITE
Total
Vinculin
Vinculin WHITE
JNK
JNK WHITE
Total
Vinculin
Vinculin WHITE
JNK
JNK WHITE
Total

Figure6F+G

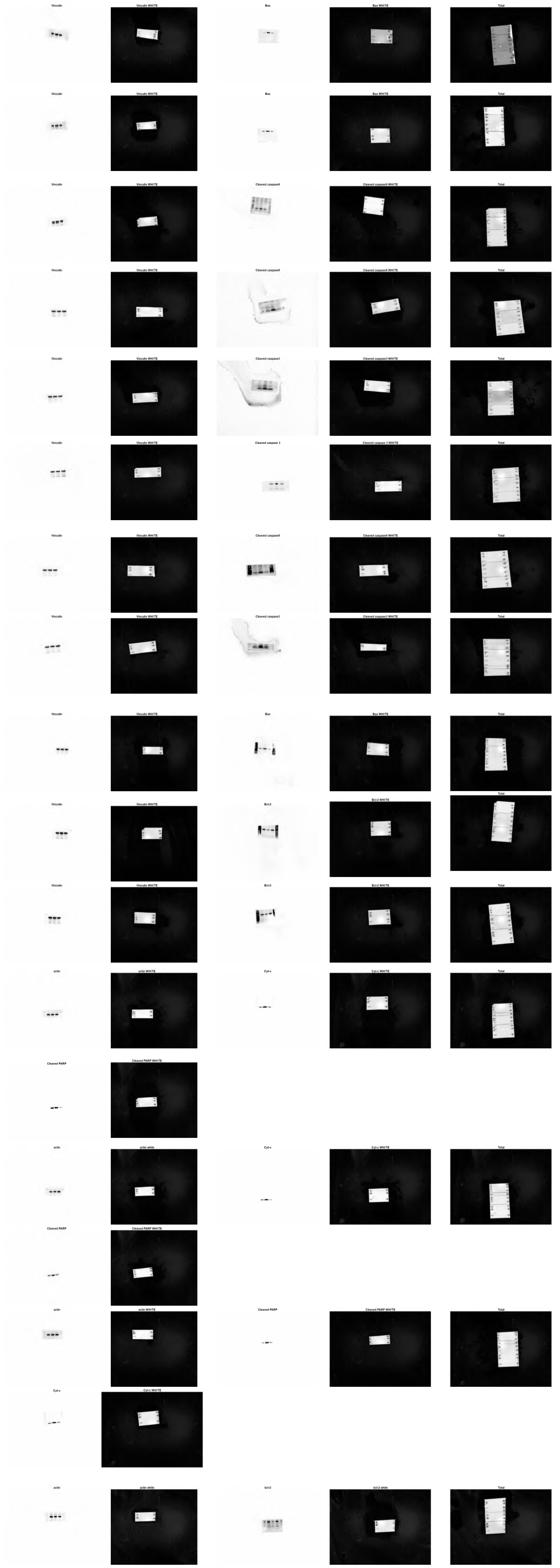

Vinculin
Vinculin WHITE
Bax
Bax WHITE
Total
Vinculin
Vinculin WHITE
Bax
Bax WHITE
Total
Vinculin
Vinculin WHITE
Cleaved caspase9
Cleaved caspase9 WHITE
Total
Vinculin
Vinculin WHITE
Cleaved caspase9
Cleaved caspase9 WHITE
Total
Vinculin
Vinculin WHITE
Cleaved caspase3
Cleaved caspase3 WHITE
Total
Vinculin
Vinculin WHITE
Cleaved caspase 3
Cleaved caspase 3 WHITE
Total
Vinculin
Vinculin WHITE
Cleaved caspase9
Cleaved caspase9 WHITE
Total
Vinculin
Vinculin WHITE
Cleaved caspase3
Cleaved caspase3 WHITE
Total
Vinculin
Vinculin WHITE
Bax
Bax WHITE
Total
Vinculin
Vinculin WHITE
Bcl-2
Bcl-2 WHITE
Total
Vinculin
Vinculin WHITE
Bcl-2
Bcl-2 WHITE
Total
actin
actin WHITE
Cyt-c
Cyt-c WHITE
Total
Cleaved PARP
Cleaved PARP WHITE
actin
actin white
Cyt-c
Cyt-c WHITE
Total
Cleaved PARP
Cleaved PARP WHITE
actin
actin WHITE
Cleaved PARP
Cleaved PARP WHITE
Total
Cyt-c
Cyt-c WHITE
actin
actin white
bcl-2
bcl-2 white
Total

Figure6H

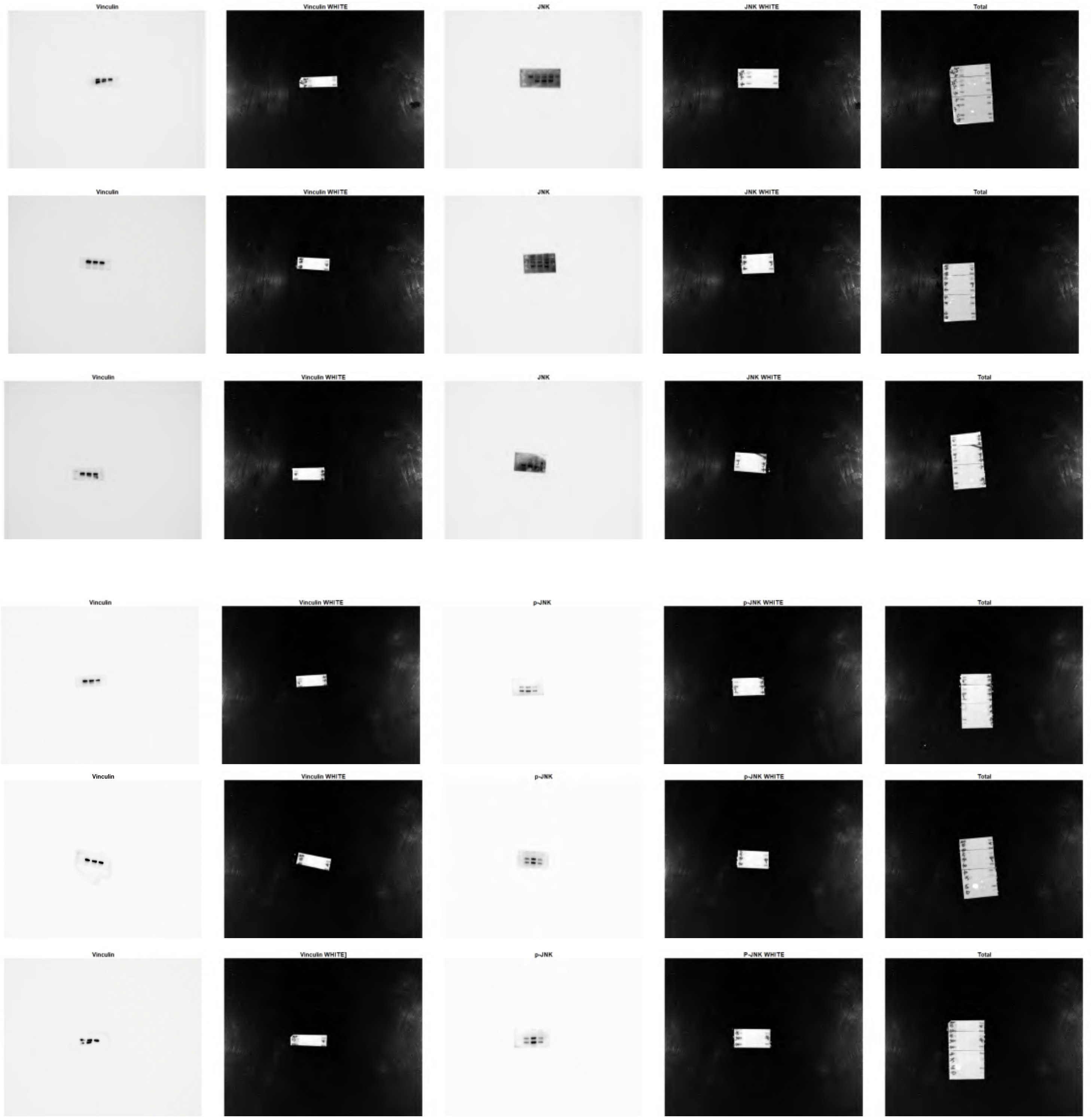
Vinculin
Vinculin WHITE
JNK
JNK WHITE
Total
Vinculin
Vinculin WHITE
JNK
JNK WHITE
Total
Vinculin
Vinculin WHITE
JNK
JNK WHITE
Total
Vinculin
Vinculin WHITE
p-JNK
p-JNK WHITE
Total
Vinculin
Vinculin WHITE
p-JNK
p-JNK WHITE
Total
Vinculin
Vinculin WHITE]
p-JNK
P-JNK WHITE
Total

Figure7B

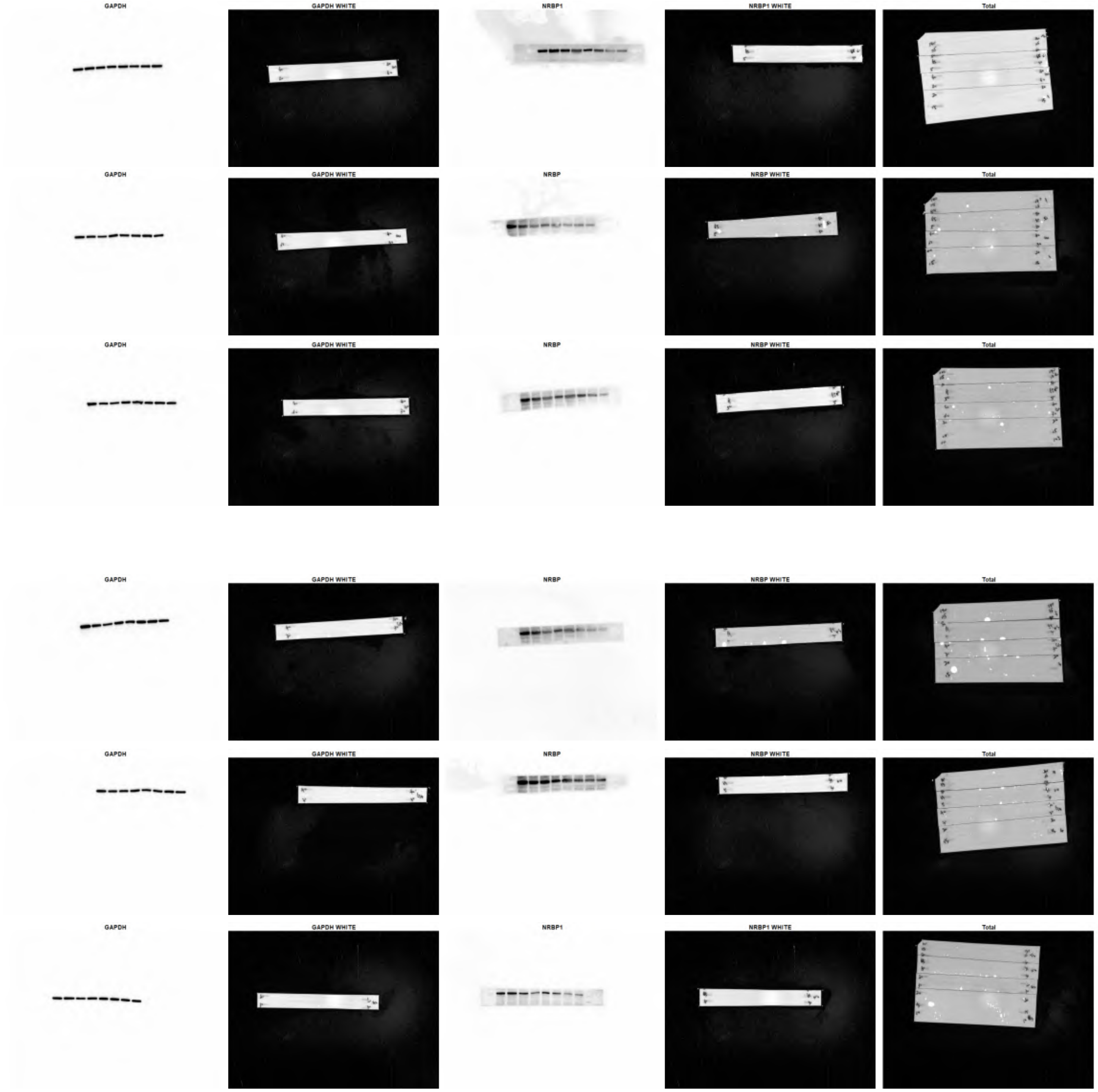
GAPDH
GAPDH WHITE
NRBP1
NRBP1 WHITE
Total
GAPDH
GAPDH WHITE
NRBP
NRBP WHITE
Total
GAPDH
GAPDH WHITE
NRBP
NRBP WHITE
Total
GAPDH
GAPDH WHITE
NRBP
NRBP WHITE
Total
GAPDH
GAPDH WHITE
NRBP
NRBP WHITE
Total
GAPDH
GAPDH WHITE
NRBP1
NRBP1 WHITE
Total

Figure 7C

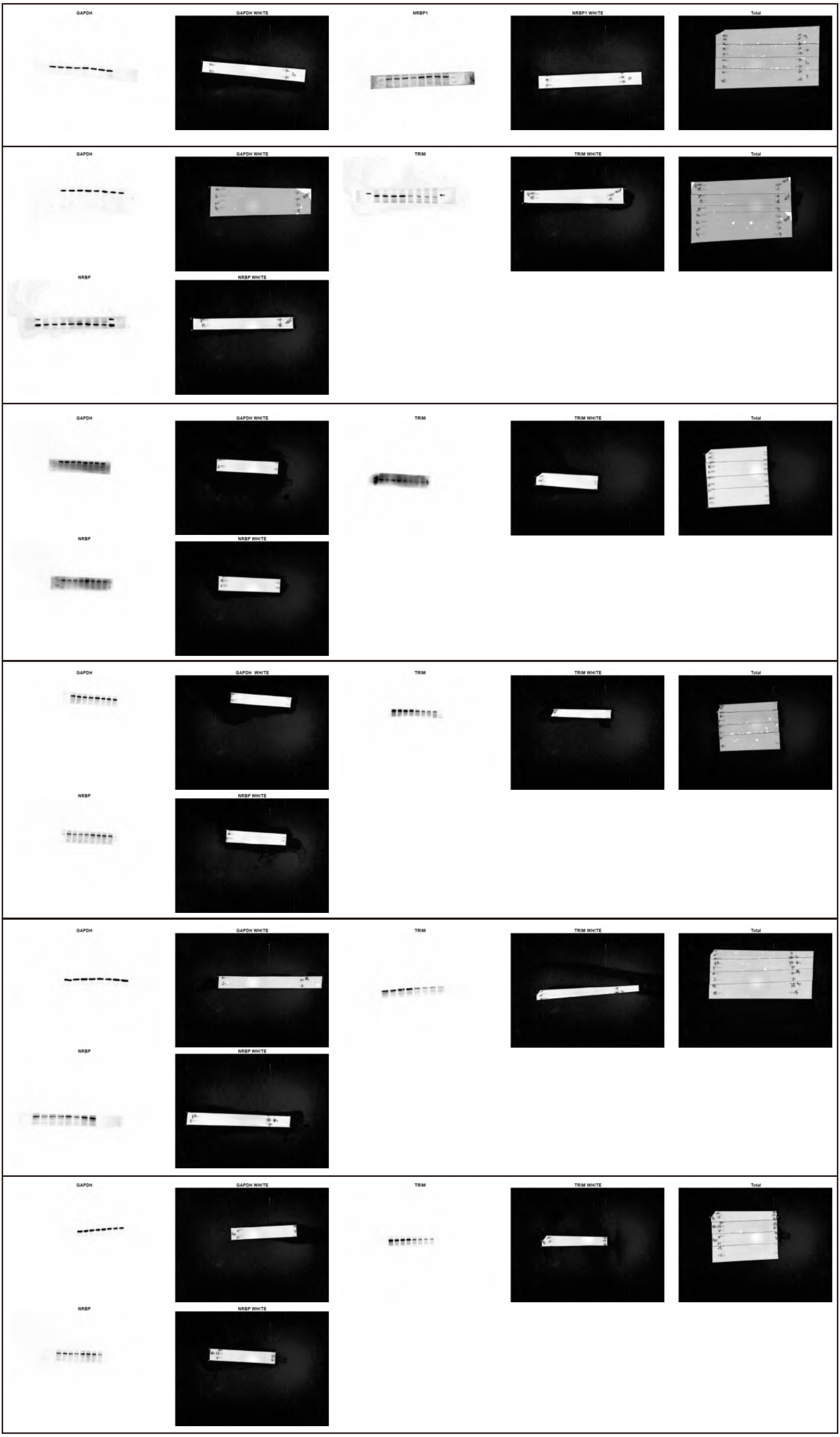
GAPDH
GAPDH WHITE
NRBP1
NRBP1 WHITE
Total
GAPDH
GAPDH WHITE
TRIM
TRIM WHITE
Total
NRBP
NRBP WHITE
GAPDH
GAPDH WHITE
TRIM
TRIM WHITE
Total
NRBP
NRBP WHITE
GAPDH
GAPDH WHITE
TRIM
TRIM WHITE
Total
NRBP
NRBP WHITE
GAPDH
GAPDH WHITE
TRIM
TRIM WHITE
Total
NRBP
NRBP WHITE
GAPDH
GAPDH WHITE
TRIM
TRIM WHITE
Total
NRBP
NRBP WHITE

Figure 7D

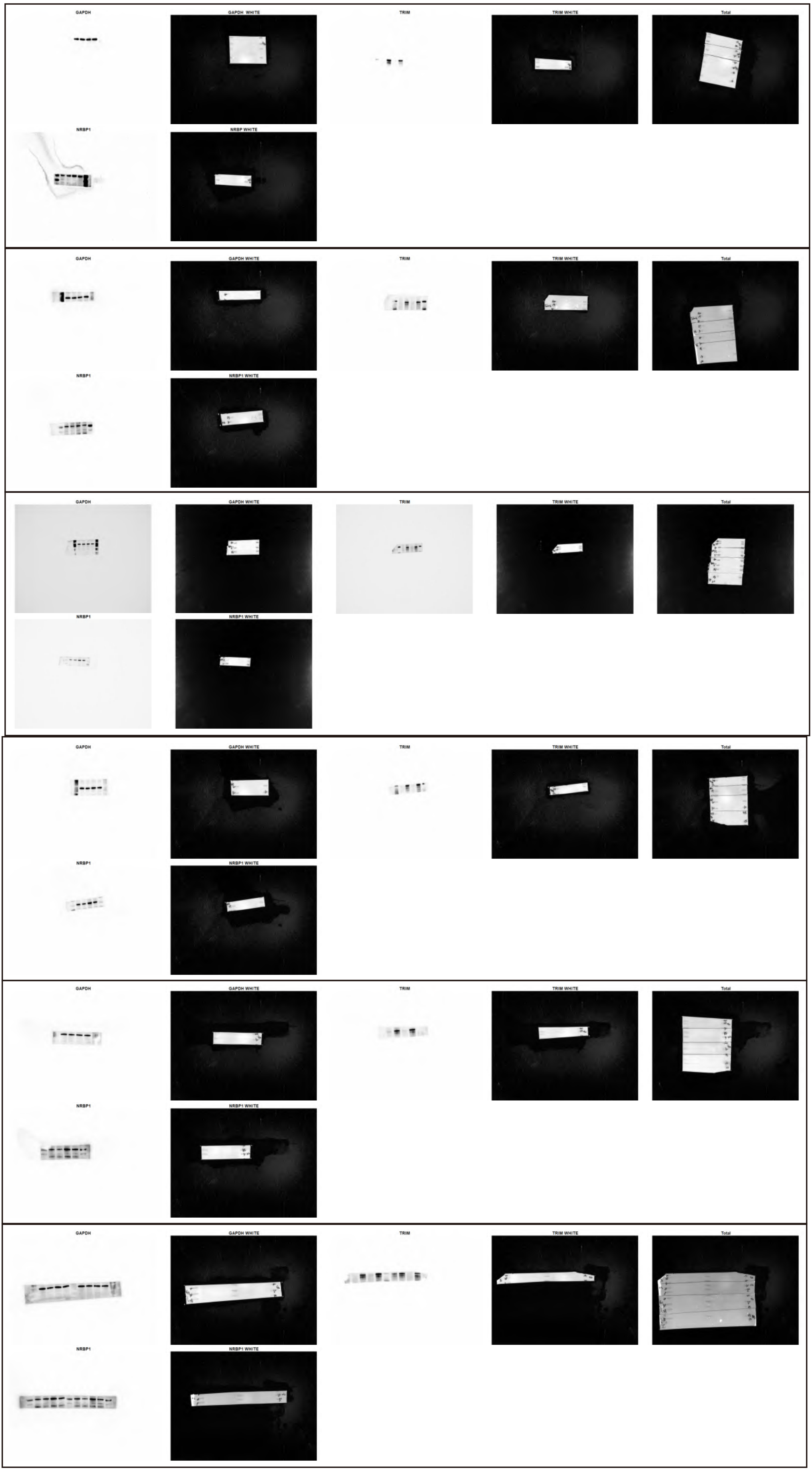
GAPDH
GAPDH WHITE
TRIM
TRIM WHITE
Total
NRBP1
NRBP WHITE
GAPDH
GAPDH WHITE
TRIM
TRIM WHITE
Total
NRBP1
NRBP1 WHITE
GAPDH
GAPDH WHITE
TRIM
TRIM WHITE
Total
NRBP1
NRBP1 WHITE
GAPDH
GAPDH WHITE
TRIM
TRIM WHITE
Total
NRBP1
NRBP1 WHITE
GAPDH
GAPDH WHITE
TRIM
TRIM WHITE
Total
NRBP1
NRBP1 WHITE
GAPDH
GAPDH WHITE
TRIM
TRIM WHITE
Total
NRBP1
NRBP1 WHITE

NRBP1

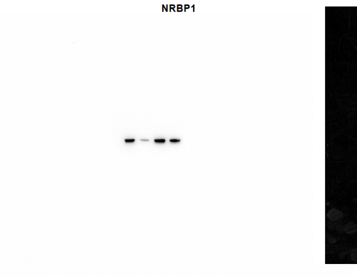

NRBP1 WHITE

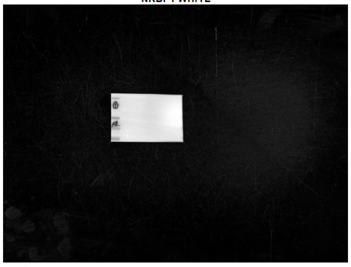

GAPDH

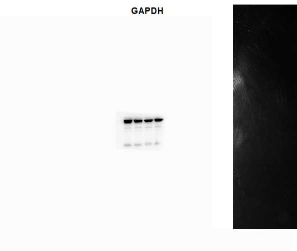

GAPDH WHITE

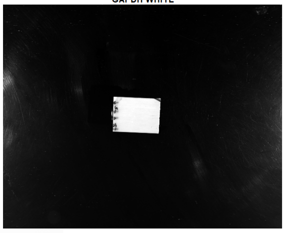

Total

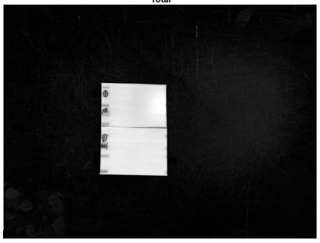

Figure 7E

TRIM

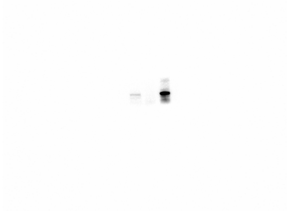

TRIM WHITE

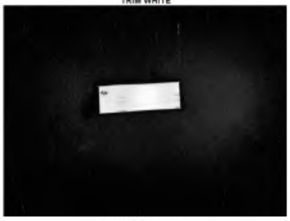

NRBP

NRBP WHITE

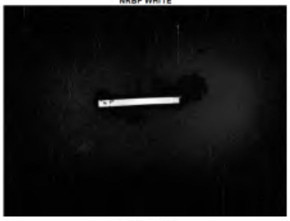

Total

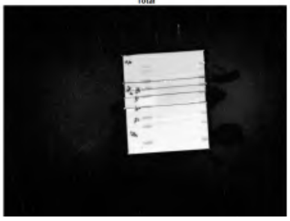

TRIM24

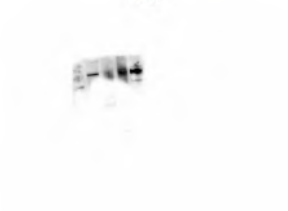

TRIM24 WHITE

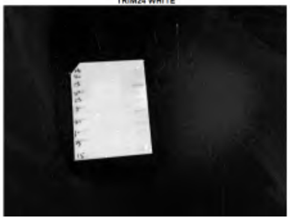

NRBP1

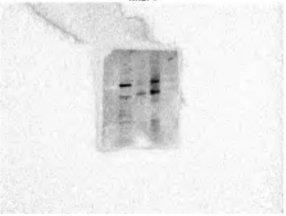

NRBP1 WHITE

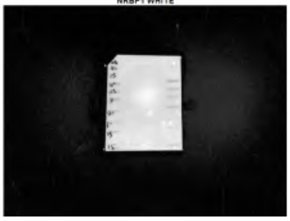

TRIM24

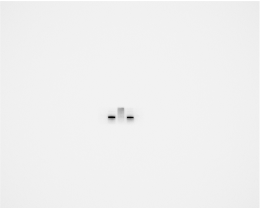

TRIM24 WHITE

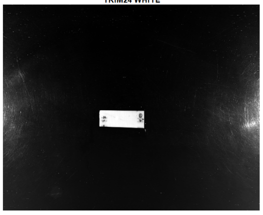

NRBP1 WHITE

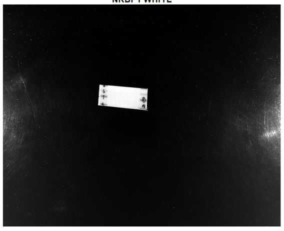

NRBP1

Total

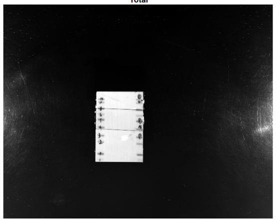

TRIM24

TRIM24 WHITE

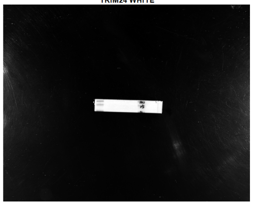

NRBP

NRBP WHITE

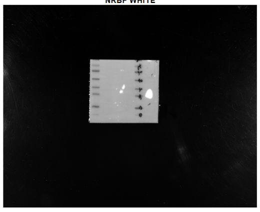

Total

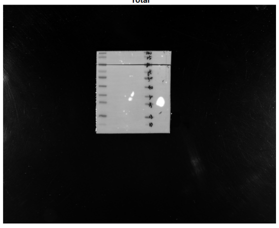

Figure 7F

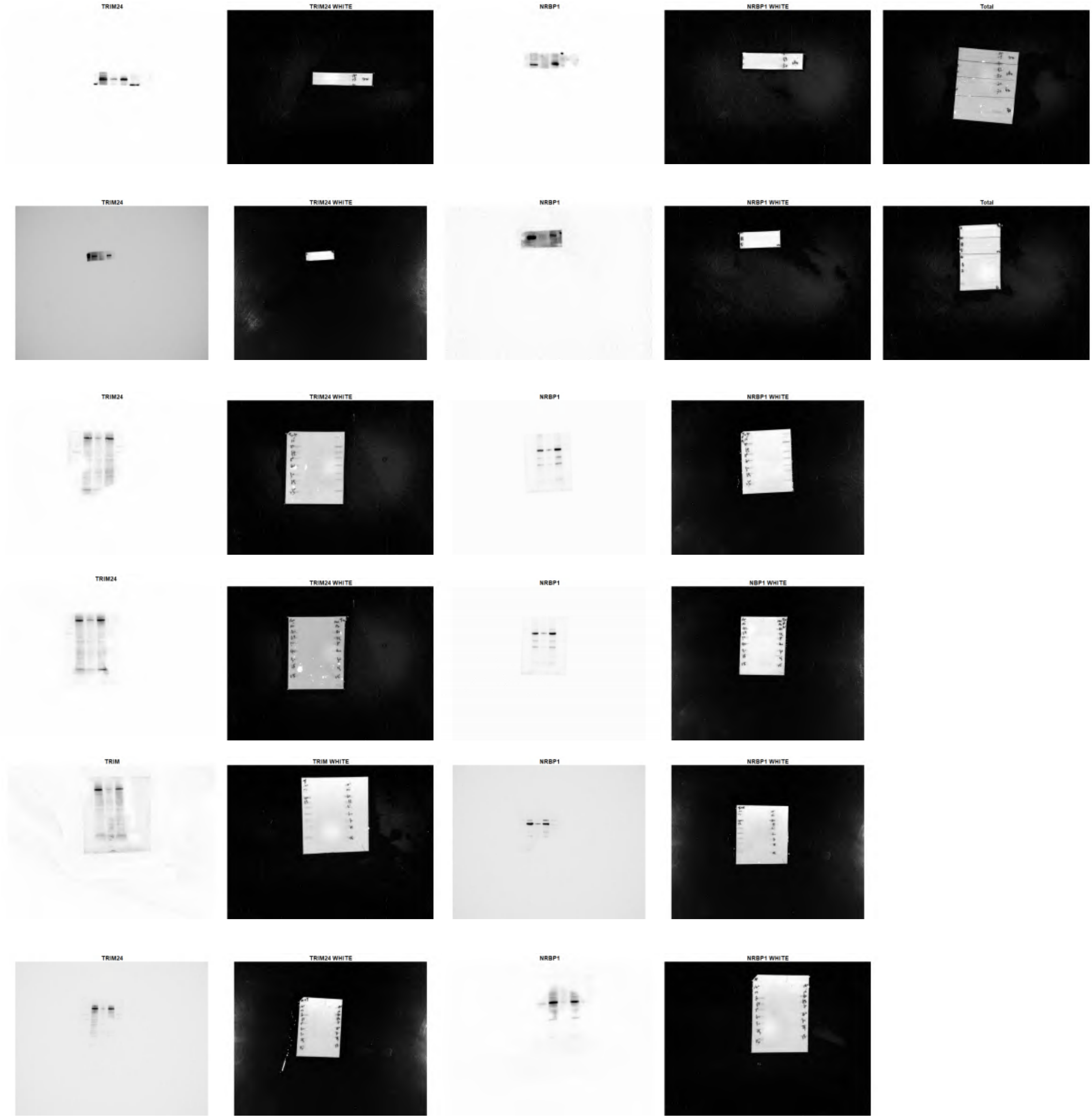
TRIM24
TRIM24 WHITE
NRBP1
NRBP1 WHITE
Total
TRIM24
TRIM24 WHITE
NRBP1
NRBP1 WHITE
Total
TRIM24
TRIM24 WHITE
NRBP1
NRBP1 WHITE
TRIM24
TRIM24 WHITE
NRBP1
NBP1 WHITE
TRIM
TRIM WHITE
NRBP1
NRBP1 WHITE
TRIM24
TRIM24 WHITE
NRBP1
NRBP1 WHITE

Figure7 G/H/I

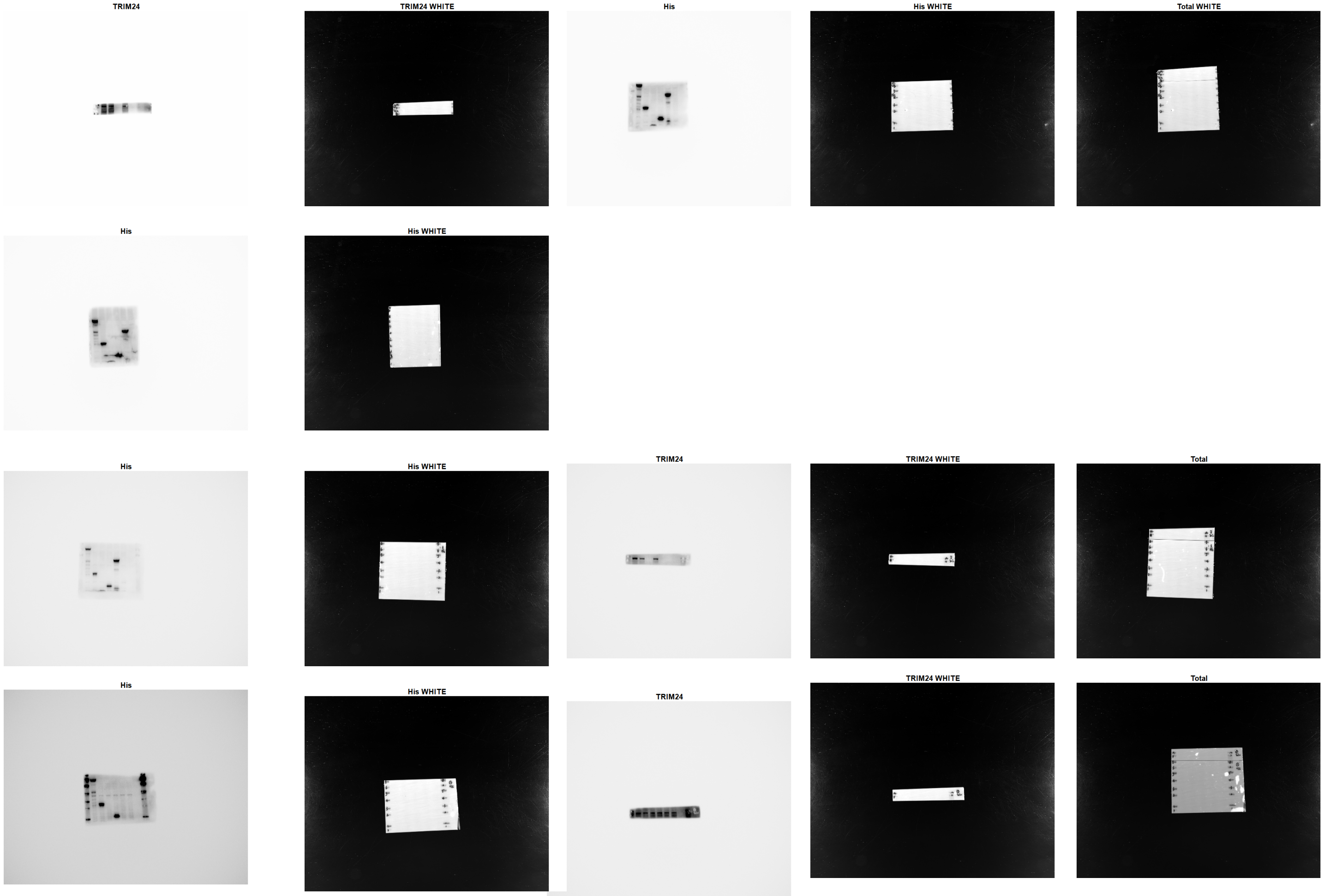
TRIM24
TRIM24 WHITE
His
His WHITE
Total WHITE
His
His WHITE
His
His WHITE
TRIM24
TRIM24 WHITE
Total
His
His WHITE
TRIM24
TRIM24 WHITE
Total

Figure 7J

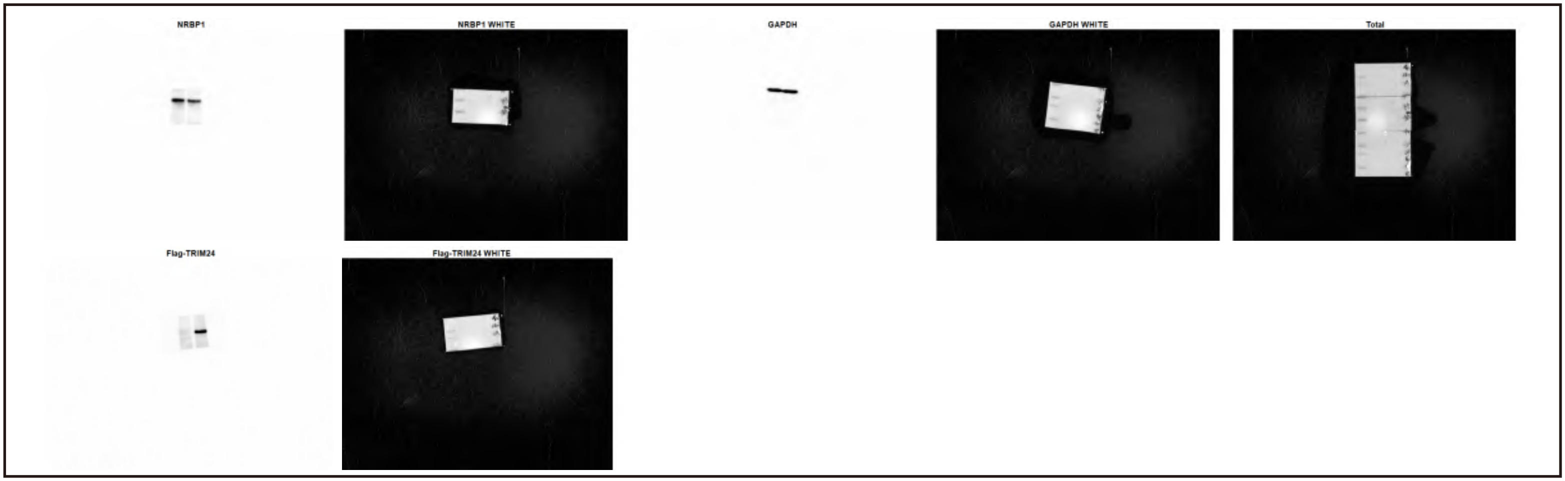
NRBP1
NRBP1 WHITE
GAPDH
GAPDH WHITE
Total
Flag-TRIM24
Flag-TRIM24 WHITE

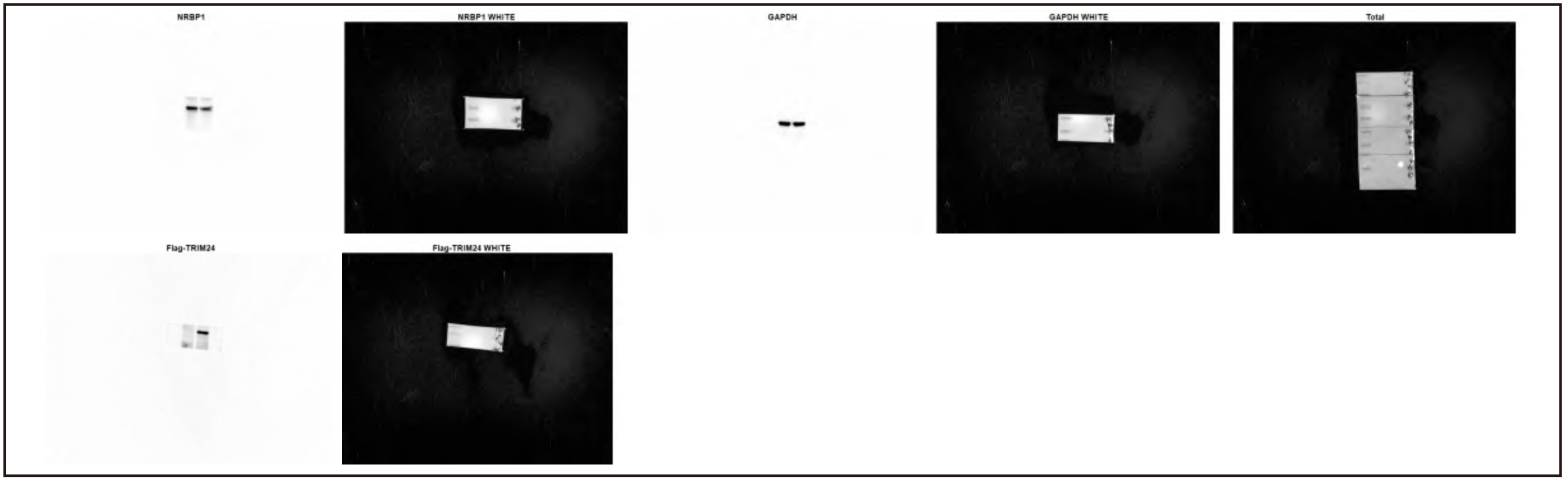
NRBP1
NRBP1 WHITE
GAPDH
GAPDH WHITE
Total
Flag-TRIM24
Flag-TRIM24 WHITE

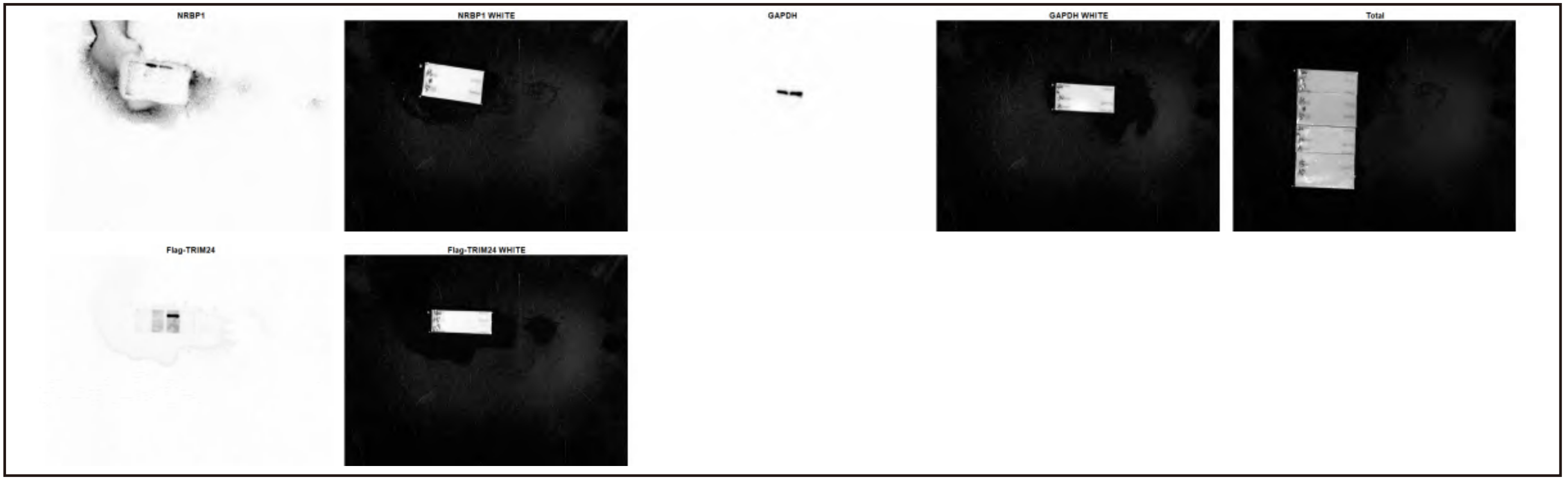
NRBP1
NRBP1 WHITE
GAPDH
GAPDH WHITE
Total
Flag-TRIM24
Flag-TRIM24 WHITE

Figure 7K

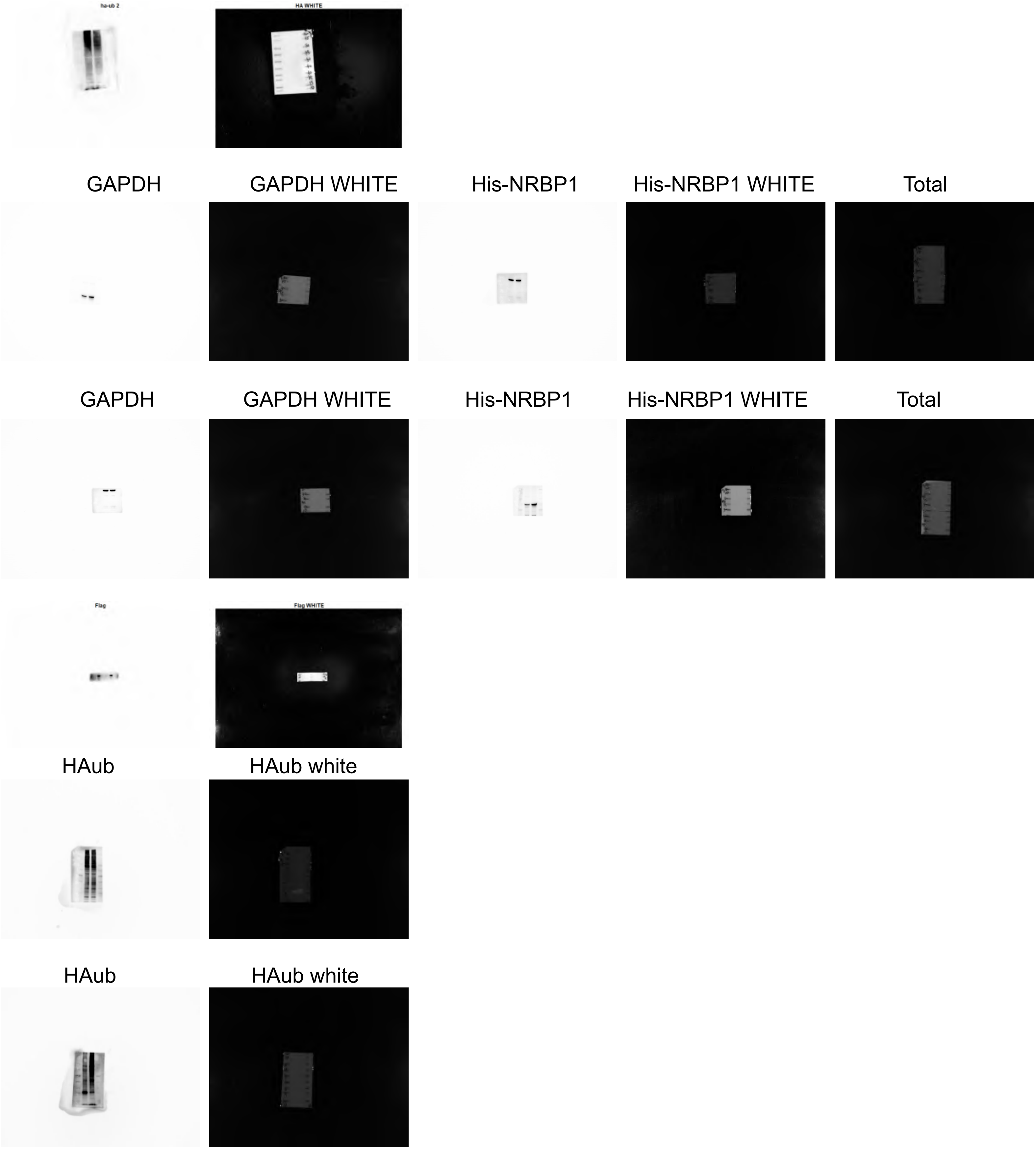
ha-ub 2
HA WHITE
GAPDH
GAPDH WHITE
His-NRBP1
His-NRBP1 WHITE
Total
GAPDH
GAPDH WHITE
His-NRBP1
His-NRBP1 WHITE
Total
Flag
Flag WHITE
HAub
HAub white
HAub
HAub white

Figure 7L

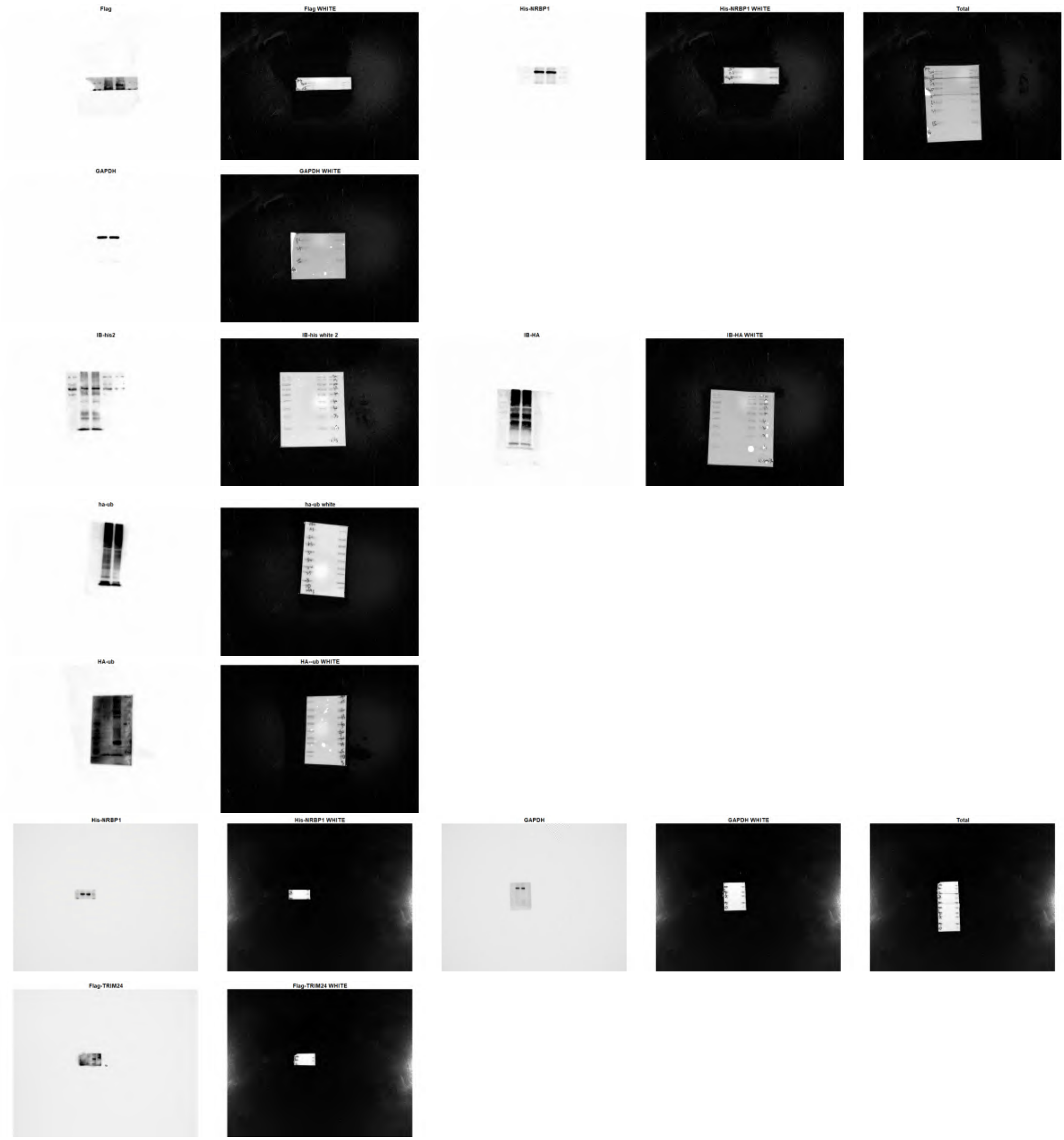
Flag
Flag WHITE
His-NRBP1
His-NRBP1 WHITE
Total
GAPDH
GAPDH WHITE
IB-his2
IB-his white 2
IB-HA
IB-HA WHITE
ha-ub
ha-ub white
HA-ub
HA--ub WHITE
His-NRBP1
His-NRBP1 WHITE
GAPDH
GAPDH WHITE
Total
Flag-TRIM24
Flag-TRIM24 WHITE

Figure8A

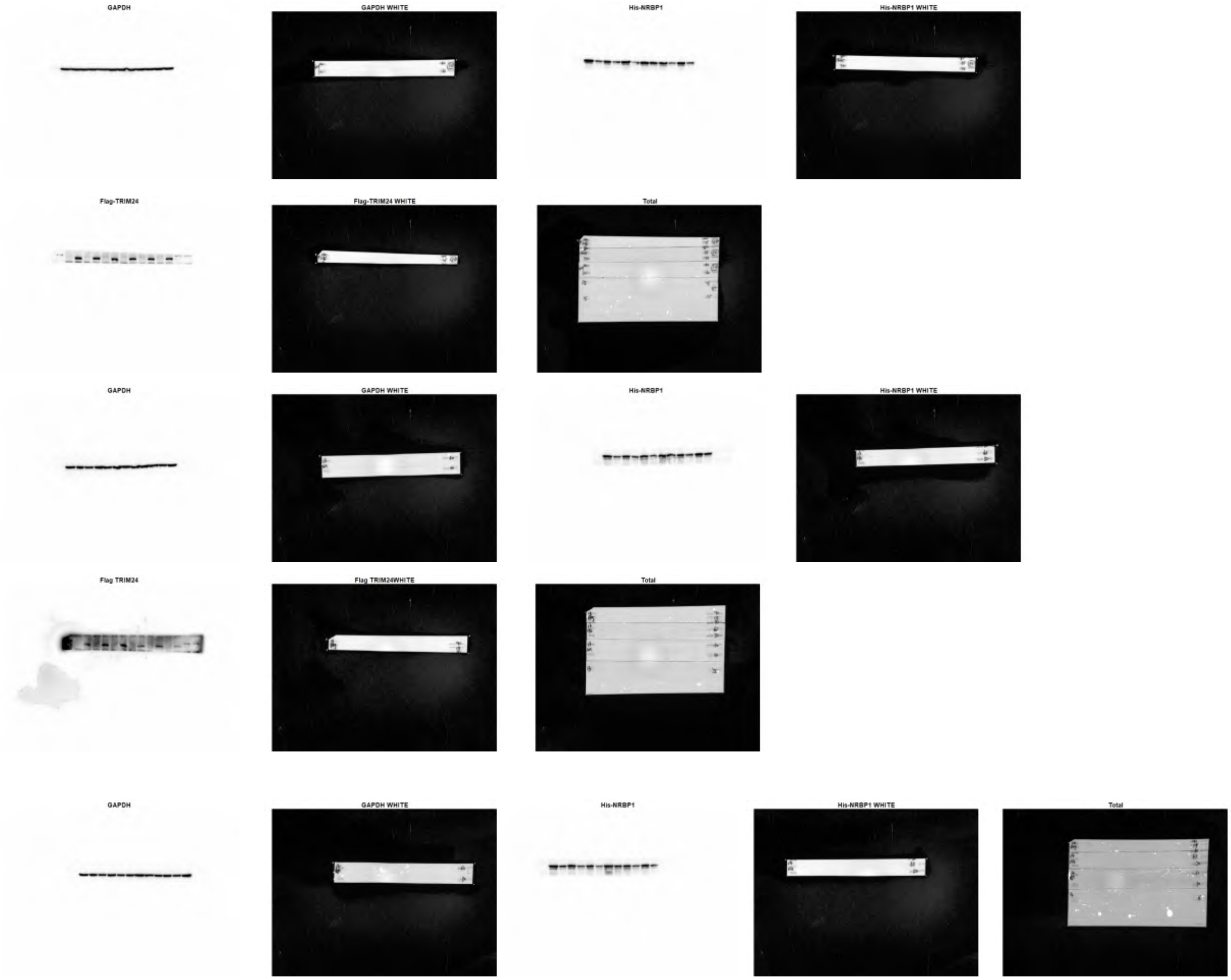

Figure8B

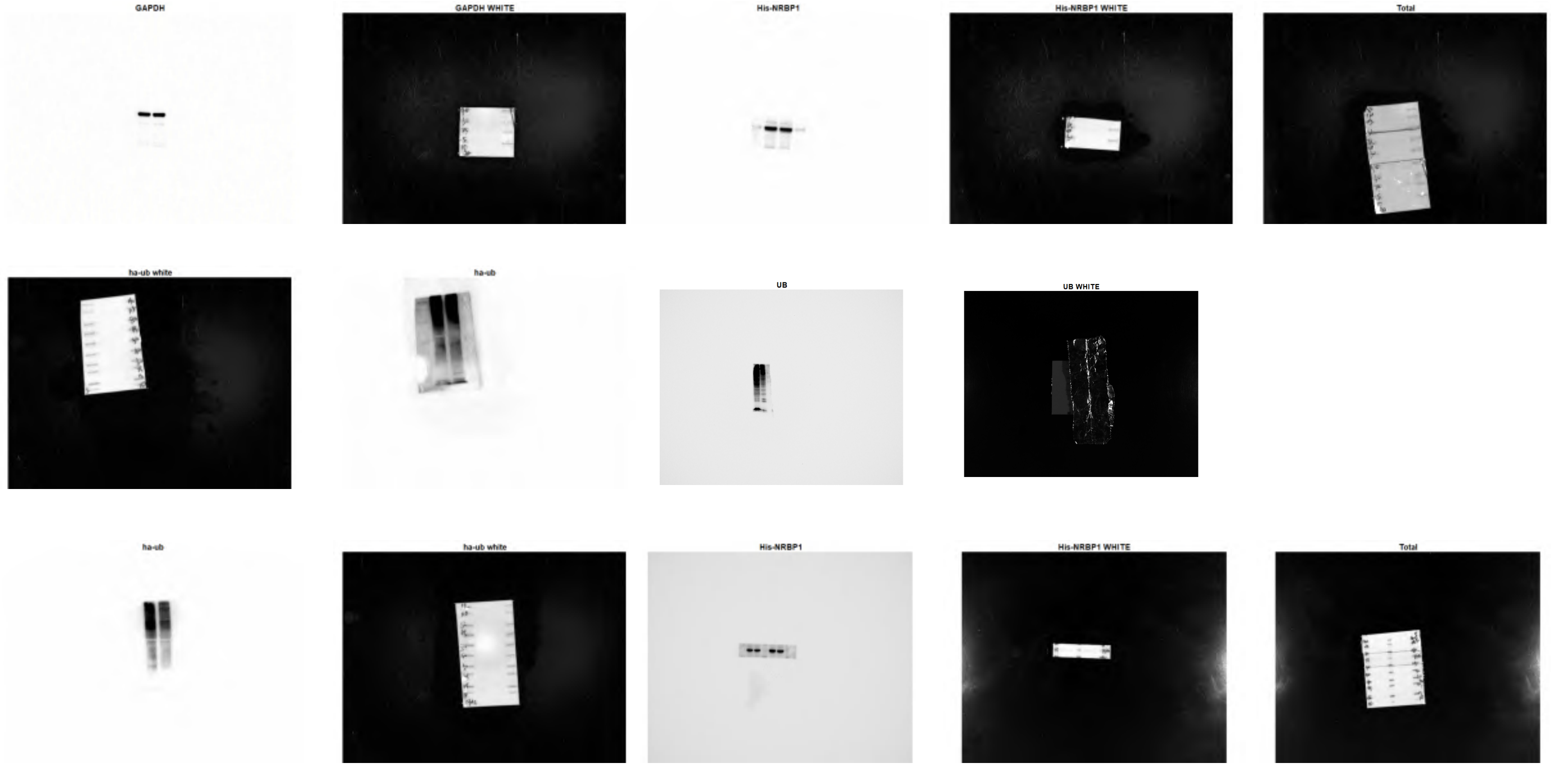

Figure8F

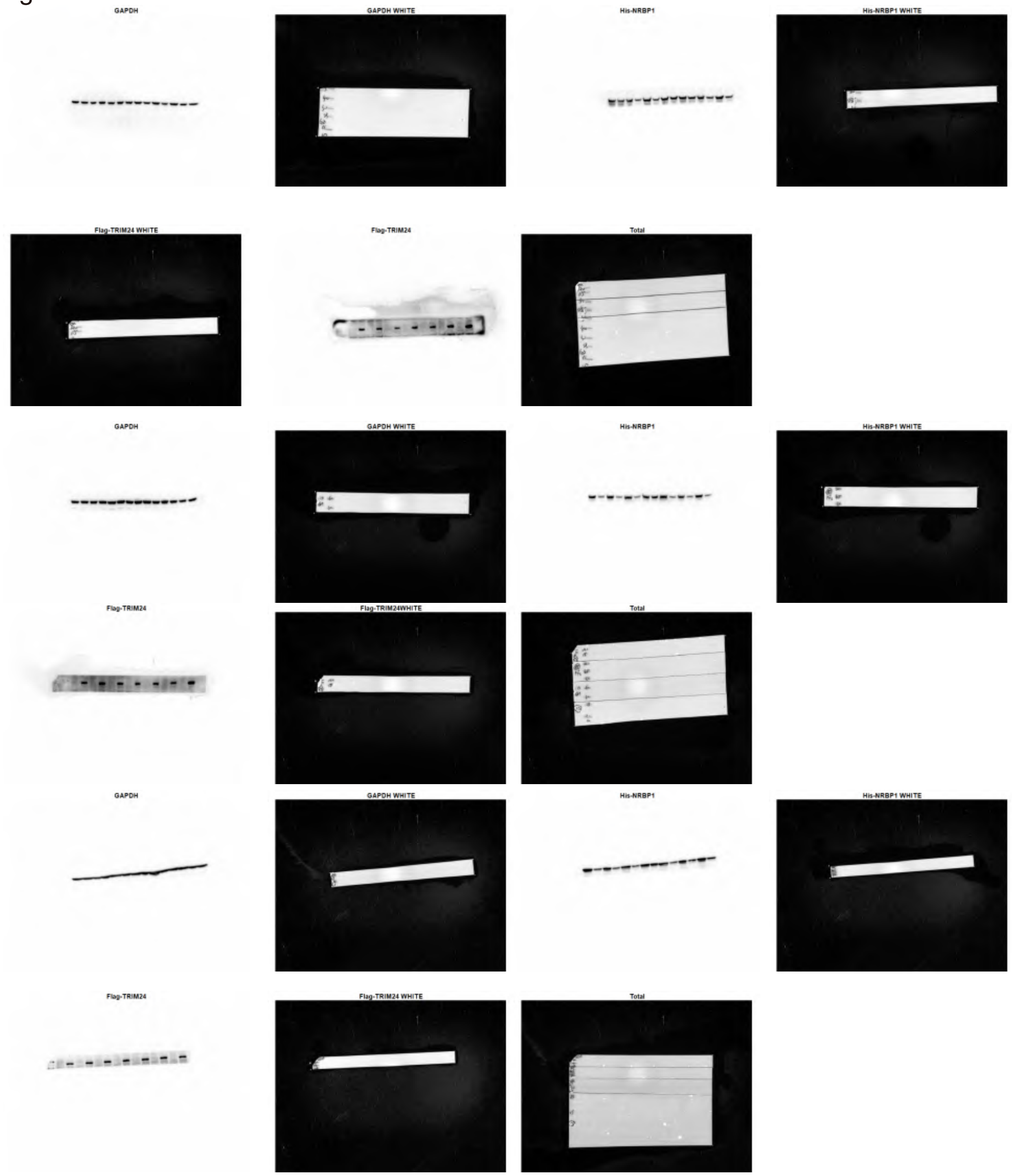

Figure 8G

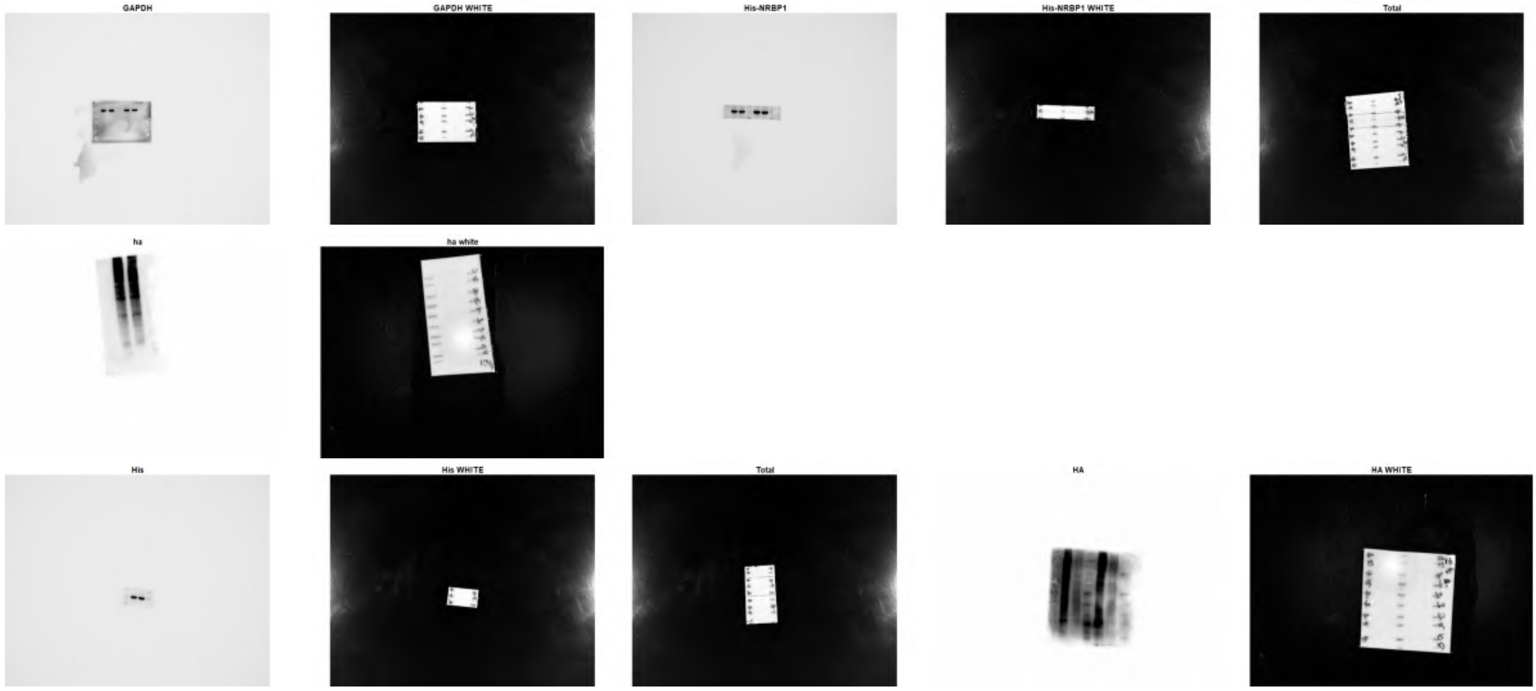

Supplementary Figure 3E

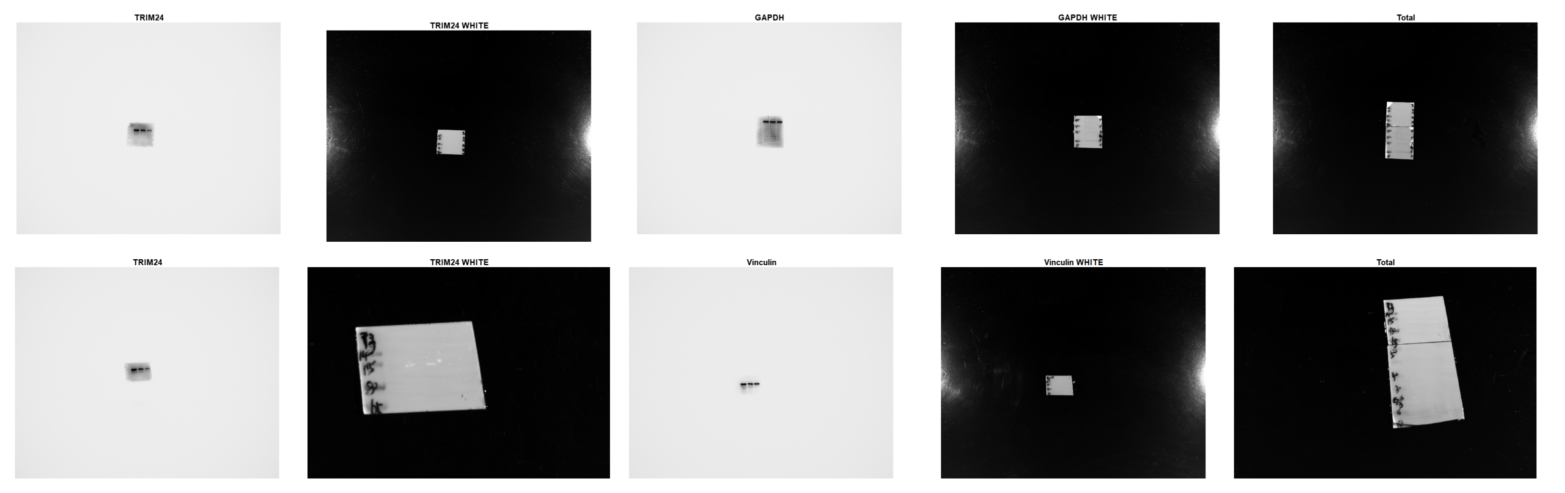

Supplementary Figure 4A

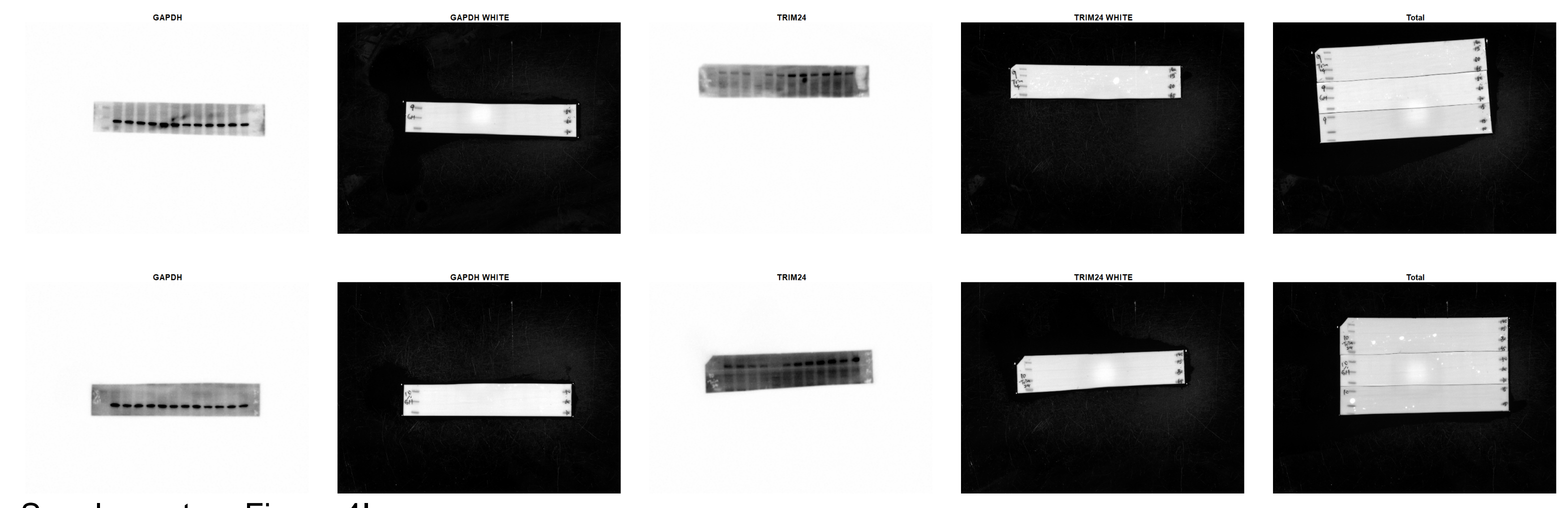

Supplementary Figure 4I

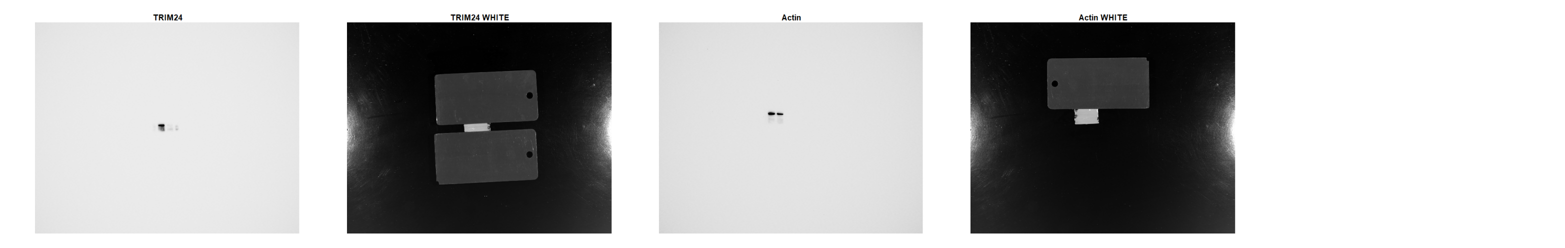

Supplementary Figure 5B

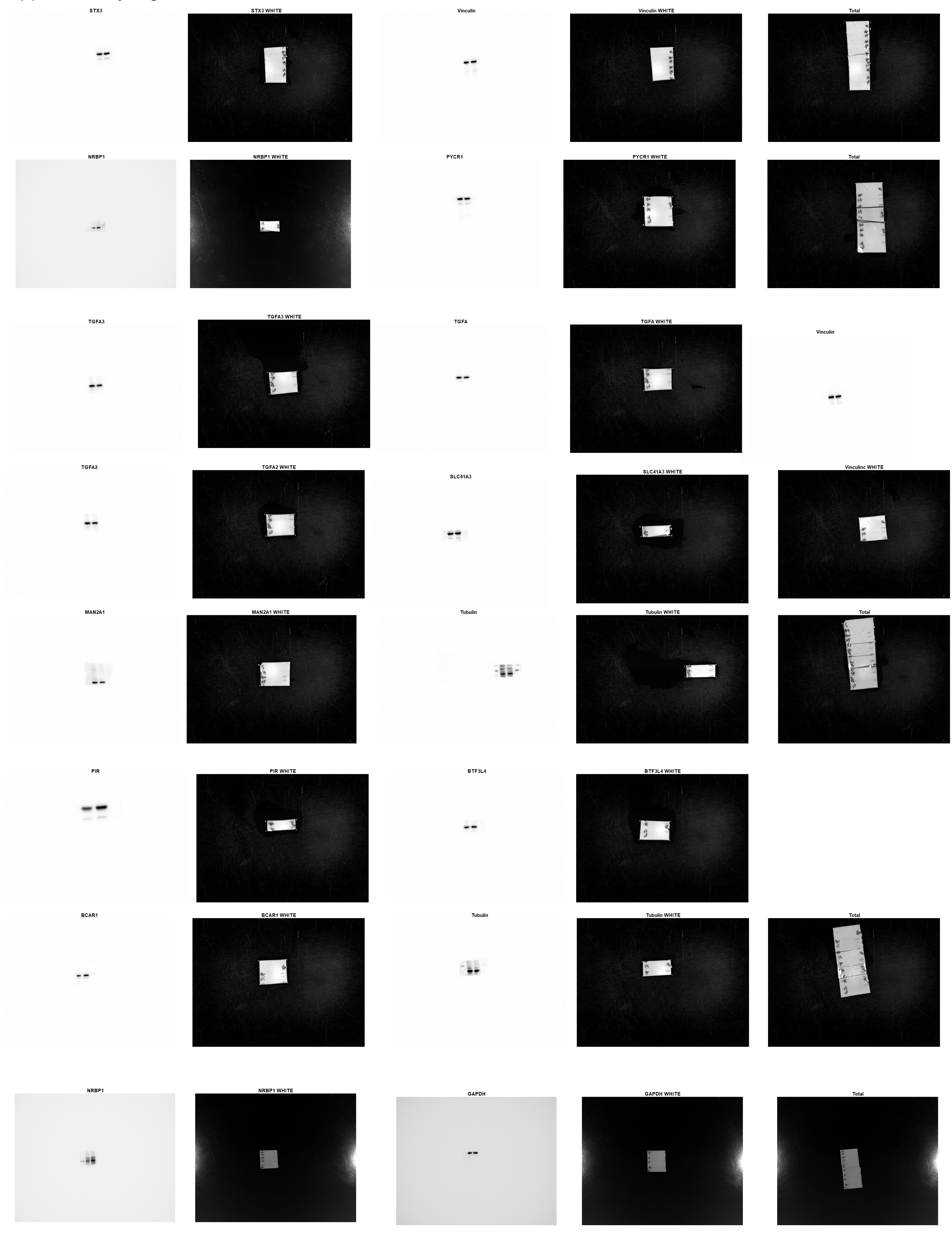

Supplementary Figure 5C

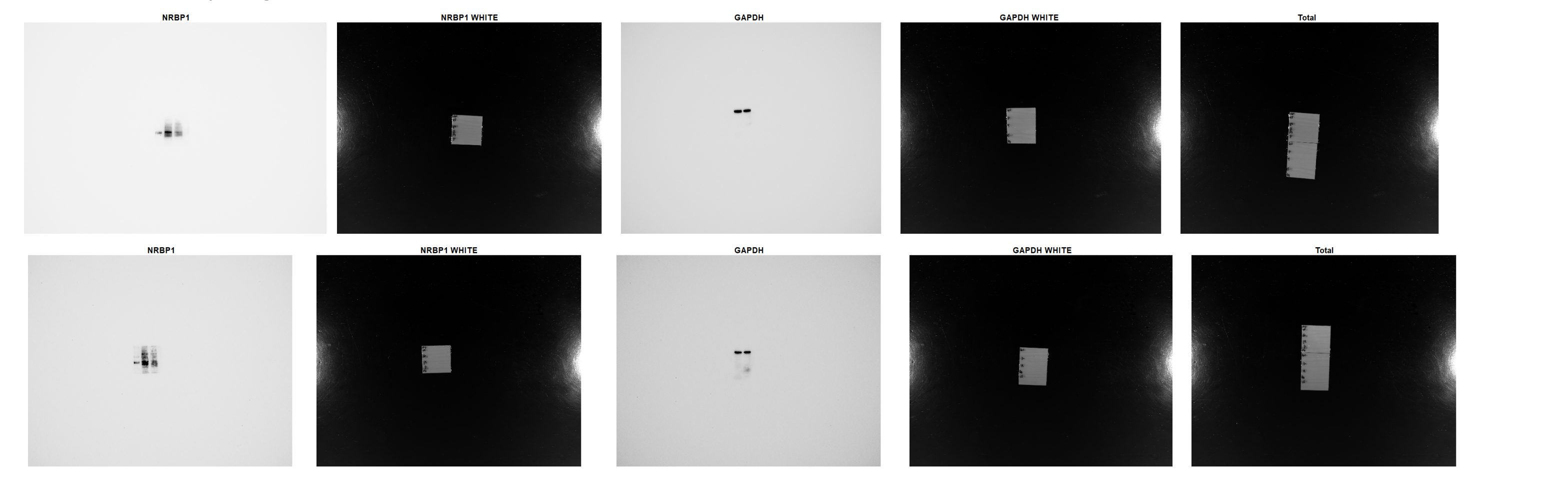

Supplementary Figure 5D

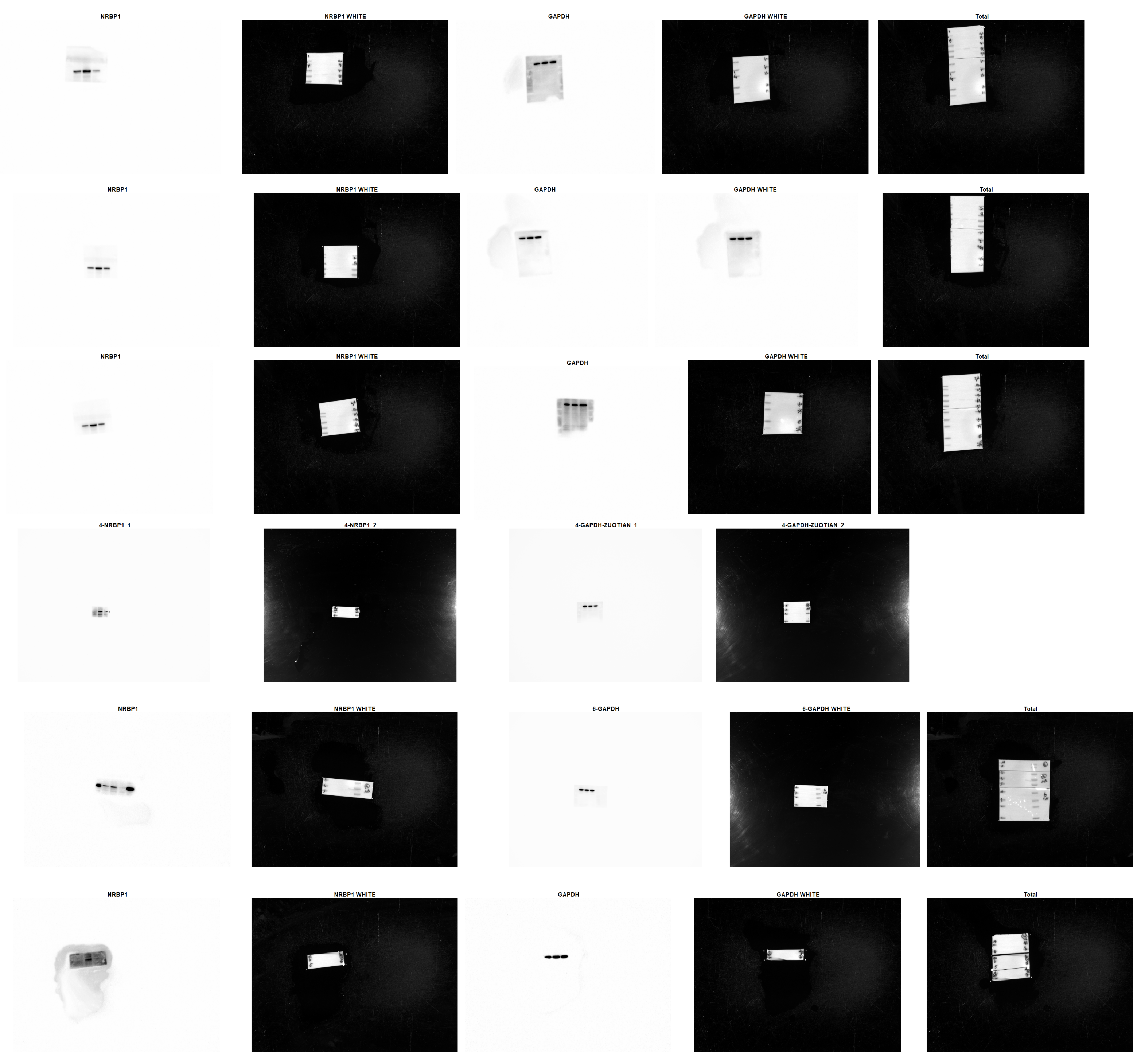

Supplementary Figure 6B+C

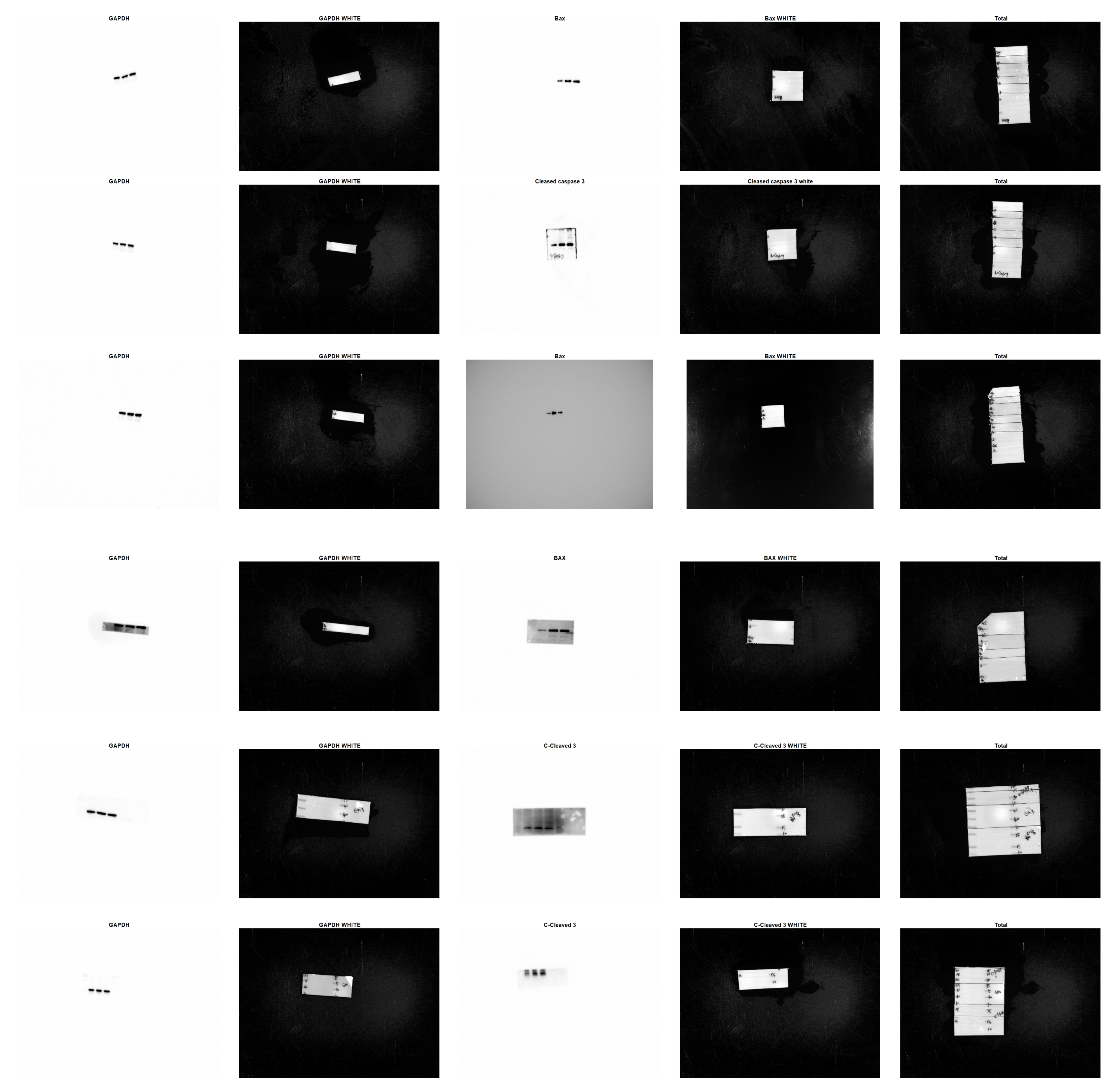

Supplementary Figure 6B+C

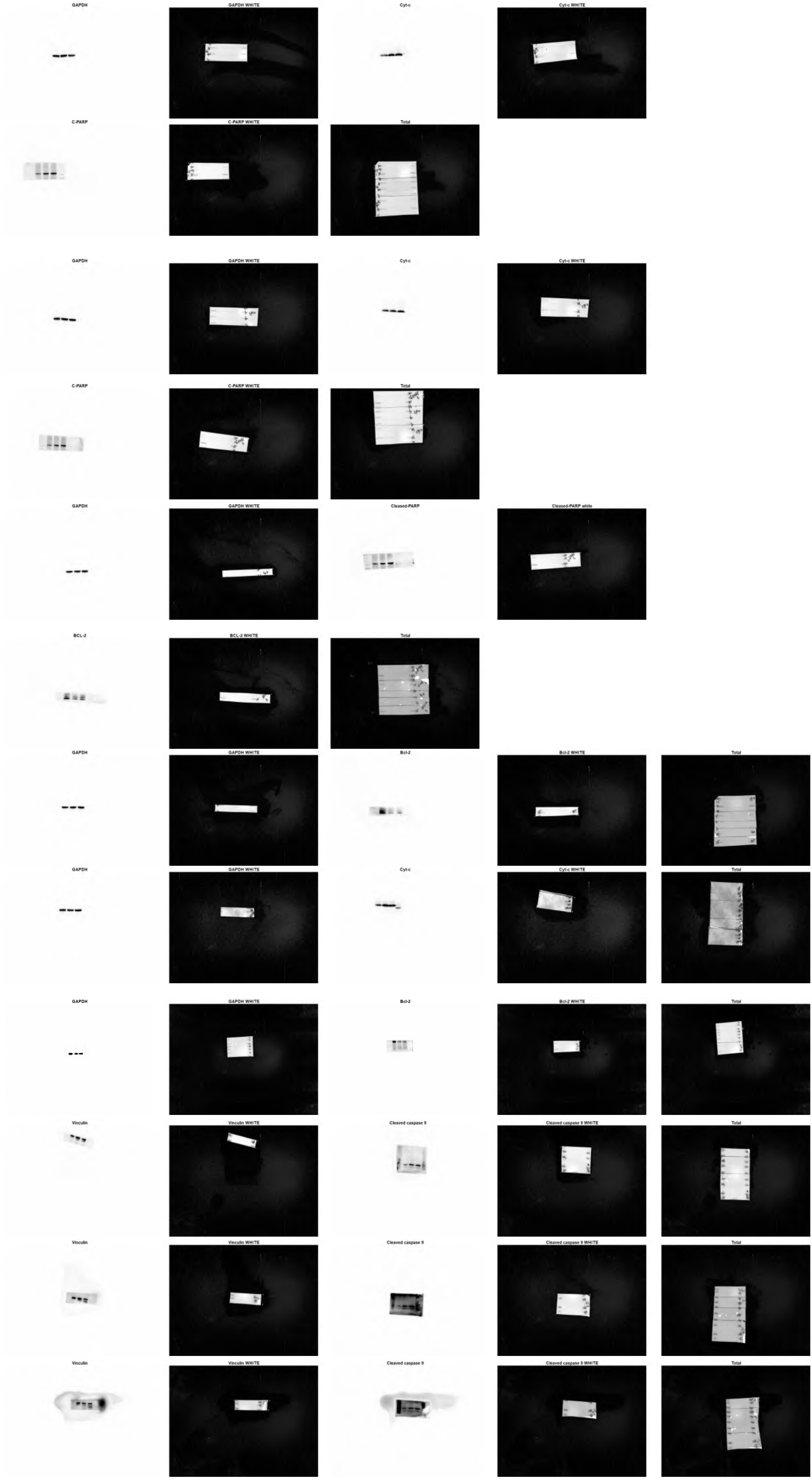

Supplementary Figure 6D

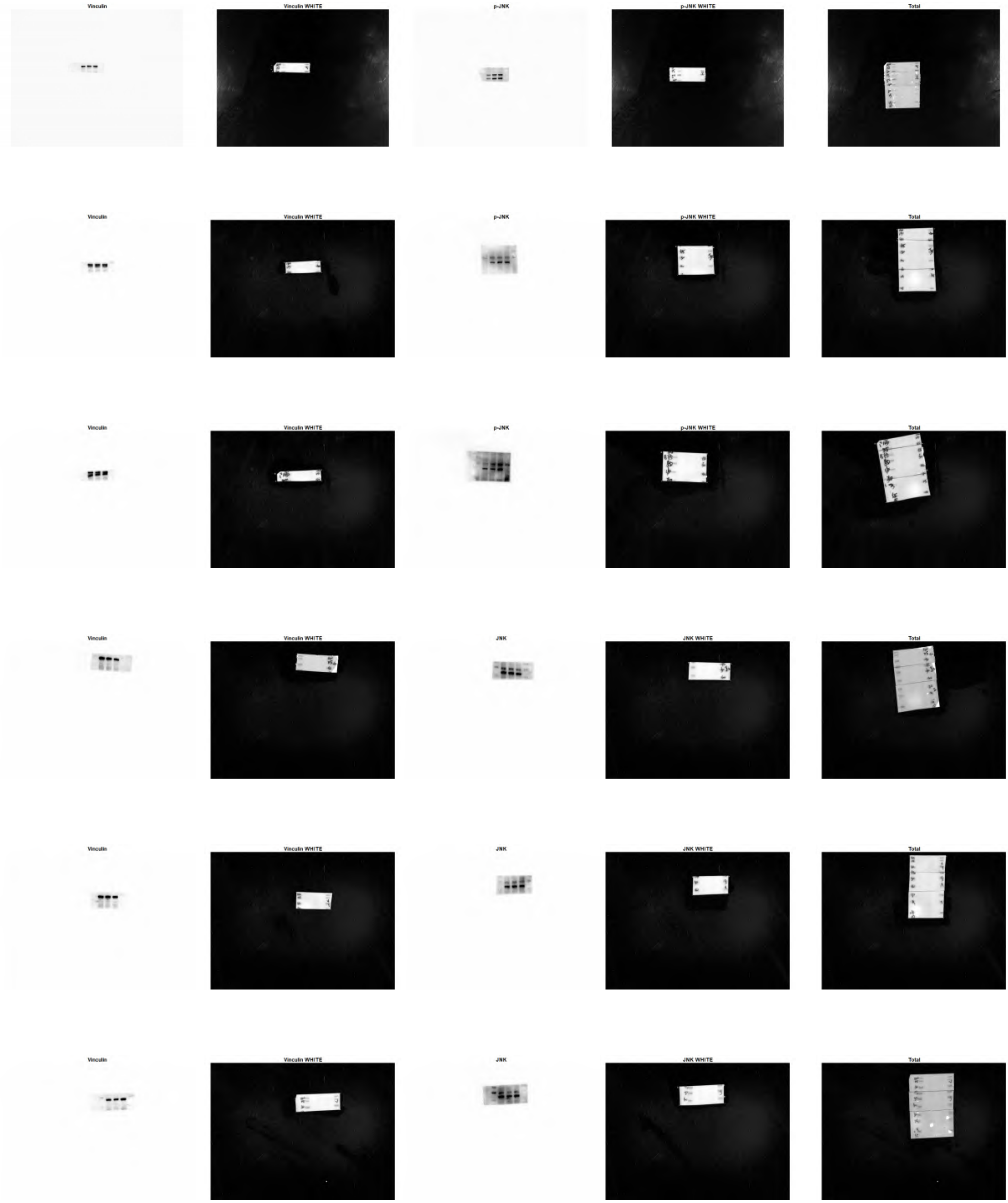

Supplementary Figure 6F+G

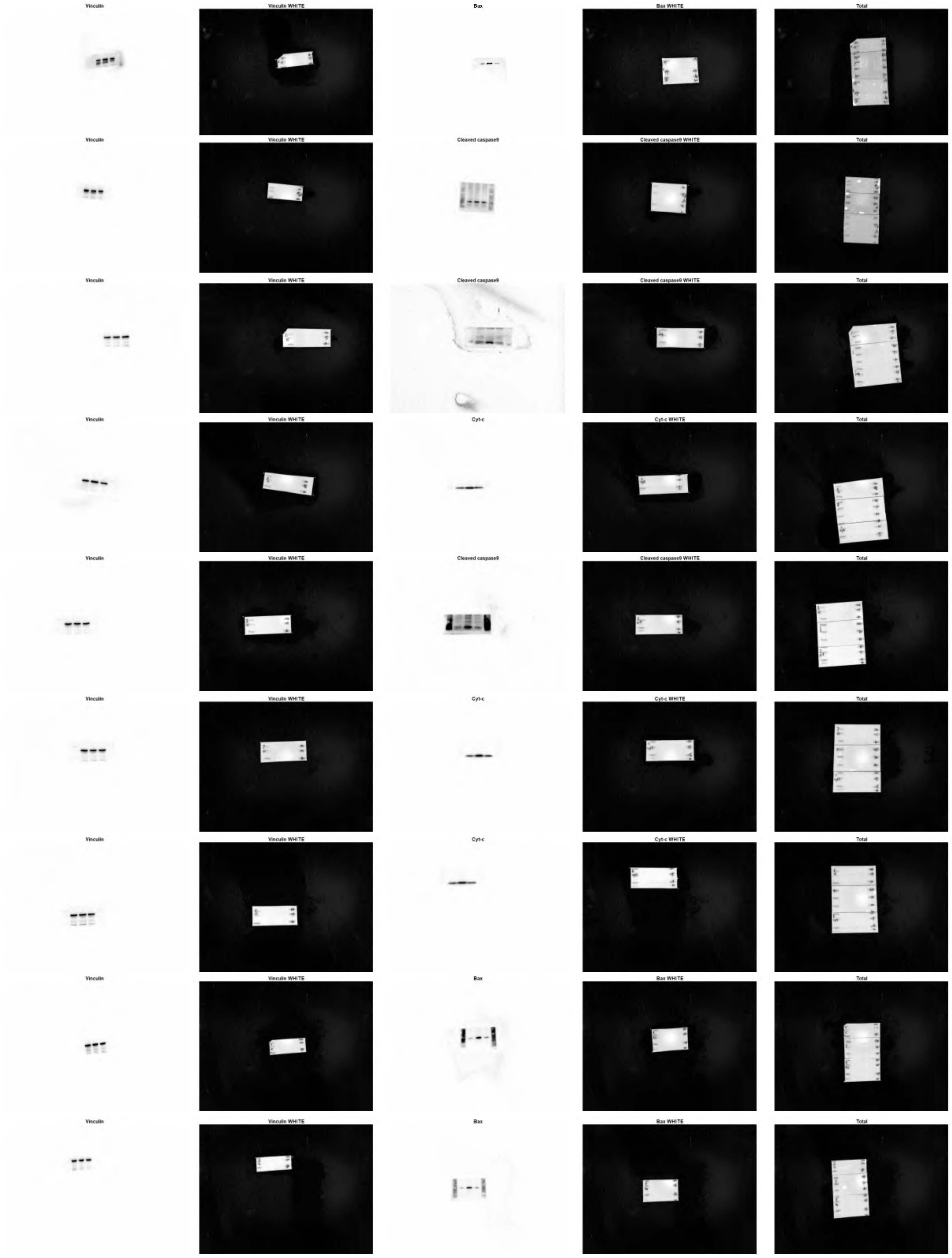

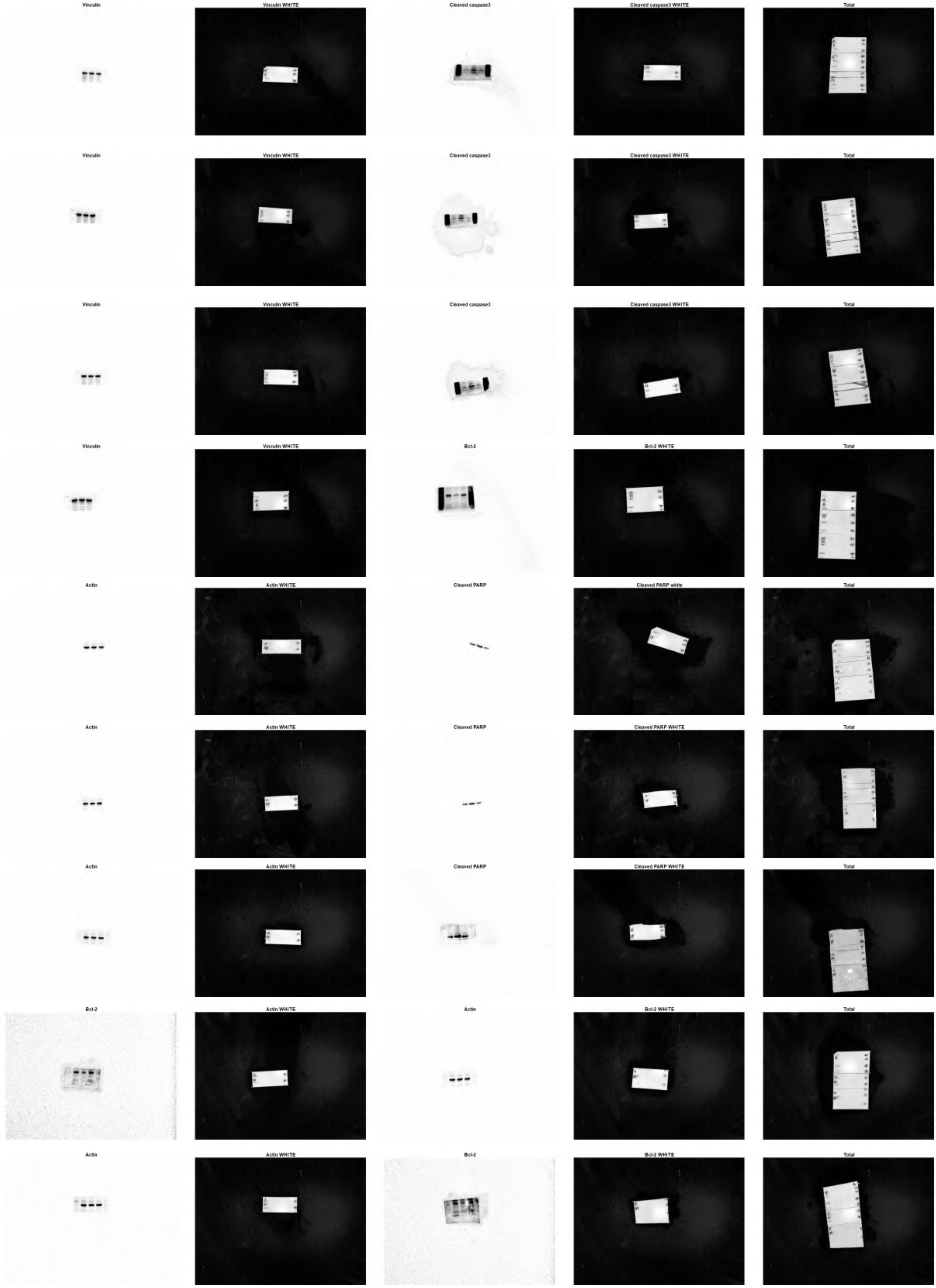
Vinculin
Vinculin WHITE
Cleaved caspase3
Cleaved caspase3 WHITE
Total
Vinculin
Vinculin WHITE
Cleaved caspase3
Cleaved caspase3 WHITE
Total
Vinculin
Vinculin WHITE
Cleaved caspase3
Cleaved caspase3 WHITE
Total
Vinculin
Vinculin WHITE
Bcl-2
Bcl-2 WHITE
Total
Actin
Actin WHITE
Cleaved PARP
Cleaved PARP white
Total
Actin
Actin WHITE
Cleaved PARP
Cleaved PARP WHITE
Total
Actin
Actin WHITE
Cleaved PARP
Cleaved PARP WHITE
Total
Bcl-2
Actin WHITE
Actin
Bcl-2 WHITE
Total
Actin
Actin WHITE
Bcl-2
Bcl-2 WHITE
Total

Supplementary Figure 6H

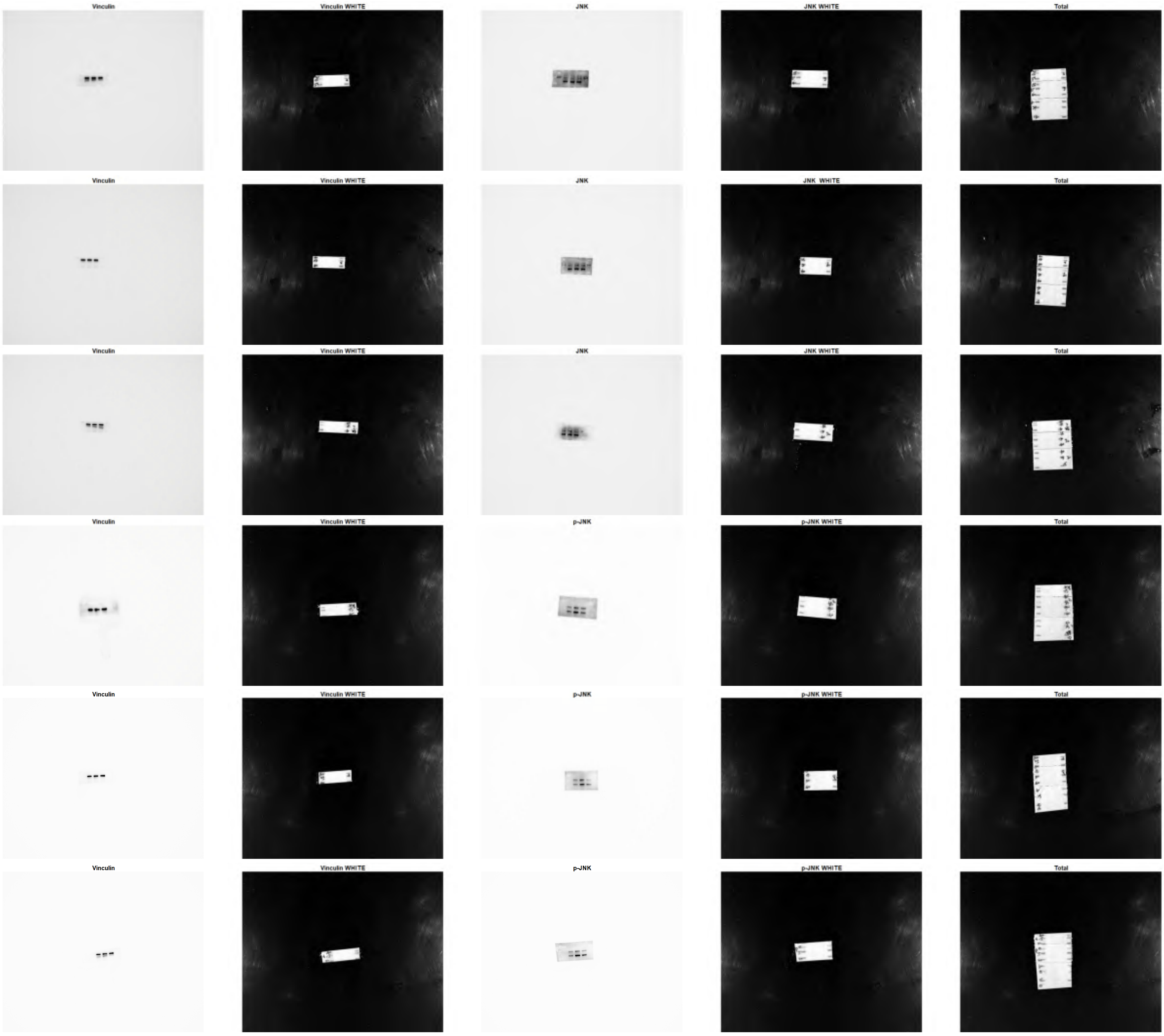

Supplementary Figure7D

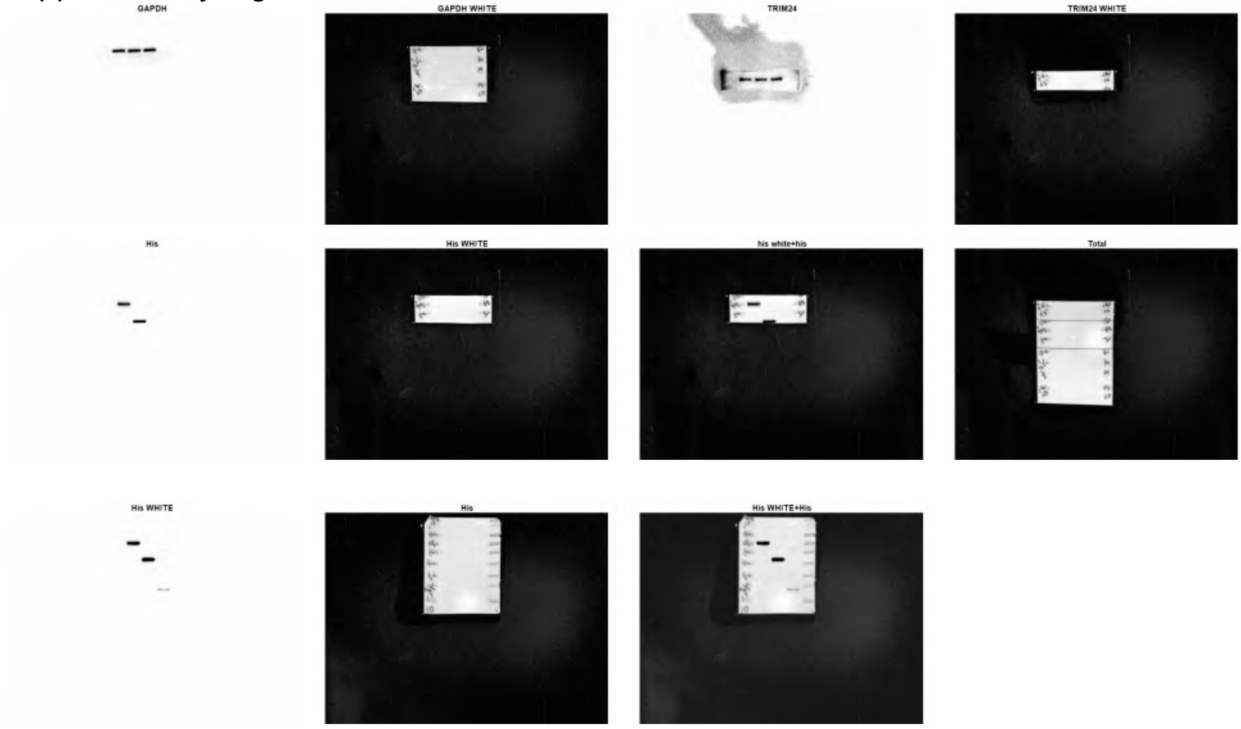

Supplementary Figure7E

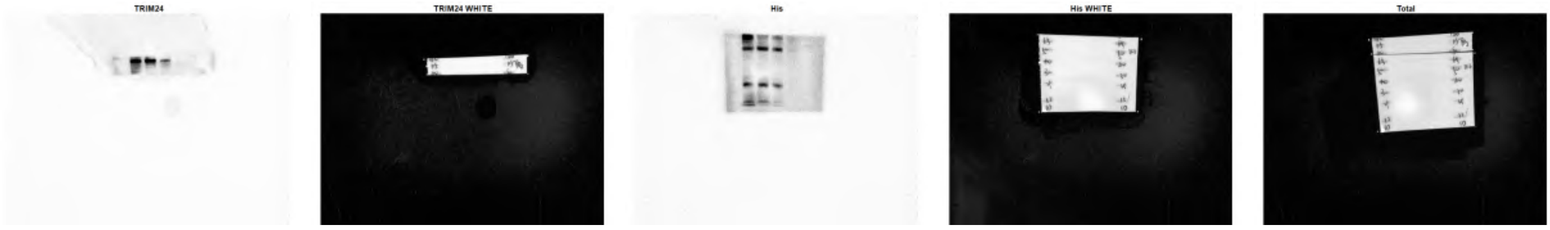

Supplementary Figure8E

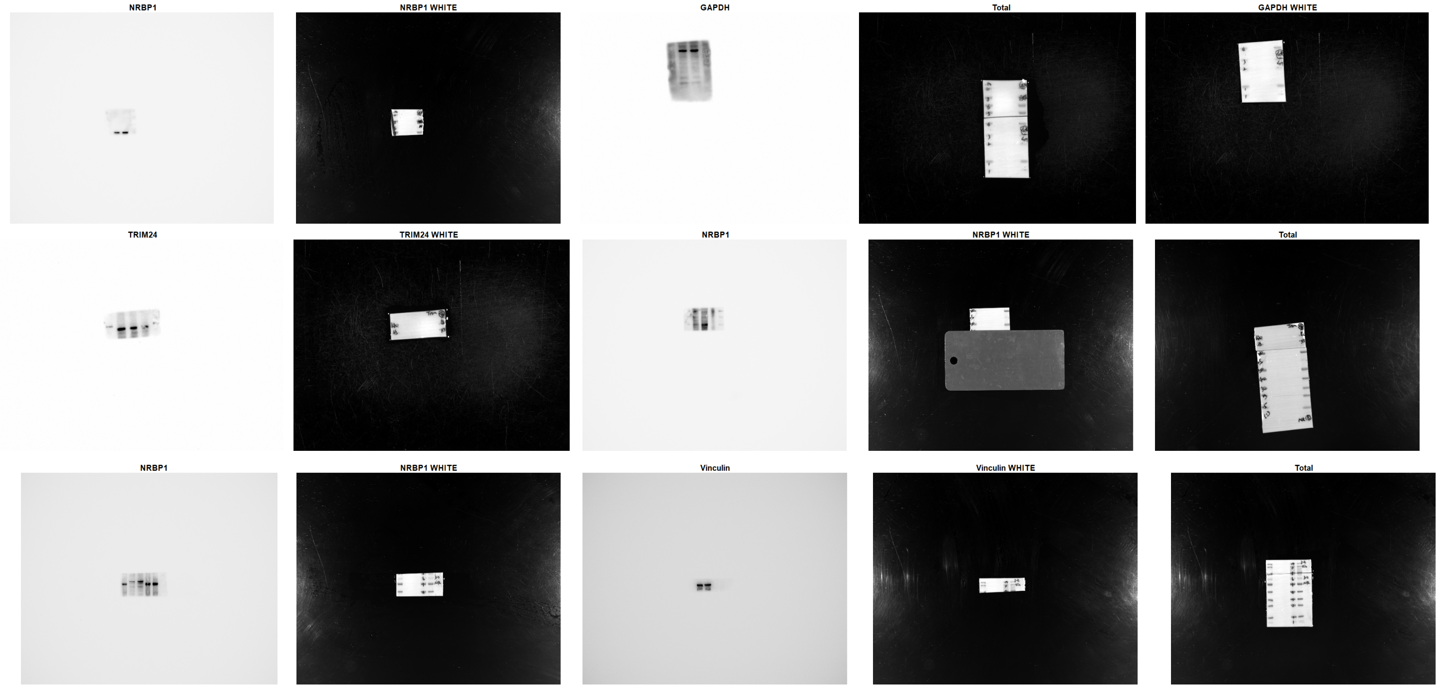

Supplement: Supplementary file 3 — All western blots -supplementary materials [file 41419_2025_8346_MOESM3_ESM.pdf]
